# Molecular basis of SIFI activity in the integrated stress response

**DOI:** 10.1038/s41586-025-09074-z

**Published:** 2025-05-06

**Authors:** Zhi Yang, Diane L. Haakonsen, Michael Heider, Samuel R. Witus, Alex Zelter, Tobias Beschauner, Michael J. MacCoss, Michael Rapé

**Affiliations:** 1 Department of Molecular and Cell Biology, University of California at Berkeley, Berkeley, CA USA; 2 Howard Hughes Medical Institute, University of California at Berkeley, Berkeley, CA USA; 3 Department of Genome Sciences, University of Washington, Seattle, WA USA; 4 California Institute for Quantitative Biosciences (QB3), University of California at Berkeley, Berkeley, CA USA; 5 Present Address: Lunenfeld-Tanenbaum Research Institute, Sinai Health System, Toronto, Ontario Canada; 6 Present Address: Biochemie-Zentrum der Universität Heidelberg (BZH), Heidelberg, Germany

**Keywords:** Ubiquitin ligases, Cryoelectron microscopy, Protein quality control

## Abstract

Chronic stress response activation impairs cell survival and causes devastating degenerative diseases^1–3^. Organisms accordingly deploy silencing factors, such as the E3 ubiquitin ligase silencing factor of the integrated stress response (SIFI), to terminate stress response signalling and ensure cellular homeostasis^4^. How a silencing factor can sense stress across cellular scales to elicit timely stress response inactivation is poorly understood. Here we combine cryo-electron microscopy analysis of endogenous SIFI with AlphaFold modelling and biochemical studies to report the structural and mechanistic basis of the silencing of the integrated stress response. SIFI detects both stress indicators and stress response components through flexible domains within an easily accessible scaffold, before building linkage-specific ubiquitin chains at separate, sterically restricted elongation modules. Ubiquitin handover by a ubiquitin-like domain couples versatile substrate modification to linkage-specific ubiquitin polymer formation. Stress response silencing therefore exploits a catalytic mechanism that is geared towards processing many diverse proteins and therefore allows a single enzyme to monitor and, if needed, modulate a complex cellular state.

## Abstract (web summary)

To efficiently silence the integrated stress response, SIFI detects many diverse substrates that include stress indicators and stress response components through flexible domains within an easily accessible scaffold, before building linkage-specific ubiquitin chains at separate, sterically restricted elongation modules.

## Main

The resilience of metazoan development relies on stress response pathways that mitigate mutational, physiological or environmental challenges to cellular processes^3,5,6^. Defective mitochondrial protein import activates the stress response kinase HRI, which phosphorylates the translation initiation factor eIF2α to stall the synthesis of mitochondrial proteins^7–9^. In this manner, HRI reduces the cytoplasmic load of aggregation-prone proteins and provides cells with time to restore mitochondrial integrity. HRI is one of four kinases of the integrated stress response that enables cells to overcome a wide range of deleterious conditions^1,3,10,11^.

As stress responses put core processes on hold, their prolonged activation can trigger cell death and tissue degeneration^1,2^. To prevent this from happening, cells turn off stress responses using dedicated silencing factors^4^. After restoration of mitochondrial import, the E3 ligase SIFI marks HRI and its activator DELE1 for proteasomal degradation to terminate eIF2α phosphorylation and restart protein synthesis^4^. Deletion of the SIFI subunit UBR4 disrupts heart, brain and yolk-sac development, resulting in embryonic lethality^12,13^, and mutations in *UBR4* cause ataxia and early onset dementia^14–16^. *UBR4*-deficient cells could be rescued by genetic or pharmacological inactivation of HRI^4^, which showed that stress response silencing is critical for cellular homeostasis.

To ensure timely silencing, SIFI must sense whether cells still experience stress. It accomplishes this task by recognizing mitochondrial precursors that accumulate in the cytoplasm only during stress^4,17–19^, and it also detects cleaved proteins that can be released from stressed mitochondria^4,7,8,20^. These proteins compete with HRI for access to SIFI, thereby delaying silencing of the stress response until stress has been resolved^4^. How SIFI can process hundreds of proteins that differ in size from around 50 to 2,500 residues and adopt different conformations is unclear. Indeed, how a single E3 ligase can monitor a global cellular state remains to be elucidated.

Here we combined cryo-electron microscopy (cryo-EM) analyses of SIFI with AlphaFold (AF) modelling and biochemical studies to reveal the molecular basis of mitochondrial stress response silencing. SIFI processes its many substrates using flexible domains within an easily accessible scaffold, while it builds Lys48-linked ubiquitin (Ub) polymers at separate modules centred on a sterically restricted E2. SIFI’s ubiquitylation centres communicate through ubiquitin handover orchestrated by a ubiquitin-like (UBL) domain. Silencing of the integrated stress response therefore relies on a mechanism that couples versatile substrate modification to linkage-specific chain elongation, which allows a single enzyme to process many diverse proteins for timely stress response silencing.

## Structure of endogenous SIFI

**Fig. 1:**
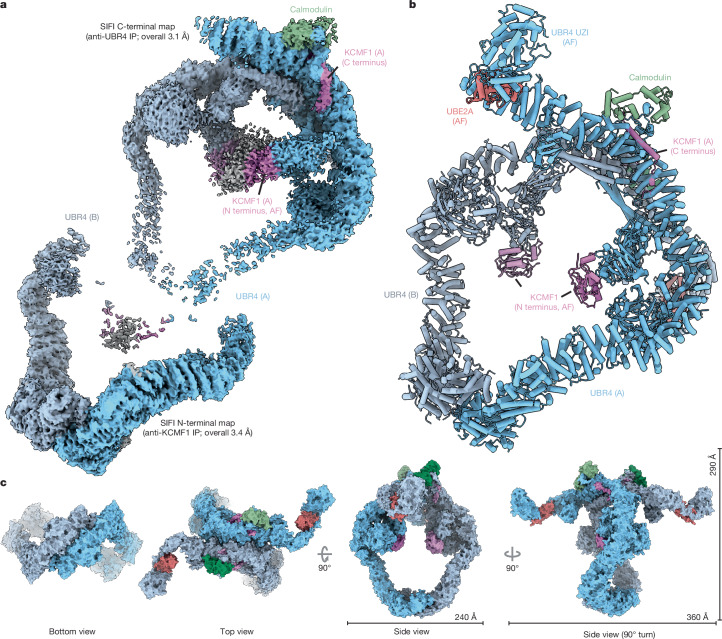
Cryo-EM structure of human SIFI. **a**, Cryo-EM maps of SIFI, highlighting its C-terminal partial map (endogenous SIFI complex; top left; Electron Microscopy Data Bank (EMDB): EMD-46686, contour level 0.12) and N-terminal partial map (immunoprecipitation (IP) of KCMF1; bottom left; EMDB: EMD-46688, contour level 0.07). (A) and (B) denote the two heterotrimers. **b**, Composite structure of human SIFI built from partial maps (Protein Data Bank (PDB): 9D9Z), including two subunits each of UBR4 (blue), KCMF1 (purple), calmodulin (green) and UBE2A (red). **c**, Surface representations of the SIFI complex viewed from the N- and C-terminal dimerization regions, as well as views from above and the side of ring’s plane. All components are colour coded as in **a** and **b**.

**Extended Data Fig. 1:**
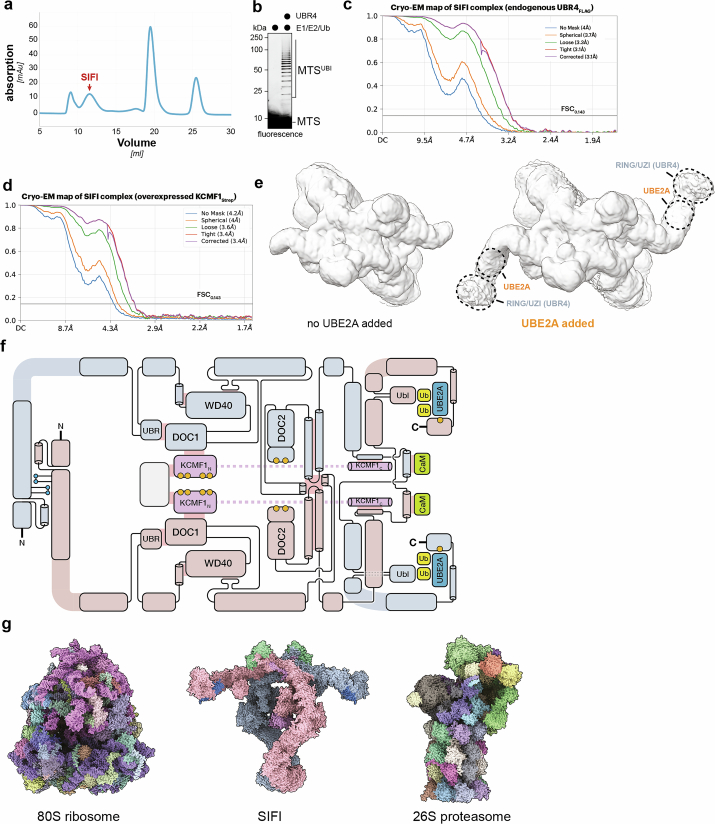
Structural characterization of human SIFI. **a**. Size exclusion chromatography of affinity-purified endogenous UBR4^FLAG^. Red arrow denotes the SIFI peak, confirmed by Western blotting and negative stain EM (data not shown). **b**. In vitro ubiquitylation of a fluorescently labelled presequence peptide shows that SEC purified endogenous SIFI used for cryo-EM is active in catalysing ubiquitylation. Similar results in n = 2 independent experiments. **c**. Fourier shell correlation (FSC) plot of the cryo-EM map of endogenous SIFI complexes. Resolution cutoff for gold-standard FSC_0.143_ is shown. **d**. FSC plot of the cryo-EM map of SIFI complexes purified by affinity-purification of overexpressed KCMF1^Strep^. **e**. Comparison of the cryo-EM maps of SIFI (left. EMD-46686, Gaussian filter Width=2.0, contour level 0.04) and SIFI supplemented with recombinant UBE2A (right. EMD-46742, Gaussian filter Width=2.0 contour level 0.014) reveals stabilization of the C-terminal region of UBR4, including hemi-RING/UZI domain. **f**. Schematic representation of the SIFI complex. **g**. Comparison between SIFI, 26S proteasome (PDB 6MSB), and 80S ribosome (PDB 6QZP) reveals their similar size dimensions. For gel source data, see Supplementary Fig. 1.

For structure determination, we purified endogenous SIFI by immunoprecipitating its largest subunit, UBR4, and then performing size-exclusion chromatography (Extended Data Fig. 1a). Having ensured that SIFI was active (Extended Data Fig. 1b), we used single-particle cryo-EM to resolve a partial C-terminal map of UBR4 and associated proteins to an overall resolution of 3.1 Å (Fig. 1a, Extended Data Fig. 1c and Supplementary Fig. 3). Purification of SIFI through its subunit KCMF1 led to a complementary map of the N-terminal half of UBR4 at an overall resolution of 3.4 Å (Fig. 1a and Extended Data Fig. 1d). The C-terminal region of UBR4, which includes its hemi-RING^21^, was not resolved until the SIFI E2 enzyme UBE2A was supplemented before cryo-EM analysis (Extended Data Fig. 1e). We integrated AF models of specific domains into less-well-resolved regions and combined all of the maps to build a near-complete structural model of human SIFI (Fig. 1b,c, Extended Data Table 1, Supplementary Video 1 and Supplementary Fig. 3).

SIFI forms an antiparallel dimer of heterotrimers that comprises two copies each of the E3 ligase UBR4, the E3 ligase KCMF1 and calmodulin^22^ (Fig. 1b,c and Extended Data Fig. 1f). UBE2A was captured by the C-terminal region of each UBR4 subunit that became ordered in its presence. Together, these proteins assemble into an approximately 1.3 MDa structure that is built around an open twisted-ring scaffold (292 Å × 229 Å) and two arms that extend to the side and span about 356 Å. SIFI is of comparable size to 80S ribosomes and 26S proteasomes (Extended Data Fig. 1g).

**Extended Data Fig. 2:**
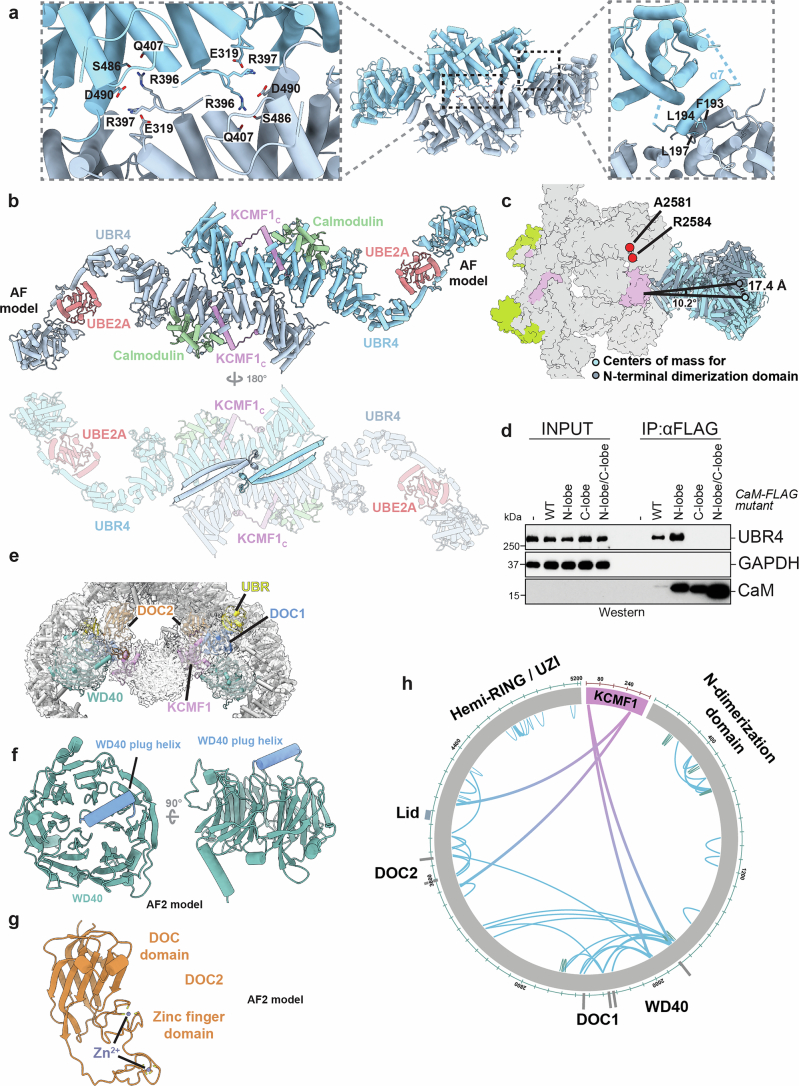
Structural domains of SIFI. **a**. Structural insights into the N-terminal dimerization region of SIFI. *Left*: two Lys residues at the tips of interlocking loops from each protomer engage in polar interactions with their paired counterpart. *Right*: α7 helix of UBR4 undergoes a helix swap with the paired protomer to form a helical bundle. **b**. C-terminal dimerization region. 180° rotation reveals the stabilizing helical pair at the inside of the SIFI scaffold. **c**. The SIFI scaffold shows flexibility around a central hinge region, leading to ~10° rotations of the N- and C-terminal dimerization domains relative to each other. Two disease mutations in *UBR4*, A2581 (ataxia) and R2584 (cancer) are located close to the hinge region. **d**. Calcium-binding to the C-lobe, but not the N-lobe of calmodulin is required for binding of UBR4, as seen by immunoprecipitation of calmodulin mutants that disrupt calcium-binding in the N- or C-lobe, respectively (N-lobe mutant: D21A, D23A, D57A, D59A; C-lobe mutant: D94A, D96A, D130A, D132A)^28^. Similar results in n = 2 independent experiments. **e**. Cryo-EM map of central SIFI showing docked WD40, DOC and UBR domains. These internal protein interaction modules are flexible and hence show lower overall resolution. The central density (ABHD10) is described below. **f**. The WD40 domain of UBR4 features a central plug helix at a surface commonly involved in protein interactions characteristic of WD40 repeats. **g**. The DOC2 domain of UBR4 contains two Zinc-binding loops that collectively form a potential protein interaction interface. **h**. Diagram of cross-linking mass spectrometry validates domain arrangement at the centre of SIFI. For gel source data, see Supplementary Fig. 1.

SIFI’s scaffold is primarily composed of α-helical armadillo repeats of UBR4 and relies on antiparallel dimer interfaces at its N- and C-terminal regions with buried surface areas of around 2,160 Å^2^ and 4,212 Å^2^, respectively (Fig. 1b,c). The N-terminal dimer interface is mediated by a domain-swapped helix from one protomer that bundles with three helices of the second, as well as two Arg residues that interlock the dimer through polar interactions (Extended Data Fig. 2a). The C-terminal interface is centred on extended interactions between helical repeats from each protomer that are cemented by horizontal coiled-coils at the inner side of the scaffold (Extended Data Fig. 2b). Revealing flexibility of the scaffold, the dimerization interfaces show ~10° rotations towards each other, which occur around a hinge close to UBR4 Ala2581, which is mutated in ataxia, and Arg2584, which is altered in cancer^23,24^ (Extended Data Fig. 2c and Supplementary Video 2).

**Fig. 2:**
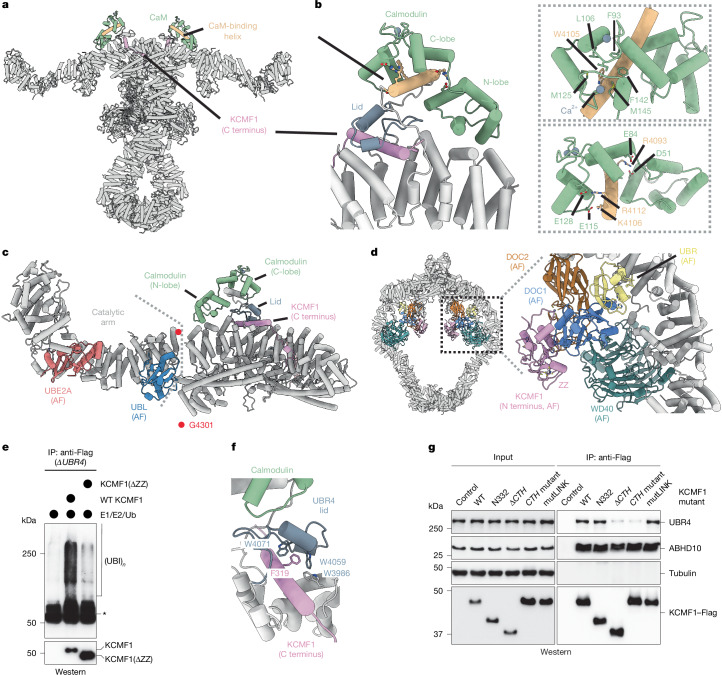
Structural arrangements of SIFI modules. **a**, Calmodulin (green; CaM) binds to the outer rim of the SIFI scaffold above the C-terminal UBR4 dimerization interface. **b**, The N-terminal lobe of calmodulin (green) forms a helix bundle with UBR4 (light grey), while the Ca^2+^-bound C-terminal lobe of calmodulin engages a calmodulin-binding motif of UBR4 (gold), situated atop the UBR4 lid (blue grey) that encircles the C-terminal α-helix of KCMF1 (plum). Conserved residues involved in the interaction are highlighted within dashed boxes. **c**, Calmodulin stabilizes the base of the UBR4 C-terminal catalytic arm, including hemi-RING, UBE2A (coral) and UBL (blue), allowing flexible movement of the arm around residue Gly4301 (red dot) of UBR4. **d**, Two copies each of SIFI’s protein interaction modules (coloured) are located at the centre of the scaffold (left). Right, magnified view of the DOC2 (brown) and WD40 (teal) domains, and the KCMF1^N138^–DOC1–UBR subcomplex (plum, blue and yellow). **e**, The N-terminal ZZ domain of KCMF1 is required for ubiquitylation activity. WT or ΔZZ KCMF1 were purified from *ΔUBR4* cells to prevent UBR4-dependent ubiquitylation and incubated with E1, E2 (UBE2D3, UBE2A) and ubiquitin. Activity was detected by formation of high-molecular-mass conjugates using anti-ubiquitin western blotting. Ubiquitylated species (UBI). Similar results were observed in *n* = 2 experiments. **f**, C-terminal helix of KCMF1 (plum) is anchored within α-helical bundles of UBR4’s armadillo repeats (light grey) through the UBR4 lid (blue grey). A conserved Phe319 of KCMF1 (plum), surrounded by three Trp residues of UBR4, is highlighted. **g**, Deletion or mutation (R316E/F319E/L323E/L325E) of the C-terminal helix (CTH mutant) impedes KCMF1 integration into SIFI, as shown by KCMF1–Flag affinity purification and detection of UBR4. A Q226/228/229/231/233E mutation in an unresolved linker of KCMF1 (mutLINK) did not affect integration into SIFI. ABHD10 binds to the ZZ domain of KCMF1 and was used as a positive control. Similar results were observed in *n* = 2 experiments. Gel source data are provided in Supplementary Fig. 1.

Calmodulin docks onto the outer rim of the scaffold, close to a hinge around UBR4 Gly4301 that connects the C-terminal dimer interface to the catalytic module of UBR4 (Fig. 2a–c). The C-lobe of calmodulin, which is bound to calcium, captures an exposed UBR4 helix that fits the calmodulin-binding consensus and contains Arg residues mutated in cancer and ataxia^24–26^ (Fig. 2a,b). Hydrophobic calmodulin residues surround Trp4105 of UBR4 in a manner consistent with other calcium-activated structures^26,27^, and mutation of calcium-binding residues in the C-lobe^28^ disrupted UBR4 binding (Extended Data Fig. 2d). Calmodulin’s N-lobe forms a bundle with two helices of the UBR4 scaffold (Fig. 2b,c). The N-lobe is not bound to calcium, and calcium-binding residues are not required for UBR4 recognition (Extended Data Fig. 2d). By engaging two distinct regions of UBR4, calmodulin creates an intramolecular bridge that stabilizes the UBR4 lid to anchor KCMF1 to the scaffold, as described below. These findings may explain why deletion of UBR4 residues that include its exposed helix blocked calmodulin integration into SIFI and prevented stress response silencing^4,29^.

Tethered to the scaffold’s interior are the UBR box, the WD40 repeat and two DOC-homologous β-sandwich domains of each UBR4 subunit (Figs. 1b and 2d). Their lower local resolution indicates that these modules are flexibly attached to the scaffold (Extended Data Fig. 2e). The WD40 repeats reside symmetrically on either side of the ring, where they are supported by a helix protruding from the scaffold. A second short helix is predicted to plug each β-propeller using loops that often recognize binding partners of WD40 repeats (Extended Data Fig. 2f). The DOC1 domains are positioned near each β-propeller and engage the UBR box of UBR4 and the N-terminal region of KCMF1 to form a three-component subcomplex, referred to as the KCMF1 module, that fits well into the local ~16 Å map (Fig. 2d). Indicative of its inherent flexibility, 3D classification analyses indicated that this subcomplex can adopt distinct positions on SIFI (Supplementary Video 1). The DOC2 domains reside next to the KCMF1 module, where they combine a β-sandwich characteristic of DOC domains with two exposed Zn^2+^-coordinating loops to form a potential protein-binding interface (Extended Data Fig. 2g). Cross-linking mass spectrometry (MS) confirmed the position of these interaction modules within the centre of SIFI (Extended Data Fig. 2h).

**Extended Data Fig. 3:**
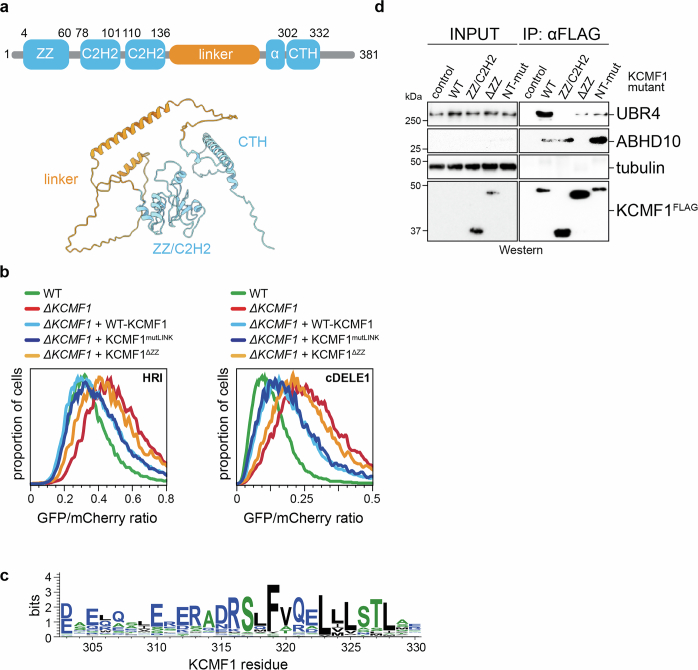
KCMF1 recruitment and function in SIFI. **a**. *top:* Schematic of domain arrangement in KCMF1. *bottom:* KCMF1 AlphaFold model suggests that the KCMF1 linker not observed in SIFI structures (orange) does not adopt a compact fold. **b**. The ZZ-domain of KCMF1, but not its linker, is required for substrate degradation. Stability of HRI or cleaved DELE1 was measured in *ΔKCMF1* cells using flow cytometry. As indicated, WT-KCMF1, KCMF1^ΔZZ^, or KCMF1^mutLink^ (containing 5 Q/E mutations in conserved residues), were expressed. Similar results in n = 2 independent experiments. **c**. The central Phe residue in the C-terminal KCMF1 helix is highly conserved. **d**. Deletion of the N-terminal ZZ domain or mutation of R20E, E38R, L61E, Y64E, R68E in the ZZ domain strongly impaired the interaction between KCMF1^FLAG^ and endogenous UBR4, as shown by affinity-purification and Western blotting. The ZZ deletion also interfered with recognition of the N-degron substrate ABHD10, as described below. Similar results in n = 2 independent experiments. For gel source data, see Supplementary Fig. 1.

SIFI possesses two subunits, KCMF1 and UBR4, with unconventional E3 ligase motifs. In KCMF1, we found that the N-terminal ZZ domain, which shares structural similarity to RING domains, supports ubiquitylation (Fig. 2e). This domain and two predicted C2H2-type zinc fingers are near the centre of the scaffold, where they bind to the DOC1 domain of UBR4, which has been shown to be required for KCMF1 binding^4^ (Fig. 2d). Following an unresolved linker that is predicted to adopt a flexible conformation and of which mutation impacted neither SIFI binding nor substrate degradation (Fig. 2g and Extended Data Fig. 3a,b), KCMF1 is further anchored above the C-terminal UBR4 dimer interface through a helix that is enclosed by the UBR4 lid (Fig. 2f). The UBR4 lid engages the KCMF1 helix through extensive van der Waals and polar interactions, including a group of Trp residues that encircle the conserved Phe319 of KCMF1 (Fig. 2f and Extended Data Fig. 3c). Underscoring the importance of these features, mutation or deletion of the N- or C-terminal regions of KCMF1 impaired its retention in SIFI (Fig. 2g and Extended Data Fig. 3d).

The ubiquitylation modules of UBR4 are found within the peripheral SIFI arms that make an approximately 90° turn to extend about 166 Å above the scaffold^21^ (Fig. 1b,c). These catalytic centres include a ten-turn α-helix, a hemi-RING and a UZI domain in which several ataxia mutations are found^16,24^. On the basis of X-ray structures and AF models^21^, the α-helix binds to the backside of the E2 and, together with the hemi-RING, captures UBE2A in a tight embrace that probably explains why this region was only visible when SIFI was incubated with its E2. Close to this module is a UBL domain that is tethered to the UBR4 scaffold through long linkers rich in Asp and Glu residues (Fig. 2c). As described below, the UBL domain has an important role in ubiquitin chain formation.

Together, our structural analyses reveal that SIFI contains several protein interaction modules within a flexible and easily accessible scaffold. The N-terminal, RING-like domains of its two KCMF1 subunits are located at the centre of this structure, while two additional ubiquitylation modules reside within the peripheral UBR4 arms. With four catalytic modules and a dynamic architecture, SIFI appears to be optimally configured to process many substrates for efficient stress response silencing.

## Substrates bind at the SIFI centre

**Fig. 3:**
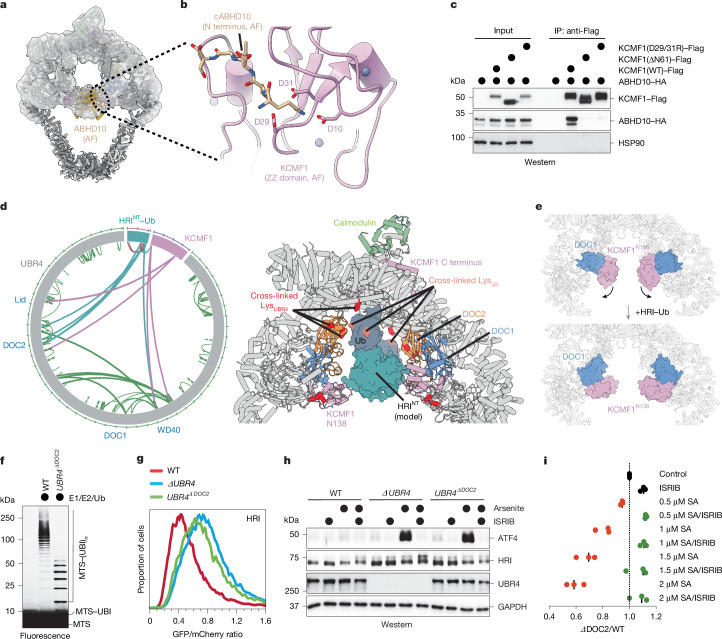
SIFI binds to substrates at the centre of its twisted-ring scaffold. **a**, Overview of SIFI, highlighting a low-resolution density ascribed to ABHD10 dimers, with an ABHD10 AF2 model fitted in the map (Gaussian filter width 2.0, contour 0.052). **b**, AF2 model of ABHD10’s cleaved N terminus (cABHD10) fitting into the N-degron-binding pocket within the ZZ domain of KCMF1. **c**, ZZ-domain deletion or Asp29/Asp31 mutation in KCMF1 ablates recognition of ABHD10–HA, as seen by KCMF1–Flag immunoprecipitation from *ΔUBR4* HEK293T cells. The experiment was performed once. **d**, Diagram of cross-linking MS analysis of the SIFI–HRI–Ub complex, highlighting cross-linked residues from UBR4, KCMF1 and HRI–Ub (left). Right, model of dimerized HRI–Ub placed at SIFI’s central cavity, with HRI located near to the DOC2 and KCMF1^N138^ domains, and fused ubiquitin near its cross-linked regions (red, salmon: cross-linked residues determined by MS). **e**, Conformational change of the KCMF1^N138^–DOC1–UBR subcomplex after SIFI binding to HRI^NT^. **f**, SIFI was purified from WT or ΔDOC2 *UBR4* cells and incubated with a fluorescently labelled MTS peptide, E1, E2 (UBE2D3 and UBE2A) and ubiquitin. Similar results were observed in *n* = 3 experiments. **g**, Deletion of the DOC2 domain in endogenous *UBR4* stabilizes HRI to a similar extent to complete *UBR4* inactivation. HRI stability was assessed by flow cytometry after *HRI-GFP::mCherry* expression. Similar results were observed in *n* = 3 experiments. **h**, DOC2 deletion leads to integrated stress response hyperactivation after mitochondrial stress induced by sodium arsenite (SA), as seen by induction of ATF4 using western blotting. Similar results were observed in *n* = 2 experiments. **i**, *UBR4*^*ΔDOC2*^ cells are hypersensitive to sodium-arsenite-induced stress, dependent on the integrated stress response. As indicated, the integrated stress response was inhibited using ISRIB. The fitness of GFP-labelled WT and mCherry-labelled *UBR4*^*ΔDOC2*^ cells was assessed through cell competition. *n* = 3 biologically independent replicates are presented together with the median of these experiments. Gel source data are provided in Supplementary Fig. 1.

**Extended Data Fig. 4:**
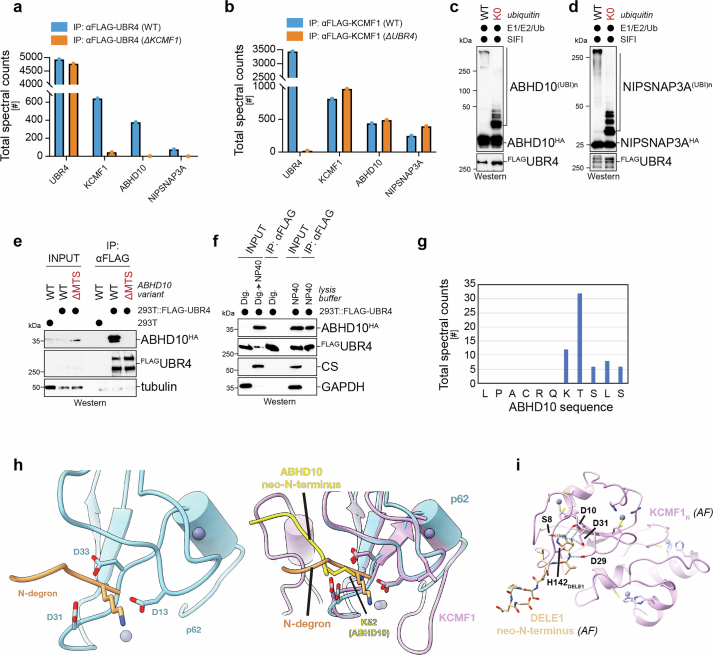
ABHD10 binds SIFI as an N-degron substrate. **a**. Immunoprecipitation of SIFI in WT or *ΔKCMF1* cells coupled to mass spectrometry shows that two mitochondrial proteins, ABHD10 and NIPSNAP3A, are lost from SIFI purifications in the absence of KCMF1. **b**. Immunoprecipitation of KCMF1 coupled to mass spectrometry from WT or *ΔUBR4* cells shows that KCMF1 binds to ABHD10 and NIPSNAP3A independently of UBR4. **c**. SIFI-bound ABHD10 is ubiquitylated if SIFI is incubated with E1, UBE2D3/UBE2A, and ubiquitin, as shown by Western blotting. Ubiquitin lacking Lys residues and hence unable to support chain formation (K0) is used as control. Similar results in n = 2 independent experiments. **d**. SIFI-bound NIPSNAP3A is ubiquitylated by SIFI in a similar manner as ABHD10. Experiment performed once. **e**. ABHD10 only binds KCMF1 if it contains an N-terminal presequence that is cleaved off upon entry into mitochondria. ΔMTS-ABHD10 does not contain its presequence and is not imported into mitochondria, thus initiating with a Met residue. Binding of ABHD10^HA^ to KCMF1^FLAG^ was analysed by αFLAG-affinity-purification and Western blotting. Similar results in n = 2 independent experiments. **f**. ABHD10 only binds KCMF1 after being released from mitochondria. Cells were either lysed with digitonin, which preserves mitochondrial integrity, or NP40, which disrupts mitochondrial integrity and results in release of mitochondrial proteins into the lysate. UBR4^FLAG^ was affinity-purified and bound ABHD10^HA^ was detected by Western blotting. CS: citrate synthase. Similar results in n = 2 independent experiments. **g**. Cleaved ABHD10 likely starts with a Lys residue, as determined by surveying all ABDH10-peptides retrieved in ABHD10 immunoprecipitations by mass spectrometry. **h**. *Left:* Structure of the ZZ-domain of p62, which is known to bind to N-degrons as determined by X-ray crystallography (PDB 5YPS). *Right:* overlay of p62 and KCMF1 N-domains bound to their respective N-degrons. **i**. AlphaFold model of cleaved DELE1 bound by the ZZ-domain of KCMF1. For gel source data, see Supplementary Fig. 1.

To prevent premature stress response silencing, SIFI must survey an entire cell to detect whether stress is still present. To this end, SIFI processes unimported or cleaved proteins that only accumulate in the cytoplasm during stress^4^, but how it engages its diverse substrates is unclear. In the cryo-EM map of endogenous SIFI, we noticed a low-resolution density that was attached to KCMF1 and could not be ascribed to SIFI components (Fig. 3a). To identify this factor, we compared SIFI immunoprecipitates from wild-type (WT) and *ΔKCMF1* cells using MS and found that two mitochondrial proteins, ABHD10 and NIPSNAP3A, were lost in the absence of KCMF1 (Extended Data Fig. 4a and Supplementary Table 1). The same proteins were found to bind to KCMF1 in *ΔUBR4* cells (Extended Data Fig. 4b and Supplementary Table 1). AF models showed that ABHD10 dimers fit well into the central density of the cryo-EM map (Fig. 3a). Both ABHD10 and NIPSNAP3A were ubiquitylated by SIFI (Extended Data Fig. 4c,d), showing that these proteins were substrates, rather than subunits, of the E3 ligase.

SIFI recognized only ABHD10 variants that could be imported into mitochondria (Extended Data Fig. 4e). Instead of detecting the presequence, SIFI bound to ABHD10 that was released from mitochondria during cell lysis and possessed a new N terminus resulting from cleavage by mitochondrial presequence peptidase (Extended Data Fig. 4f). MS analyses of SIFI-bound ABHD10 revealed an N-terminal lysine (Extended Data Fig. 4g and Supplementary Table 1), a residue known to act as an N-degron^30^. SIFI recognized cleaved ABHD10 through the ZZ domain of KCMF1 (Fig. 3a,b and Extended Data Fig. 3d), which shows structural similarity to a domain in p62 that binds to N-degrons^31^ (Extended Data Fig. 4h). AF models showed that the processed ABHD10 N-termini fit well into the N-degron pocket of KCMF1 (Fig. 3b), which was validated by mutation of Asp residues in KCMF1 that ablated ABHD10 recognition (Fig. 3c). The fortuitous co-purification of ABHD10 dimers, which can access both ZZ domains at the centre of SIFI and therefore stably interact with the E3 ligase, showed how SIFI detects a set of N-degron targets. Note that DELE1 contains an N-degron that is produced by stress-induced cleavage through OMA1^4^ and, as indicated by AF, fits well into the N-degron pocket of KCMF1 (Extended Data Fig. 4i).

**Extended Data Fig. 5:**
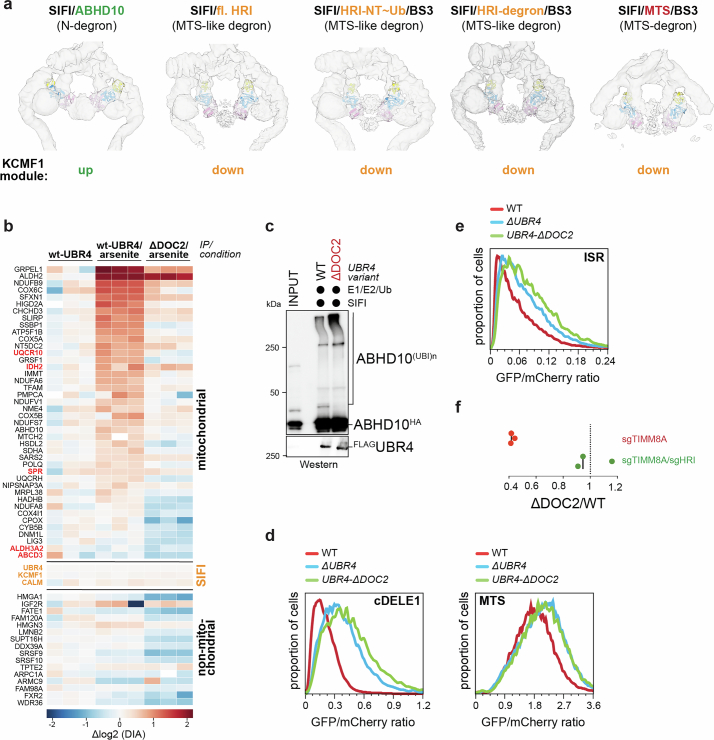
The DOC2 domain of UBR4 is important for SIFI function. **a**. Crosslinking of HRI~Ub or a presequence peptide to SIFI results in similar conformational rearrangements of the KCMF1 module composed of the N-terminal domains of KCMF1 and the DOC1 and UBR domains of UBR4, as determined by cryo-EM. fl: full-length; MTS: mitochondrial targeting sequence. All maps were Gaussian filtered with width sdev=2.0. **b**. SIFI binds endogenous mitochondrial proteins, when import has been disrupted by arsenite treatment. When SIFI was purified from cells expressing endogenous UBR4^ΔDOC2^, the binding of mitochondrial proteins was lost. Binding partners were determined after endogenous SIFI affinity-purification using mass spectrometry. Mitochondrial proteins, for which predicted presequence peptides could be identified, are shown in red. IP-MS samples were run as technical triplicates. **c**. The DOC2 domain of SIFI is not required for ABHD10 ubiquitylation. SIFI was purified from either WT or *UBR4*^ΔDOC2^ cells that also expressed ABHD10^HA^ and incubated with E1, UBE2D3/UBE2A, and ubiquitin. Ubiquitylation of bound ABDH10 was detected by Western blotting. Experiment performed once. **d**. The DOC2 domain of SIFI is required for degradation of DELE1 or unimported mitochondrial proteins. The stability of DELE1 or the presequence of COX8A was determined in WT or *UBR4*^ΔDOC2^ cells by flow cytometry, as described before. Similar results in n = 2 independent experiments. **e**. Deletion of the DOC2 domain in *UBR4* results in stronger activation of the integrated stress response. A reporter for integrated stress response activation based on uORFs of ATF4 was expressed in either WT, *ΔUBR4*, or *UBR4*^ΔDOC2^ cells, which were treated with sodium arsenite and analysed by flow cytometry. Similar results in n = 2 independent experiments. **f**. The DOC2 domain of UBR4 is essential for cell survival upon mitochondrial import stress. WT and *UBR4*^ΔDOC2^ cells were labelled with GFP and mCherry, respectively, mixed, and depleted of either TIMM8A or TIMM8A/HRI, as indicated. After 12 d of co-culture, the ratio between cell types was determined by flow cytometry. n = 3 independent replicates shown with the median of these experiments. For gel source data, see Supplementary Fig. 1.

To understand the recognition of HRI and mitochondrial presequences, we incubated SIFI with the degron-containing N-terminal domain of HRI (HRI^NT^)^4^. To increase substrate solubility and provide reactive Lys residues for cross-linking, we fused HRI^NT^ to ubiquitin (HRI^NT^–Ub). Cross-linking MS indicated that SIFI recognizes HRI^NT^–Ub at the centre of its scaffold, close to the DOC2 domain and the C-terminal dimer interface of UBR4 (Fig. 3d). The binding of HRI^NT^–Ub caused a substantial shift in the position of the KCMF1 module, while the density ascribed to ABHD10 was strongly reduced (Fig. 3e and Extended Data Fig. 5a). Reflecting the converging degrons in HRI and unimported mitochondrial proteins^4^, we observed similar conformational changes if SIFI was cross-linked to full-length HRI immunoprecipitated from cells or degron peptides derived from either HRI or mitochondrial presequences (Extended Data Fig. 5a). The architectural rearrangements caused by HRI or presequence binding underscore the flexibility of the central substrate-binding scaffold of SIFI (Supplementary Video 3). Although the resolution of these structures did not permit us to visualize the respective degrons, these results suggested that HRI and mitochondrial presequences bind close to UBR4’s DOC2 domain, and therefore at a different site compared with ABHD10.

To assess the role of UBR4’s DOC2 domain in stress response silencing, we excised it from all *UBR4* loci. Affinity purification coupled to MS revealed that SIFI(ΔDOC2) was unable to engage mitochondrial proteins, including those with an MTS, during stress (Extended Data Fig. 5b and Supplementary Table 1). The binding of UBR4 to KCMF1 or calmodulin was not affected by the DOC2 deletion. SIFI(ΔDOC2) was strongly impaired in catalysing the ubiquitylation of mitochondrial presequences (Fig. 3f), while it retained its ability to modify the N-degron substrate ABHD10 (Extended Data Fig. 5c). Accordingly, the DOC2 deletion stabilized HRI, DELE1 and mitochondrial precursors to a similar extent to *UBR4* inactivation (Fig. 3g and Extended Data Fig. 5d), resulting in increased stress signalling (Fig. 3h and Extended Data Fig. 5e). SIFI(ΔDOC2) cells also showed a strong fitness defect when experiencing mitochondrial stress, which was rescued by HRI deletion or treatment with the stress response inhibitor ISRIB (Fig. 3i and Extended Data Fig. 5f). The DOC2 domains at the centre of SIFI therefore have an important role in degrading HRI and mitochondrial precursors to ensure stress response silencing.

**Extended Data Fig. 6:**
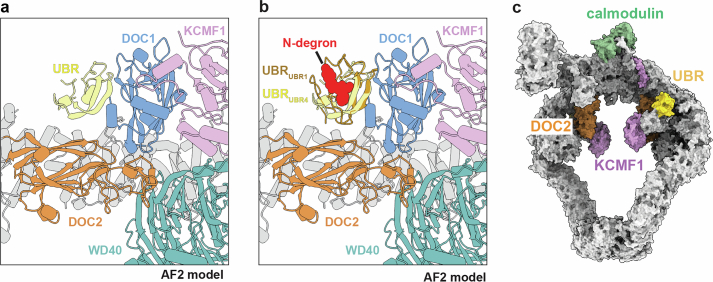
The UBR boxes of UBR4 present substrates towards the interior of SIFI. **a**. The UBR box of UBR4 binds to DOC1 domain of UBR4 and the N-terminal domain of KCMF1, forming the KCMF1^N138^-DOC1-UBR subcomplex. X-ray structure of the UBR box of UBR1 (PDB 3NIH)^35^ reveals that its peptide binding groove is open. **b**. Modelling a N-degron peptide into the open substrate-binding groove of the UBR box of UBR4, based on the UBR1 X-ray structure (PDB 3NIH). **c**. Structure of SIFI showing the proximity of UBR box to other substrate-binding modules of SIFI, including the DOC2 domain of UBR4 and the ZZ domain of KCMF1.

SIFI also contains a UBR box that interacts with N-degron peptides, albeit with comparably low affinity^32–35^. The UBR box in UBR4 is not required for HRI or DELE1 degradation, and it does not contribute to stress response silencing^4^. While the UBR box was not occupied in our structure (Extended Data Fig. 6a), its peptide-binding pocket is accessible (Extended Data Fig. 6b) and would place targets close to those bound through the ZZ and DOC2 domains (Extended Data Fig. 6c). While further studies are needed to investigate proteins processed through SIFI’s UBR box, our analyses suggest that SIFI recognizes all known targets at the centre of its flexible scaffold, where they occupy overlapping space and could therefore compete with DELE1 and HRI to ensure timely stress response silencing.

## Distinct modules for chain formation

**Fig. 4:**
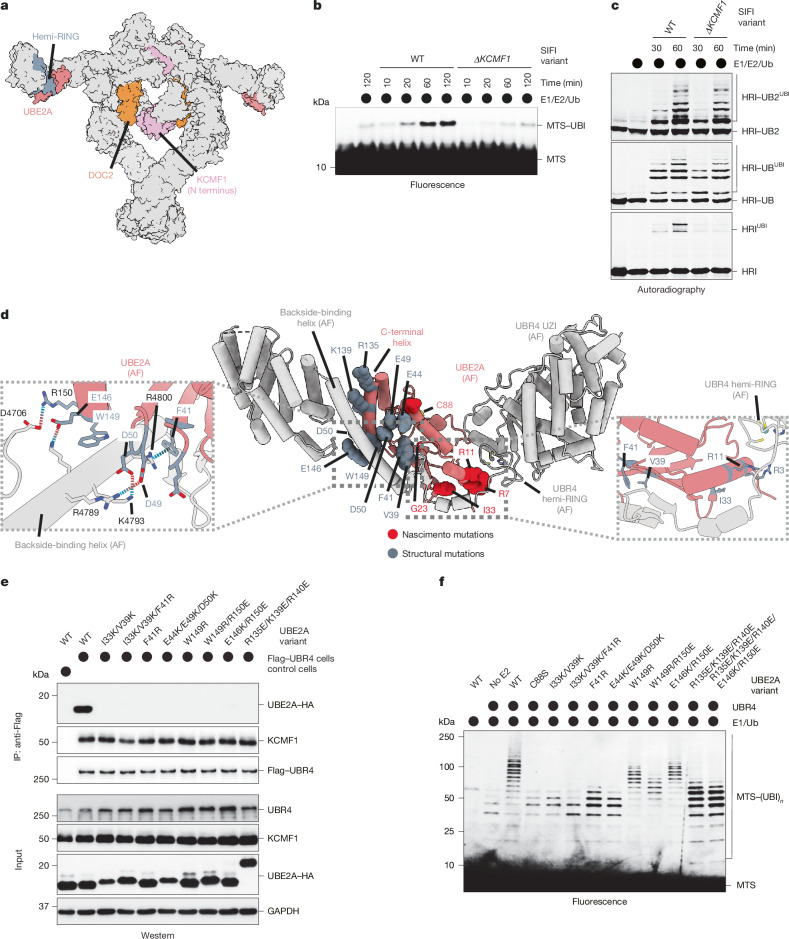
SIFI uses distinct modules for ubiquitin chain initiation and elongation. **a**, Surface representation of SIFI with DOC2 (orange), KCMF1^N138^ (plum), UBE2A (coral) and the hemi-RING domain (blue grey) highlighted. **b**, A time-course ubiquitylation assay of a fluorescently labelled presequence peptide by SIFI or SIFI(ΔKCMF1) indicates that KCMF1 is required for the transfer of the first ubiquitin. Reactions were performed in the presence of ubiquitin(K48R) to prevent chain elongation. Similar results were observed in *n* = 2 experiments. **c**, Fusion of one or two ubiquitin molecules to HRI^NT^ (HRI-UB; HRI-UB2) overcomes the defect of SIFI(ΔKCMF1) in catalysing ubiquitin chain formation. ^35^S-labelled HRI^NT^–SUMO (the SUMO tag was used for solubility) was incubated with WT SIFI or SIFI(ΔKCMF1) and ubiquitylation was followed by autoradiography. Similar results were observed in *n* = 2 experiments. **d**, AF2 model of UBE2A bound by the C-terminal region of UBR4 through three interfaces (middle). Mutations introduced to the interfaces are highlighted. Left, the interaction network between UBE2A and the backside binding helix of UBR4. Right, interactions near the N-terminal helix of UBE2A. **e**, Mutations in UBE2A that disrupt binding to the backside helix or the lock loop of UBR4, respectively, prevent stable integration of UBE2A into SIFI, as revealed by UBR4 affinity purification and western blotting using specific antibodies. The experiment was performed once and validated by in vitro ubiquitylation. **f**, UBE2A mutations impair MTS peptide ubiquitylation. Chain initiation occurred through an E2 that co-purified with SIFI. The experiment was performed once and validated with co-immunoprecipitation. Gel source data are provided in Supplementary Fig. 1.

**Extended Data Fig. 7:**
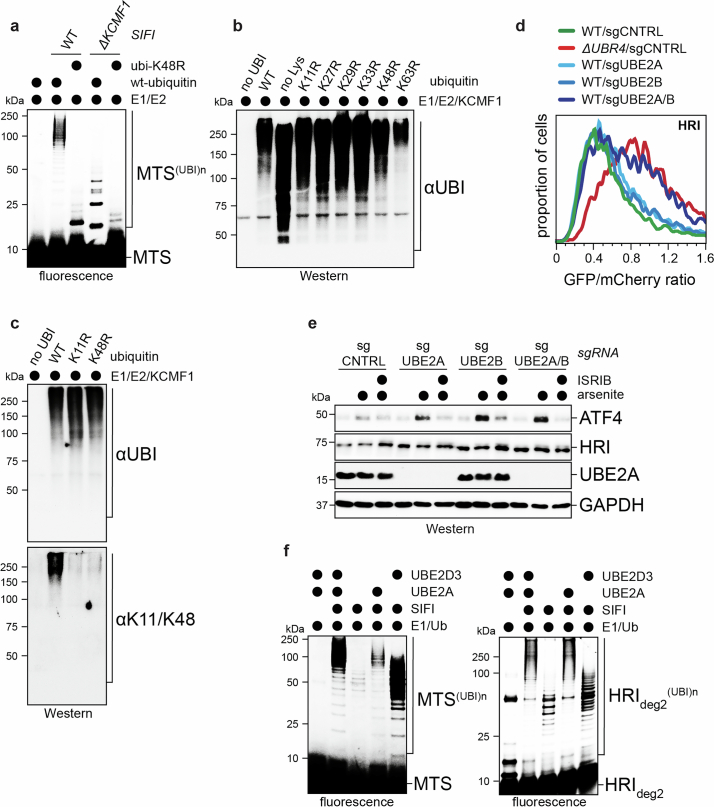
UBE2A promotes ubiquitin chain elongation by SIFI. **a**. KCMF1 does not determine linkage specificity of SIFI. SIFI was purified from WT or *ΔKCMF1* cells and tested for its ability to catalyse ubiquitylation of a mitochondrial presequence. As indicated, either wt-ubiquitin or ubiquitin-K48R were used. Experiment performed once. **b**. KCMF1 can modify ubiquitin residues independently of a specific Lys residue. KCMF1 was affinity-purified from *ΔUBR4* cells and incubated with E1, UBE2D3, and indicated ubiquitin variants. Ubiquitin polymers were detected by Western blotting. Similar results in n = 2 independent experiments. **c**. KCMF1 can introduce ubiquitin branches. KCMF1 was affinity-purified from *ΔUBR4* cells and incubated with E1, UBE2D3, and indicated ubiquitin variants. Reaction products were visualized with either ubiquitin antibodies or with bispecific K11/K48-antibodies that preferentially detect K11/K48-branched ubiquitin molecules^17^. Experiment performed once. **d**. UBE2A and its close homologue UBE2B are required for HRI degradation. UBE2A and UBE2B were co-depleted from 293 T cells, and HRI stability was monitored using a HRI-GFP::mCherry reporter, as described before. Similar results in n = 2 independent experiments. **e**. UBE2A and UBE2B co-depletion results in increased activation of the integrated stress response, as determined by αATF4-Western blotting. Experiment performed once. **f**. UBE2A promotes ubiquitin chain elongation. SIFI was affinity-purified via UBR4^FLAG^ and incubated with E1, ubiquitin and a mitochondrial presequence peptide (left) or HRI degron helix 2 peptide (right), resulting in short ubiquitin chains on the presequence substrate likely catalysed by an E2 that co-purified with SIFI. Addition of the E2 UBE2D3 results in an increase in short ubiquitin chains, indicative of a role of UBE2D3 in chain initiation. Addition of UBE2A leads to selective elongation of ubiquitin chains that had already been initiated. Similar results in n = 2 independent experiments. For gel source data, see Supplementary Fig. 1.

As substrates bind next to the N-terminal domains of KCMF1 (Fig. 4a), KCMF1 might help to transfer the first ubiquitin, a reaction that must accommodate the diverse sequence context of Lys residues in SIFI’s many targets. Loss of KCMF1 indeed impaired attachment of the first ubiquitin (Fig. 4b), which was mitigated when we bypassed chain initiation by fusing ubiquitin to a target (Fig. 4c). In cases in which initiation could occur, SIFI(ΔKCMF1) was able to build short ubiquitin chains that were connected through the correct Lys48 linkage (Extended Data Fig. 7a). KCMF1 therefore supports substrate modification but does not determine the linkage specificity of chain formation. Affinity purifications from *ΔUBR4* cells showed that KCMF1 can modify ubiquitin independently of a particular lysine (Extended Data Fig. 7b). When encountering an internal subunit of a growing chain, KCMF1 can therefore branch off new conjugates (Extended Data Fig. 7c), which is equivalent to initiating a new chain off an existing polymer^4,17^.

With KCMF1 supporting initiation, we hypothesized that chain elongation relies on the catalytic modules provided by UBR4^36^. UBR4 acts with only UBE2A or UBE2B^21^, and co-depletion of these E2 proteins stabilized HRI and increased stress signalling (Extended Data Fig. 7d,e). To test whether UBR4 drives chain elongation, we assessed the role of E2 enzymes in SIFI-dependent ubiquitylation, making use of the observation that SIFI co-purified with an E2 that could initiate some chains if supplied with E1 and ubiquitin. While addition of UBE2D3 increased the abundance of short chains, indicative of a role in initiation, supplementing SIFI with the UBR4-specific UBE2A resulted in selective chain extension (Extended Data Fig. 7f).

UBR4 recruits UBE2A through a three-sided embrace that to our knowledge has not been seen for other E3 ligases (Fig. 4d). While the hemi-RING of UBR4 binds to the N-terminal α-helix of UBE2A, as noted previously^21^, a long UBR4 helix and a short β-sheet together engage the backside of the E2. The helix connects to a UBR4 loop that contacts the C-terminal α-helix of UBE2A (Fig. 4d). Mutation of UBE2A residues at each interface prevented integration into SIFI and impaired chain elongation (Fig. 4e,f), showing that the E2 embrace is required for SIFI activity.

**Extended Data Fig. 8:**
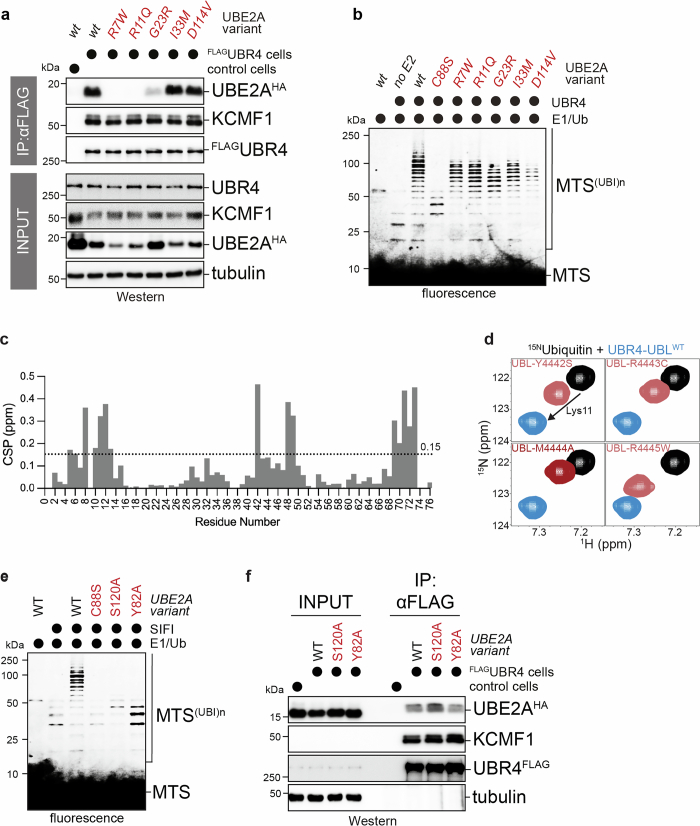
Structural insight into UBE2A binding and linkage specificity. **a**. UBE2A mutants detected in patients of Nascimento Syndrome are impaired in binding SIFI in cells, as determined by UBR4 affinity-purification and Western blotting. Similar results in n = 2 independent experiments. **b**. UBE2A variants in Nascimento Syndrome are impaired, but not fully inhibited, in catalysing ubiquitin chain elongation on SIFI substrates. D114 of UBE2A is at the interface with donor ubiquitin. Similar results in n = 2 independent experiments. **c**. Chemical shift perturbation analysis of ^15^N-ubiquitin with 2.5 molar equivalents of UBR4 UBL domain. **d**. NMR analysis of UBR4 UBL mutants binding to ^15^N-ubiquitin. Lys11 of ubiquitin was chosen as a reporter of binding because it is far away from the interface directly affected by UBL mutations. **e**. Y82 and S120 of UBE2A are essential for ubiquitin chain elongation by SIFI, as detected in an in vitro ubiquitylation assay as described above. Experiment performed once. **f**. Mutation of Y82 or S120 of UBE2A does not prevent binding of the E2 to SIFI. Endogenous SIFI was purified from UBR4^FLAG^ cells and co-purifying UBE2A^HA^ variants were detected by Western blotting. Experiment performed once. For gel source data, see Supplementary Fig. 1.

*UBE2A* mutations that cause the neurodevelopmental Nascimento syndrome map to each part of the E2 embrace^37–39^ (Fig. 4d): Arg7 of UBE2A faces the UBR4 hemi-RING; Arg11 binds to the loop that connects the hemi-RING to the backside helix; Gly23 is next to the backside β-sheet of UBR4; and the C-terminal helix of UBE2A, of which the deletion was first noted in patients with Nascimento syndrome^39^, engages the UBR4 loop. Mutants of Arg7, Arg11 or Gly23 were impaired in binding to SIFI and catalysing chain extension (Extended Data Fig. 8a,b), highlighting the physiological importance of the E2 embrace and implying that defective stress response silencing contributes to symptoms of patients with Nascimento syndrome.

## Ubiquitin handover

**Fig. 5:**
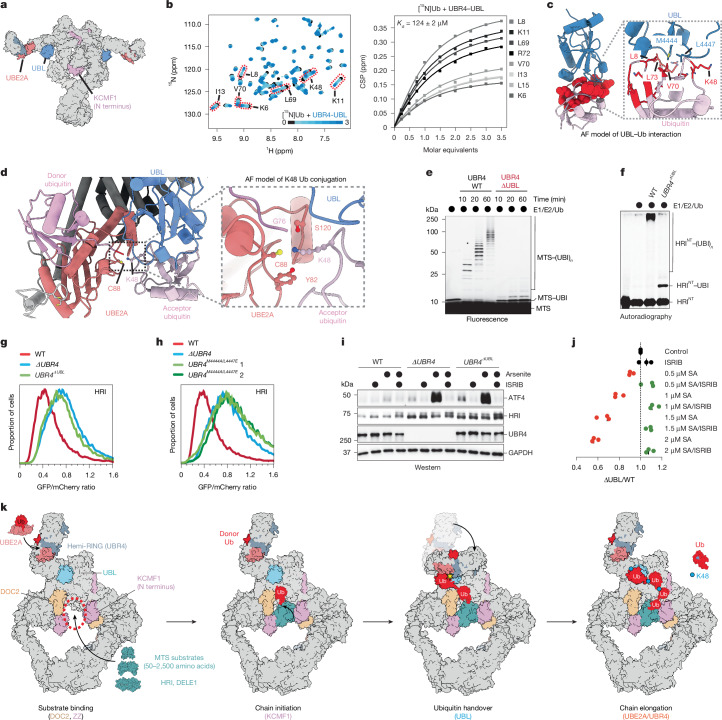
Ubiquitin handover from chain initiation to elongation modules. **a**, SIFI’s UBL domain (blue) is located in between KCMF1 (pink) and the UBR4–UBE2A chain elongation centre (coral/blue grey). **b**, Overlaid ^1^H-^15^N HSQC NMR spectra of ^15^N-labelled ubiquitin titrating unlabelled UBL domain (left). Right, ^1^H-^15^N NMR spectroscopy titration analysis of unlabelled UBL domain and ^15^N-labelled ubiquitin. CSP, chemical shift perturbations. **c**, Model of the UBL–ubiquitin interaction, highlighting interface residues affected in NMR binding experiments. **d**, AF3 model illustrating the C-terminal conformational change of UBR4 that brings UBE2A (coral) and donor Ub close to UBL (blue) and acceptor Ub (left). Right, magnified view that highlights Gly76 of donor Ub (light pink), Lys48 of acceptor Ub (dark pink), and Ser120 and Tyr82 of UBE2A. **e**, In vitro ubiquitylation of a fluorescently labelled MTS peptide shows that SIFI(ΔUBL) is deficient in ubiquitin chain elongation, but not in initiation. Similar results were observed in *n* = 2 experiments. **f**, In vitro ubiquitylation of ^35^S-labelled HRI^NT^–SUMO shows that the UBL domain is required for chain elongation. Similar results were observed in *n* = 3 experiments. **g**, HRI stability was analysed by flow cytometry using *HRI-GFP::mCherry*. Similar results were observed in *n* = 3 experiments. **h**, Endogenous mutation of key UBL residues stabilizes HRI to the same extent as complete *UBR4* inactivation. Similar results were observed in *n* = 2 experiments. **i**, WT or *UBR4*^*ΔUBL*^ cells were exposed to sodium arsenite, and ATF4 levels were monitored by western blotting. Similar results were observed in *n* = 2 experiments. **j**, Cell competition assay with sodium arsenite and ISRIB, performed as described above. *n* = 3 independent replicates shown with the median of these experiments. **k**, Model of SIFI-dependent substrate ubiquitylation. Substrates bind within the central cavity of SIFI. Ubiquitin chain initiation is mediated dependent on the ZZ-type domain of KCMF1, before a UBL domain hands substrate-attached ubiquitin over to UBE2A for Lys48-linkage specific chain elongation. Gel source data are provided in Supplementary Fig. 1.

As UBE2A lacks linkage specificity^21,40,41^, additional factors must encode SIFI’s preference for ubiquitin Lys48. In between initiation and elongation modules, SIFI contains a UBL domain (Fig. 5a). ^1^H-^15^N heteronuclear single quantum coherence (HSQC) nuclear magnetic resonance (NMR) spectroscopy showed that this domain binds to ubiquitin with a *K*_D_ of 124 μM, an affinity sufficient to capture substrate-attached ubiquitin while still enabling processive chain formation (Fig. 5b). This interaction centres on UBR4 Met4444, Leu4447 and Arg residues mutated in cancer cells, which engage a surface centred on Leu8 of ubiquitin (Fig. 5b,c and Extended Data Fig. 8c,d). Notably, AF models showed that the UBL domain orients ubiquitin so that a small movement of the peripheral SIFI arms, as implicated by the flexibility of the UBE2A module (Supplementary Video 4), is sufficient to present Lys48 to the active site of UBE2A (Fig. 5d). The ε-amino group of Lys48 in UBL-bound ubiquitin is guided towards the catalytic Cys88 of UBE2A by Tyr82 and Ser120 of the E2, which are essential for chain elongation without affecting SIFI-binding (Extended Data Fig. 8e,f). These results explain observations that Ser120 of UBE2A is required for substrate ubiquitylation^42^, and strongly suggest that the UBL domain hands substrate-attached ubiquitin over to UBE2A for Lys48-specific chain formation.

**Extended Data Fig. 9:**
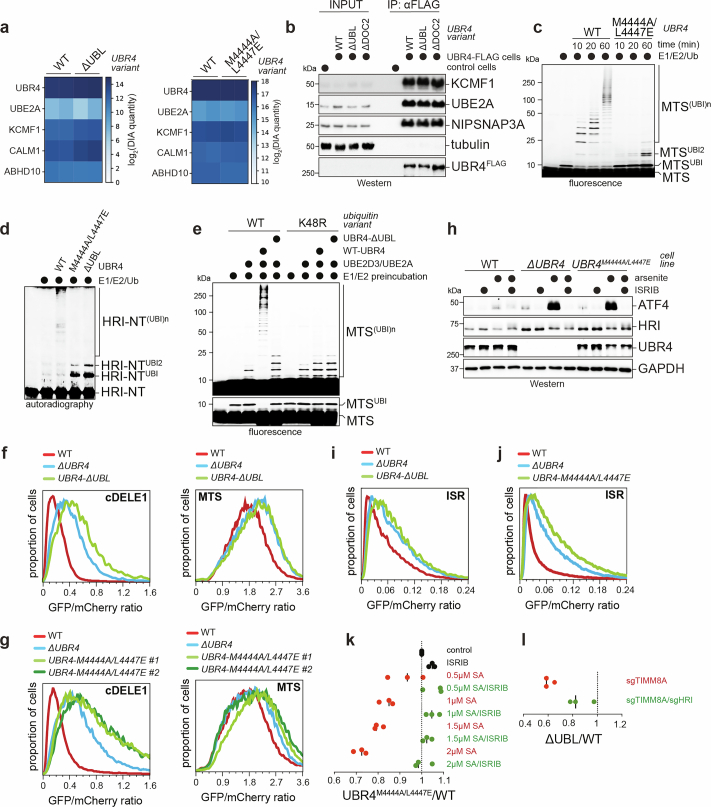
The UBL domain of UBR4 is essential for ubiquitin chain elongation. **a**. Mass spectrometry of SIFI^ΔUBL^ indicates that the UBL domain is not required for structural integrity of SIFI. Endogenous SIFI was affinity-purified from WT, *UBR4*^*ΔUBL*^ or *UBR4*^*M4444A/L4447E*^ cells and bound proteins were detected by mass spectrometry. Technical replicates are shown. **b**. Western blotting of SIFI^ΔUBL^ shows that the UBL domain is not required for structural integrity of SIFI. Endogenous SIFI was affinity-purified from WT or *UBR4*^*ΔUBL*^ cells and bound proteins were detected by Western blotting. Experiment performed once and validated by mass spectrometry. **c**. Mutation of the UBL domain in UBR4 prevents ubiquitin chain elongation, as seen with a TAMRA-labelled presequence peptide as substrate. Similar results in n = 2 independent experiments. **d**. Mutation or deletion of the UBL domain in UBR4 both prevent ubiquitin chain elongation by SIFI, as seen with ^35^S-labelled HRI^NT^ ~ SUMO as a substrate. **e**. Deletion of the UBL prevents ubiquitin chain elongation, even if initiation was performed independently of SIFI by a 2 h preincubation with E1/E2. Similar results in n = 2 independent experiments. **f**. The UBL domain of UBR4 is required for degradation of DELE1 or unimported mitochondrial proteins. Protein stability was determined by flow cytometry, as described before. Similar results in n = 2 independent experiments. **g**. Mutation of the UBL domain stabilizes cDELE1 and unimported mitochondrial proteins, as seen by flow cytometry with two independent clones expressing endogenous UBR4^M4444A/L4447E^. Similar results in n = 2 independent experiments. **h**. Mutation of the UBL domain in UBR4 results in increased stress signalling, as seen by an increase in ATF4 levels after import was compromised with arsenite. Experiment validated with two independent clones. **i**. Deletion of the UBL domain in UBR4 increases stress response activation upon sodium arsenite treatment, as detected by uORF-ATF4 reporters in flow cytometry as described above. Similar results in n = 2 independent experiments. **j**. Mutation of the UBL domain in UBR4 increases stress response activation, as measured by flow cytometry. Experiment validated with two independent clones. **k**. Mutation of the UBL domain in UBR4 compromises cell survival upon arsenite-induced import stress, as seen in a competition experiment with WT cells. n = 3 independent replicates shown with median of these experiments. **l**. The UBL domain of UBR4 is essential for cell survival upon mitochondrial import stress. WT and *UBR4*^ΔUBL^ cells were labelled with GFP and mCherry, respectively, mixed, and depleted of either TIMM8A or TIMM8A/HRI, as indicated. After 12 d of co-culture, the ratio between cell types was determined by flow cytometry. n = 3 independent replicates shown with median of these experiments.

We excised the entire UBL domain or mutated two residues at the interface with ubiquitin (Met4444/Leu4447) at all *UBR4* loci. Affinity purifications confirmed that SIFI remained intact (Extended Data Fig. 9a,b and Supplementary Table 1). Notably, SIFI complexes containing UBR4(M4444A/L4447E) or UBR4(ΔUBL) were strongly compromised in chain elongation, while transfer of the first ubiquitin was not affected (Fig. 5e,f and Extended Data Fig. 9c,d). Deletion of the UBL domain also prevented chain elongation if chain initiation had been accomplished before SIFI addition (Extended Data Fig. 9e). HRI, DELE1 and mitochondrial precursors were stabilized by UBL mutation or deletion to the same degree as in *ΔUBR4* cells (Fig. 5g,h and Extended Data Fig. 9f,g), leading to increased stress signalling (Fig. 5i and Extended Data Fig. 9h–j). Cells expressing SIFI without an intact UBL domain accordingly revealed a strong fitness defect when experiencing import stress, which was rescued by HRI loss or ISRIB (Fig. 5j and Extended Data Fig. 9k,l). We conclude that the UBL domain hands substrate-attached ubiquitin over to UBE2A for chain elongation. Coordination between flexible initiation and linkage-specific elongation modules allows SIFI to decorate many diverse proteins with a specific ubiquitin tag, a prerequisite for timely and efficient stress response silencing.

## Discussion

Silencing of the integrated stress response requires that a single enzyme, SIFI, can sense stress across cellular scales. SIFI indeed processes a large array of mislocalized or cleaved proteins that accumulate in the cytoplasm only during stress and competitively inhibit ubiquitylation of the stress response kinase HRI^4,14,17,20,32,43^. Our study shows that SIFI manages such diverse proteins by combining flexible substrate-modification modules with ubiquitin handover to sterically restricted elongation centres (Fig. 5k).

SIFI binds to its targets within an easily accessible scaffold that also contains modules required for ubiquitin chain initiation. These SIFI domains show high degrees of flexibility that suggest that that they can adapt to targets ranging in size from around 5 to 250 kDa. Even if substrates are recognized through distinct degrons, they occupy overlapping space on SIFI, which provides a rationale for their competition with HRI as required for timely stress response silencing.

Elongation of Lys48-linkage specific polymers occurs at separate sites in the peripheral SIFI arms. Contrasting the flexibility of its initiation modules, SIFI’s elongation centres are built around an E2, UBE2A, that is captured by SIFI on three sides. UBE2A residues at each interface are required for chain extension and mutated in Nascimento syndrome, showing that the E2 embrace is critical for ubiquitylation. The elongation module is therefore sterically restricted. Given that E1 and E3 bind to overlapping sites on E2s^44,45^, it will be interesting to see how UBE2A is recharged with ubiquitin for efficient chain extension.

Although it was known that ubiquitin chain initiation and elongation can be executed by distinct enzymes^46,47^, SIFI illustrates how these activities are coordinated with each other by ubiquitin handover through a UBL domain that is tethered to the scaffold by long linkers. The UBL domain orients substrate-attached ubiquitin towards UBE2A so that only Lys48 is available for chain extension, a feature that requires some movement of the SIFI arms around a hinge close to the UBL domain. While distinct from previously described mechanisms of chain elongation^48,49^, ubiquitin handover is an efficient solution for enzymes that need to modify many substrates with a specific ubiquitin tag. Ubiquitin handover might therefore be a general feature of machines that ubiquitylate diverse targets and, indeed, ubiquitin-binding domains are often found in quality-control E3 ligases^50–54^.

Its ability to decorate conformationally diverse proteins with a linkage-specific ubiquitin tag suggests that SIFI should be harnessed for the emerging modality of targeted protein degradation. Our structures point to the ZZ, DOC2 and UBR domains of SIFI as potential binding sites for compounds. As SIFI already counteracts protein aggregation and is highly expressed in the brain^17,55^, it should be particularly tested for applications against neurodegenerative diseases. Our work therefore not only reveals the molecular basis of SIFI activity in the integrated stress response, but also points to strategies to unlock targeted protein degradation to disease areas of high unmet need.

## Methods

### Data reporting

No statistical methods were used to predetermine sample size. The experiments were not randomized and the investigators were not blinded to allocation during experiments and outcome assessment.

### Mammalian cell culture

HEK293T cells were maintained in DMEM + GlutaMax (Gibco, 10566-016) plus 10% fetal bovine serum (VWR, 89510-186). HEK293T cells that were adapted to growth in suspension were cultured in FreeStyle 293 Expression medium (Gibco, 12338026) with 1% FBS. Expi293F cells used for transient transfection for protein purification were grown in Expi293 Expression medium (A1435101, Gibco). All cell lines were purchased directly from the UC Berkeley Cell Culture Facility, authenticated by short-tandem-repeat analysis before freezing stocks (9 February 2022) and were routinely tested for mycoplasma contamination using the Mycoplasma PCR Detection Kit (abmGood, G238). All of the cell lines tested negative for mycoplasma.

Plasmid transfections were performed using polyethylenimine (PEI, Polysciences 239661) at a 1:6 ratio of DNA (in μg) to PEI (in μl at a 1 mg ml^−1^ stock concentration), Lipofectamine 3000 transfection reagent (Thermo Fisher Scientific, L3000008) or Expifectamine according to the manufacturer’s instructions or ExpiFectamine 293 (Thermo Fisher Scientific, A14524). Lentiviruses were produced in HEK293T cells by co-transfection of lentiviral and packaging plasmids using PEI. Virus-containing supernatants were collected 48 h and 72 h after transfection, and the supernatants were spun down, aliquoted and stored at −80 °C for later use. For lentiviral transduction, 10^5^ cells were seeded into 24-well plates and centrifuged for 45 min at 1,000*g* after addition of lentiviral particles and 6 μg ml^−1^ polybrene (Sigma-Aldrich, TR-1003). HEK293T transduced cells were drug-selected 24 h after infection with the following drug concentrations when applicable: puromycin (1 μg ml^−1^, Sigma-Aldrich, P8833), blasticidin (7.5 μg ml^−1^, Thermo Fisher Scientific, A1113903).

### Plasmids

The list of all constructs used in this study is provided in Supplementary Table 2. Most cloning was performed using Gibson assembly with the HIFI DNA Assembly master mix (NEB, E2621L).

### Generation of CRISPR–Cas9 genome-edited cell lines

All CRISPR–Cas9 edited cell lines used in this study were generated from HEK293T cells. sgRNA sequences were designed using the online resource provided by IDT. DNA oligonucleotides for sgRNA and their complementary sequence were phosphorylated (NEB, M0201), annealed and ligated (NEB, M0202) into pX330 (Addgene, 42230). HEK293T cells were cultured in a six-well plate and transfected at 50% confluence with 2 μg of px330 plasmids (and 1 μl of 10 μM single stranded donor oligo when applicable) using Mirus TransIT-293 Transfection reagent (Mirus, MIR2705). At 48 h after transfection, individual clones were expanded in 96-well plates. Homozygous clones were screened by PCR and DNA sequencing and confirmed by western blotting when applicable.

HEK293T Flag–UBR4, ΔUBR4, Flag–UBR4 ΔKCMF1 cells were generated previously^4^. For the generation of N-terminally Twin-Strep-tagged UBR4 cells (Twin-Strep–UBR4), we used the following sgRNA: 5′-GCGGAAGATGGCGACGAGCGG-3′ and ssODN 5′-CCGGTGGCAAGCCCCCGGAGGGAGCCGCAGTAGTACGACGGAAGATGAGCGCCTGGAGTCACCCTCAGTTTGAGAAAGGCGGAGGTAGCGGAGGTGGCTCTGGCGGAAGCGCCTGGTCACACCCACAGTTCGAGAAGGGCGGAGGTAGCGCGACGAGCGGCGGCGAAGAGGCGGCGGCAGCGGCTCCGGCGCCGGG-3′.

UBR4(ΔUBL) (Δ4342–4434), UBR4(ΔDOC2) (Δ3538–3721) were generated in the Flag–UBR4 background, with the following protospacer sequences that created in-frame deletions: UBR4(ΔUBL): 5′-CCTTGTTCCATGGACACTCG-3′ and 5′-CATTAGTCAGATGCCTCCAA-3′; UBR4(ΔDOC2): 5′-CGGGTTATTACACACCAGGC-3′ and 5′-ATGGGATCCACTGCACAGCA-3′ and the following sODN to repair: 5′-CATGCTAAGACTGGTTTCTTCCTTAGCACTTTGTCTGGCTTAGTGGAGTTTGATGGCTATTACCTGGAGAGCGATCCCTGCCTCGTGTGTAATAACGGCAGCAGCGCAGTGGATCCTATTGAGAATGAAGAAGACCGGAAGAAGGTGAGGCCAGATCTGGCCTAGACTCAGGGCTGTGGCCTTGATCTGGACTTTGGGCA-3′.

UBR4(M4444A/L4447E) cells were generated by nucleofecting Cas9–RNP complexes (sgRNA: 5′-GGAUUGUUUAUCGUGCCCGG-3′) and the ssODN template (5′-AGTCCAGGGACTCAATGAACTCCTCTGTGGCATCGCCCAGCTCCCCCCGGGCACGATAAACAATCCTCATGGGCTCTCCCTGAGCAGAGAAA-3′) into Flag–UBR4 cells using the Lonza 4D-Nucleofector X Unit (program CM130) and the SF Cell Line 4D-Nucleofector X Kit S. For each reaction, 2.5 µl of recombinant Cas9 (40 µM) was incubated with 1.3 µl of sgRNA (100 µM) and incubated for 15 min at room temperature with 4.7 µl of SF solution, after which 1.5 µl of ssODN (100 µM) and 2 × 10^5^ cells in 10 µl of SF solution were added to the RNP complex. Immediately after nucleofection, cells were plated into 12-well plates. The editing efficiency was increased by adding Alt-R HDR Enhancer V2 from IDT for 16 h. Then, at 72 h after nucleofection, the bulk editing efficiency was determined by PCR and DNA sequencing and individual clones were expanded in 96-well plates. Homozygous clones were screened and confirmed multiple times.

### Protein expression and purification

To purify the endogenous SIFI complex, HEK293T cells with the *UBR4* gene endogenously tagged with a Flag or Strep tag were collected from 150 mm culture dishes or adapted suspension cells. To purify SIFI complex through affinity-tagged KCMF1, Twin-Strep-II-tagged KCMF1 was transiently expressed in Expi293F cells for 48 h before collection. Cells were lysed in cell lysis buffer containing 40 mM HEPES pH 7.5, 150 mM NaCl, 1 mM DTT, 0.1% Nonidet P-40 (NP-40), benzonase nuclease (Millipore-Sigma, 70746-4), proteasome inhibitor carfilzomib and Roche cOmplete protease inhibitor cocktail (Sigma-Aldrich, 11836145001). Cells were lysed for 20 min and homogenized using a Dounce homogenizer. Cell lysates were cleared by centrifugation at 4,000*g* for 10 min followed by centrifuging at 36,000*g* for 40 min. The supernatant solution was subsequently collected and incubated with M2 Flag resin (Sigma-Aldrich, A2220) or Strep-Tactin XT 4Flow resin (IBA Lifesciences, 16674714) for 4–6 h at 4 °C. Affinity resin was washed thoroughly with wash buffer (150 mM HEPES pH 7.5, 150 mM NaCl, 1 mM DTT), then eluted with elution buffer (500 μg ml^−1^ 3×Flag peptide (Sigma-Aldrich, F4799) for M2 Flag resin, 50 mM biotin (IBA Lifesciences, 21016005) for Strep-Tactin XT 4Flow resin). Eluted proteins further purified using the Superose 6 10/300 size-exclusion chromatography (SEC) column (Cytiva, 17517201).

### Cryo-EM sample preparation

Purified SIFI complex, SIFI–substrate mixture or cross-linked SIFI-substrate mixture samples were concentrated to 2–4 mg ml^−1^ concentration for cryo-preservation. The concentrated samples were mixed with a final concentration of 0.02% (w/v) fluorinated octylmaltoside (Anatrace) immediately before cryo-freezing to prevent protein denaturation at the air–water interface. Then, 2.6 μl of the sample was applied to a glow-discharged 300-mesh Quantifoil R1.2/1.3 grid and incubated for 15 s before being blotted and plunge-vitrified in liquid ethane, which was cool-protected by liquid nitrogen. Grid freezing was performed using a Mark IV Vitrobot (Thermo Fisher Scientific) system operating at 12 °C and 100% humidity.

### Cryo-EM data collection and processing

Cryo-EM data were collected using the 300 kV Titan Krios G3i or G2 electron microscope (Thermo Fisher Scientific) equipped with a BIO Quantum energy filter (slit width 20 eV). Data were collected using SerialEM software^56^ at a nominal ×105,000 magnification with a pixel size of 1.05 Å per pixel (G3i) or 0.83 Å per pixel (G2). Videos were recorded using a 6k x 4k Gatan K3 Direct Electron Detector operating in super-resolution CDS mode. Each video was composed of 40 subframes with a total dose of 60 e^−^ Å^−2^, resulting in a dose rate of 1.5 e^−^ Å^−2^. A total of more than 6,000 videos was recorded for each sample. Data processing, including motion correction, CTF estimation, particle picking, 2D class averaging and 3D refinement, was performed using cryoSPARC v.4.3 workflow^57^. All videos were 2× binned and patch motion-corrected. After particle picking and several iterations of 2D class averaging, the initial 3D volume was calculated using ab initio 3D reconstruction. Selected particles were then used for non-uniform 3D refinements^58^. Protein motion and flexibility was calculated using cryoSPARC 3D Flexible Refinement^59^ (3DFlex).

### Model building and structural analysis

Coordinates for the N- and C-terminal halves of SIFI complex were built separately into their respective cryo-EM maps. Coordinates for regions of the EM maps with higher local resolutions, such as the UBR4 α-helical armadillo repeats, were manually built into the map, whereas, for regions with lower local resolutions (~5–8 Å), AF2^60^ models were used and protein secondary structures, including α-helices and β-strands, were fitted into the EM density. For regions with very low resolutions (<8 Å), such as the UBR4 WD40 domain, UBR, DOC1, N-terminal domain of KCMF1, UBL domain and hemi-RING/UBE2A, AF2 models were fitted into the low-resolution density in ChimeraX. Minimal adjustments were done at the secondary-structure level. Helices were adjusted to fit into the map, and amino acid side chains were refined to their preferred rotamers and clashes were minimized. Certain low-resolution models, such as the UBL domain of UBR4, the UBE2A-binding domains and the N-terminal domain of KCMF1, were validated through other biochemical assays such as NMR, binding assays and in vitro ubiquitylation assays. Coordinates of the medium-resolution to high-resolution regions were refined with multiple iterations of PHENIX real-space refinement^61^ and manual refinement in Coot^62^. Buried surface area of the protein–protein interface was calculated using PDBePISA^63^ with a 1.4 Å probe. Potential hydrogen bonds were assigned using the geometry criteria of <3.5 Å separation distance and >90° acceptor–donor–hydrogen (A-D-H) angle. A maximum distance of 4.0 Å was allowed for a potential van der Waals interaction. Structures were further analysed, and structural presentations were prepared using PyMOL v.2 (Schrödinger) and ChimeraX^64,65^.

### MS analysis

MS was performed on immunoprecipitates prepared from forty 15-cm plates of endogenously *Flag-UBR4* and *Flag-UBR4 ΔKCMF1* HEK293T cell lines or twenty 15-cm plates of WT or *ΔUBR4* HEK293T cells transfected with *KCMF1-3×Flag* DNA. Cells were lysed in lysis buffer (40 mM HEPES, pH 7.5, 150 mM NaCl, 0.2% NP-40, benzonase (Sigma-Aldrich, E1014) and 1× cOmplete protease inhibitor cocktail (Roche, 11836170001) and 1× PMSF), lysed extracts were clarified by centrifugation at 21,000*g* and bound to anti-Flag M2 affinity resin (Sigma-Aldrich, A2220) for 2 h with rotation at 4 °C. Immunoprecipitates were then washed four times and eluted three times at 30 °C with 0.5 mg ml^−1^ of 3×Flag peptide (Sigma-Aldrich, F4799) buffered in 1× PBS plus 0.1% Triton X-100. Elutions were pooled and precipitated overnight at 4 °C with 20% trichloroacetic acid. Centrifuged pellets were washed three times with an ice-cold acetone/0.1 N HCl solution, dried, resolubilized in 8 M urea buffered in 100 mM Tris pH 8.5, reduced with TCEP, at a final concentration of 5 mM, (Sigma-Aldrich, C4706) for 20 min, alkylated with iodoacetamide, at a final concentration of 10 mM (Thermo Fisher Scientific, A39271) for 15 min, diluted fourfold with 100 mM Tris-HCl pH 8.5 and digested with 0.5 mg ml^−1^ trypsin (Promega, v5111) supplemented with CaCl_2_ (at a final concentration of 1 mM) overnight at 37 °C. Trypsin-digested samples were submitted to the UC Berkeley Vincent J. Coates Proteomics/Mass Spectrometry Laboratory for analysis. Peptides were processed using multidimensional protein identification technology and run on the LTQ XL linear ion-trap mass spectrometer. To identify high-confidence interactors, CompPASS analysis was performed against MS results from unrelated Flag immunoprecipitates performed in our laboratory. KCMF1, ABHD10 and NIPSNAP3A were the only three proteins with more than 10 spectral counts and showing a tenfold or more reduction in Flag–UBR4(ΔKCMF1) compared with Flag–UBR4.

Flag–UBR4, Flag–UBR4(ΔUBL) and UBR4(M4444A/L4447E) MS experiments (Extended Data Fig. 9a,b) were performed from twenty 15-cm plates lysed in lysis buffer (40 mM HEPES, pH 7.5, 150 mM NaCl, 0.2% NP-40, benzonase (Sigma-Aldrich, E1014) and 1× cOmplete protease inhibitor cocktail (Roche, 11836170001), 1× PMSF), clarified by centrifugation at 21,000*g*, and bound to anti-Flag M2 affinity resin (Sigma-Aldrich, A2220) for 2 h with rotation at 4 °C. Immunoprecipitates were then washed four times with (40 mM HEPES, pH 7.5, 150 mM NaCl, 0.2% NP-40) followed by three washes in PBS, all of the remaining liquid was removed with a crushed gel tip and the beads were flash-frozen in liquid nitrogen.

To monitor mitochondrial proteins bound to SIFI under mitochondrial import stress, fifteen 15-cm plates of subconfluent cells per condition were treated with 10 µM arsenite for 16 h. Cells were co-treated with 200 nM ISRIB to eliminate differences in ISR-activation-induced translational changes. To reduce SIFI occupancy with ABHD10, NIPSNAP3A and other mitochondrial interactors present after lysing mitochondria, cells were collected without freezing cell pellets and lysed in digitonin lysis buffer (40 mM HEPES 7.5, 150 mM NaCl, 10 μg ml^−1^ digitonin (GoldBio, D-180-2.5), with Roche cOmplete Protease Inhibitor Cocktail (Sigma-Aldrich, 11873580001), carfilzomib (2 μM, Selleckchem, S2853) for 10 min with rotation at 4 °C. Lysed cells were spun down at 2,000*g* for 5 min at 4 °C and the supernatant was collected. The supernatant was added to equilibrated anti-Flag-M2 Affinity Agarose Gel slurry (Sigma-Aldrich, A2220) and rotated for 2 h at 4 °C. The following wash and elution steps were the same as described above for NP-40 lysis.

Further sample processing was performed at the UC San Diego Proteomics Facility, where the protein samples were diluted in TNE (50 mM Tris pH 8.0, 100 mM NaCl, 1 mM EDTA) buffer. RapiGest SF reagent (Waters) was added to the mix to a final concentration of 0.1%, and the samples were boiled for 5 min. TCEP was added to 1 mM (final concentration) and the samples were incubated at 37 °C for 30 min. Subsequently, the samples were carboxymethylated with 0.5 mg ml^−1^ of iodoacetamide for 30 min at 37 °C followed by neutralization with 2 mM TCEP (final concentration). The proteins samples were then digested with trypsin (trypsin:protein ratio, 1:50) overnight at 37 °C. RapiGest was degraded and removed by treating the samples with 250 mM HCl at 37 °C for 1 h followed by centrifugation at 14,000 rpm for 30 min at 4 °C. The soluble fraction was then added to a new tube and the peptides were extracted and desalted using C18 desalting columns (Thermo Fisher Scientific, PI-87782). Peptides were quantified using BCA assay and a total of 1 μg of peptides were injected for LC–MS analysis. Trypsin-digested peptides were analysed by ultra-high-pressure liquid chromatography (UPLC) coupled with tandem mass spectroscopy (LC–MS/MS) using nano-spray ionization. The nanospray ionization experiments were performed using a TimsTOF 2 pro hybrid mass spectrometer (Bruker) interfaced with nanoscale reversed-phase UPLC (EVOSEP ONE). The Evosep method of 30 samples per day was performed using a 10 cm × 150 μm reversed-phase column packed with 1.5 μm C18-beads (PepSep, Bruker) at 58 °C. The analytical columns were connected with a fused silica ID emitter (10 μm inner diameter, Bruker Daltonics) inside a nanoelectrospray ion source (captive spray source, Bruker). The mobile phases comprised 0.1% formic acid as solution A and 0.1% formic acid/99.9% acetonitrile as solution B. The MS settings for the TimsTOF Pro 2 were as follows: the dia-PASEF method for proteomics. The values for mobility-dependent collision energy were set to 10 eV. No merging of TIMS scans was performed. The ion mobility (IM) was set between 0.85 (1/k0) and 1.3 (1/k0) with a ramp time of 100 ms. Each method includes one IM window per dia-PASEF scan with variable isolation window at 20 amu segments; 34 PASEF MS/MS scans were triggered per cycle (1.38 s) with a maximum of 7 precursors per mobilogram. Precursor ions in an *m*/*z* range of between 100 and 1,700 with charge states ≥3+ and ≤8+ were selected for fragmentation. Protein identification and label-free quantification were performed using Spectronaut 18.0 (Biognosys).

The values obtained with DIA analysis for the Flag–UBR4, Flag–UBR4(ΔUBL) and UBR4(M4444A/L4447E) values are represented for a subset of interactors that were found significantly enriched over the HEK293T background control samples and were previously validated.

For analysis of mitochondrial proteins bound to SIFI under mitochondrial import stress, after DIA analysis, contaminant proteins were excluded from the analysis if they appeared in ≥25% of datasets in the CRAPOME database^66^. Mitochondrial proteins were defined as such if annotated in MitoCarta3.0^67^. Identified peptides of mitochondrial proteins were run through the MTS prediction tool iMLP: iMTS-L predictor service^68^ and classified as a potential (i)MTS if there was a clear predicted iMTS-L propensity profile with a score >1. Detected intensities were normalized to bait (UBR4) and log_2_-transformed fold change values relative to the average DIA quantity of untreated WT samples are displayed as a heat map for all of the mitochondrial proteins and the top 19 abundant non-mitochondrial proteins. Gene-Ontology-term enrichment analysis was performed using Spectronaut 18.0 (Biognosys).

### Protein cross-linking

Cross-linking reactions were performed in 40 mM HEPES, pH 7.4, 150 mM NaCl, 1 mM DTT buffer containing 3 mg ml^−1^ total protein. BS3 (bis(sulfosuccinimidyl)suberate) (Thermo Fisher Scientific, A39266) dissolved in water was added to a final concentration of 0.5 mM to initiate cross-linking. Cross-linking was performed at room temperature for 30 min before quenching by addition of 100 mM final concentration of ammonium bicarbonate. Cross-linked samples were reduced with 10 mM final concentration of TCEP for 1 h at 37 °C, alkylated with 15 mM final concentration of iodoacetamide at room temperature in the dark for 30 min followed by addition of 15 mM final concentration of DTT. Sequencing-grade modified trypsin (Promega, V5111) was added at an enzyme:substrate ratio of 1:15. Tryptic digestion was performed for 6 h at 37 °C before acidification by addition of HCl to a final concentration of 250 mM. Digested samples were centrifuged in a microfuge at 21,000*g* for 10 min, and the resulting supernatant was transferred to an autosampler vial and stored at −80 °C before analysis using LC–MS/MS.

### MS analysis of cross-linked samples

MS and data analysis were based on previously described methods^69^. Sample digest (5 µl) was loaded onto a PepMap Neo Trap Cartridge (Thermo Fisher Scientific, 174500). The trap was brought online with a Bruker PepSep C18 15 cm × 150 µm, 1.9 µm column (Thermo Fisher Scientific, 1893471) connected to a 5 cm × 20 µm inner diameter Sharp Singularity Fossil Ion Tech tapered tip mounted in a custom constructed microspray source. Peptides were eluted from the column at 0.8 µl min^−1^ using a 90 min acetonitrile gradient. An Orbitrap Exploris 480 (Thermo Fisher Scientific) was used to perform MS in data-dependent acquisition (DDA) mode using the following parameters. Three methods were used, differing only by MS/MS resolution. The resolution at *m*/*z* 200 for MS was 60,000. MS/MS resolution was 15,000, 30,000 or 60,000. Cycle time was 2 s between MS scans. MS scan range was *m*/*z* 400–1,600. The automatic gain control was set to standard for MS and MS/MS, and the maximum injection times were set to auto. MS/MS spectra were acquired using an isolation width of 2 *m*/*z* and a normalized collision energy of 27. MS/MS acquisitions included +3 to +6 precursor ions and undefined precursor charge states were excluded. Dynamic exclusion was set at 10 s. All spectra were collected in centroid mode.

Acquired spectra were converted to mzML format using ProteoWizard’s msConvert (v.3.0.22335)^70^. Cross-links were identified from DDA data using the Kojak (v.2.0.3)^71^ search algorithm with post-processing using Percolator (v.2.08)^72^ and cross-link data visualization using the ProXL web application^73^. All data were filtered at a false-discovery rate (FDR) of 5% at the peptide level unless otherwise stated.

### Growth competition assays

HEK293T, *ΔUBR4*, *ΔDOC2*, *UBR4(M4444A/L4447E)* and *ΔUBL* cells were transduced to express either GFP or mCherry, respectively using the lentiviral pLVX-GFP-P2A-Blasticidin or pLVX-mCherry-P2A-blasticidin vector as described previously^4^.

For drug competition assays, 5 × 10^4^ WT GFP and 5 × 10^4^ *ΔUBR4* mCherry or UBR4 domain mutants were mixed in six-well plates. The next day, indicated concentrations of sodium arsenite (Ricca Chemical, 714216), were added for 72 h. The ratio of mCherry^+^/GFP^+^ cells was determined on the BD LSRFortessa instrument, analysed using FlowJo v.10.8.1 and normalized to the untreated sample. Gating strategies for flow cytometry analysis are shown in Supplementary Fig. 2.

### Drug treatments

For 3 day competition experiments with drug-treated cells, we used the following drug concentrations: 0.5–2 μM sodium arsenite (Ricca Chemical, 714216). For overnight drug treatments we used 5 μM sodium arsenite unless otherwise indicated in the figure legends. To inhibit the proteasome, we used 2 μM carfilzomib (Selleck Chemicals, S2853) for 6 h. ISRIB (Sigma-Aldrich, SML0843) was used at a concentration of 200 nM.

### Protein-stability reporter assay

The pCS2+-degron-GFP-IRES-mCherry reporter constructs (ISR, HRI and DELE1) were gene-rated as described previously^4^ and are listed Supplementary Table 2. Protein stability reporter assays were performed as described previously^4^. Cells were analysed on either the BD Bioscience LSR Fortessa or LSR Fortessa X20 system and the GFP/mCherry ratio was analysed using FlowJo. Gating strategies for flow cytometry analysis are shown in Supplementary Fig. 2.

### Western blotting

For western blot analysis of whole-cell lysates, cells were collected at the indicated timepoints by washing in PBS, pelleting and snap-freezing. Cells were lysed in lysis buffer (150 mM NaCl, 50 mM HEPES pH 7.5, 1% NP-40 substitute) supplemented with Roche cOmplete Protease Inhibitor Cocktail (Sigma-Aldrich, 11836145001), PhosSTOP Phosphatase Inhibitor Cocktail (Roche, 4906837001), carfilzomib (2 μM) and benzonase (EMD Millipore, 70746-4) on ice. The samples were then normalized to protein concentration using the Pierce 660 nm Protein Assay Reagent (Thermo Fisher Scientific, 22660). Then, 2× urea sample buffer (120 mM Tris, pH 6.8, 4% SDS, 4 M urea, 20% glycerol, bromophenol blue) was added to the samples. SDS–PAGE and immunoblotting was performed using the indicated antibodies. Images were captured on the ProteinSimple FluorChem M device.

### Small-scale immunoprecipitations

Cells were collected after washing in PBS, pelleted and snap-frozen. Frozen pellets were resuspended in lysis buffer (40 mM HEPES 7.5, 150 mM NaCl, 0.1% NP-40, with Roche cOmplete Protease Inhibitor Cocktail (Sigma-Aldrich, 11873580001), carfilzomib (2 μM, Selleckchem, S2853) and benzonase (EMD Millipore, 70746-4)). The lysates were incubated for 30 min on ice and cleared by centrifugation for 20 min at 21,000*g* at 4 °C. The supernatants were normalized to volume and protein concentration. Then, 5% of the sample was removed as an input and the sample was added to equilibrated anti-Flag-M2 Affinity Agarose Gel slurry (Sigma-Aldrich, A2220) and rotated for 1–2 h at 4 °C. The beads were washed three times in wash buffer (40 mM HEPES pH 7.5, 150 mM NaCl, 0.1% NP-40) and eluted with 2× urea sample buffer. SDS–PAGE and immunoblotting was performed using the indicated antibodies. Images were captured on a ProteinSimple FluorChem M device.

For immunoprecipitations performed from cells lysed in digitonin lysis buffer, cells were collected after washing in PBS and immediately lysed (never frozen) in digitonin lysis buffer (40 mM HEPES pH 7.5, 150 mM NaCl, 50 μg ml^−1^ digitonin (Sigma-Aldrich, D141)), with Roche cOmplete Protease Inhibitor Cocktail (Sigma-Aldrich, 11873580001), carfilzomib (2 μM, Selleckchem, S2853) for 10 min with rotation at 4 °C. Lysed cells were then spun down at 2,000*g* for 5 min at 4 °C and the supernatant was collected, and 5% input was removed. The supernatant was added to equilibrated anti-Flag-M2 Affinity Agarose Gel slurry (Sigma-Aldrich, A2220) and rotated for 1–2 h at 4 °C. The following wash and elution steps were the same as described above for NP-40 lysis. The pellet obtained after centrifugation of the digitonin-lysed cells was also subsequently lysed in NP-40 lysis buffer as described above to break open mitochondria and the sample was collected after centrifugation at 21,000*g* as the NP-40 fraction.

### Antibodies

The following antibodies were used for immunoblot analyses: anti-Flag (mouse, clone M2, Sigma-Aldrich, F1804, 1:1,000), anti-Flag (rabbit, Cell Signaling Technology (CST), 2368, 1:1,000), anti-HA-tag (rabbit, C29F4, CST, 3724, 1:1,000), anti-Strep (strepMAB-Classic, 2-1507-001, Iba Lifesciences, 1:10,000), anti-GAPDH (rabbit, D16H11, CST, 5174, 1:1,000), anti-HSP90β (rabbit, D3F2, CST, 7411, 1:1,000), anti-α-tubulin (mouse, DM1A, Calbiochem, CP06, 1:1,000), anti-UBR4/p600 (rabbit, A302, Bethyl, A302-279A, 1:1,000), anti-UBE2A/B (mouse, G-9, Santa Cruz, sc-365507, 1:150), anti-ATF4 (rabbit, D4B8, CST, 11815S, 1:1,000), anti-EIF2AK1 (rabbit, Proteintech, 20499-1-AP, 1:1,000), anti-KCMF1 (rabbit, Sigma-Aldrich, HPA030383, 1:1,000), anti-NIPSNAP3A (rabbit, Thermo Fisher Scientific, PA5-20657, 1:1,000), anti-ubiquitin (rabbit, CST, 43124, 1:1,000), anti-ABHD10 (rabbit, Thermo Fisher Scientific, PA5-103553, 1:1,000), goat anti-rabbit IgG (H+L) HRP (Vector Laboratories, PI-1000, 1:5,000), Sheep anti-mouse IgG (H+L) HRP (Sigma-Aldrich, A5906, 1:5,000), goat anti-mouse IgG-light-chain-specific HRP conjugated (Jackson Immunoresearch, 115-035-174, 1:5,000).

### In vitro transcription/translation of substrates

In vitro synthesized substrates were all cloned into pCS2 vectors containing a SP6 promoter (Supplementary Table 2) and generated using Wheat Germ Extract (Promega, L3260) as previously described^4^.

### In vitro ubiquitylation assays

For in vitro ubiquitylations, human SIFI complex was purified using an endogenous *Flag-UBR4* HEK293T cell line. Each in vitro ubiquitylation reaction required material from 2.5 15-cm plates of *Flag-UBR4*, *Flag-UBR4(ΔUBL)*, *Flag-UBR4(M4444A/L4447E)* or *Flag-UBR4(ΔDOC2)* cells. For *Flag-UBR4(ΔKCMF1)* cells, we used ten 15-cm plates per reaction. Frozen cell pellets were lysed at 4 °C for 30 min in 1 ml of lysis buffer per ten 15-cm plates (40 mM HEPES, pH 7.5, 5 mM KCl, 150 mM NaCl, 0.1% NP-40, 1 mM DTT, 1× cOmplete protease inhibitor cocktail, 2 μM carfilzomib and 4 μl of benzonase per ten 15-cm plates). Lysed extracts were pelleted at 21,000*g* to remove cellular debris and the clarified lysate was bound to anti-Flag M2 resin (20 μl of slurry per 2.5 15-cm plates of material) for 2 h with rotation at 4 °C. UBR4-coupled beads were washed twice with (40 mM HEPES, pH 7.5, 5 mM KCl, 150 mM NaCl, 0.1% NP-40, 1 mM DTT) and twice without (40 mM HEPES, pH 7.5, 5 mM KCl, 150 mM NaCl, 1 mM DTT) detergent, all liquid was removed from the beads using a crushed gel loading tip before addition of the in vitro ubiquitylation reaction.

In vitro ubiquitylation assays were performed in a 10 μl reaction volume. 0.5 μl of 10 μM E1 (250 nM final), 0.5 μl of 50 μM Ube2A (2.5 μM final), 0.5 μl of 50 μM Ube2D3 (2.5 μM final), 1 μl of 10 mg ml^−1^ ubiquitin (1 mg ml^−1^ final) (R&D Systems, U-100H), 0.5 μl of 200 mM DTT, 1.5 μl of energy mix (150 mM creatine phosphate (Sigma-Aldrich, 10621714001-5G), 20 mM ATP, 20 mM MgCl_2_, pH to 7.5 with NaHCO_3_), 1 μl of 10× ubiquitylation assay buffer (250 mM Tris, pH 7.5, 500 mM NaCl and 100 mM MgCl_2_) and 0.5 μl of 1 mg ml^−1^ tandem ubiquitin binding entities were pre-mixed and added to 10 l of UBR4-coupled bed resin. Then, 3 μl of in vitro translated substrate and 1 μl of 100 μM TAMRA-labelled peptide were added to the reactions. In the ABHD10 ubiquitylation experiment, where ABHD10–3×HA was immunoprecipitated with Flag–UBR4, and in the KCMF1 auto-ubiquitylation experiment, where KCMF1–3×Flag (and mutants) were immunoprecipitated from cells, no additional substrate was added. PBS was added to reach final volume of 10 μl. Peptide sequences used in this study are summarized in Supplementary Table 3. Reactions were performed at 30 °C with shaking for 2 h or as indicated in the respective time course. Reactions were stopped by adding 2× urea sample buffer and resolved on SDS–PAGE gels before autoradiography in the case of radiolabelled substrates. MTS-TAMRA peptide ubiquitylations were run on 4–20% gradient gels (Thermo Fisher Scientific, EC6026BOX) and imaged on the ProteinSimple Fluorchem M imager. ABHD10–3×HA or KCMF1–3×Flag ubiquitylations were visualized by western blotting with anti-HA or anti-ubiquitin antibodies, respectively. Lys11/48-branched ubiquitin chains were visualized using a bispecific antibody developed previously^17^. The following commercially available recombinant human ubiquitin mutants used in this study were used: R&D Systems, UM-K11R, UM-K27R, UM-K29R, UM-K33R, UM-K48R, UM-K480, UM-K63R, UM-NOK. L8A ubiquitin was purified as described previously^48^. E1 enzyme UBA1 was purified as described previously^74^. Ube2A and Ube2A mutants, Ube2D3 and Tube recombinant proteins were purified as previously described^4^.

### Protein purification for NMR analysis

For NMR isotopic labelling, WT untagged ubiquitin was expressed in *Escherichia coli* BL21 (DE3) grown in M9 minimal medium supplemented with ^15^N-ammonium chloride (Cambridge Isotope Labs). Ubiquitin was purified by cation exchange followed by SEC using the Superdex 75 column (GE Healthcare) equilibrated in NMR buffer (25 mM NaPi, pH 7.0, 150 mM NaCl, 1 mM TCEP) as previously described^75^. 6×His-tagged UBR4-UBL (residues 4340–4463; UniProt: Q5T4S7-1) variants were expressed in *E. coli* BL21 (DE3) and purified by Ni^2+^-NTA affinity chromatography using the Cytiva HisTrap FF crude column according to the manufacturer’s instructions followed by SEC using the Superdex 75 column equilibrated in NMR buffer.

### NMR

All NMR samples were assembled with 200 mM ^15^N-labelled ubiquitin and the indicated amounts of unlabelled UBR4-UBL in NMR buffer supplemented with 10% deuterium oxide. ^1^H-^15^N heteronuclear single quantum coherence (HSCQ) spectra were collected on the Bruker Ascend 500 mHz magnet equipped with a Bruker Avance IV NEO console and a 5 mm BBO Prodigy CryoProbe. Data were transformed and phased using Bruker TopSpin (4.3) and plotted in NMRViewJ. Combined amide (NH) chemical shift perturbations (CSP) were calculated using the following equation: ΔδNH (ppm) = sqrt[ΔδH^2^  +  (ΔδN/5)^2^]. NMR CSP titration data were fitted to the standard equation for a 1:1 binding equilibrium to derive a dissociation constant (*K*_d_) for the ubiquitin–UBL interaction: ΔδNH (ppm) = Δ*δ*_max_ (([*P*]_*t*_ + [*L*]_*t*_ + *K*_d_) − [([*P*]_*t*_ + [*L*]_*t*_ + *K*_d_)^2^ − 4[*P*]_*t*_[*L*]_*t*_]^1/2^)/2[*P*]_*t*_ where [*L*]_*t*_ and [*P*]_*t*_ are the concentrations of ligand (UBR4-UBL) and protein (ubiquitin), respectively^76^. A subset of NMR signals with substantial and well-resolved chemical shifts was used for *K*_d_ calculation. Data were plotted and fit to the binding equilibrium equation using Python. Plotted curves were fit to individual residues, while the reported *K*_d_ represents a global fit to all plotted residues with the s.d. derived from the residual sum of squares.

### Software and code for data analysis

The following freely/commercially available software/codes were used to analyse data: FlowJo (v.10.8.1), GraphPad Prism (v.9), NMRViewJ (v9.2.0-b27), Bruker TopSpin (v.4.3.0), cryoSPARC (v.4.3), SerialEM (v.4.1), 3DFlex, AlphaFold (v.2; v.3), PHENIX (v.1.21.1-5286), Coot (v.0.9.8.92), PDBePISA (v1.52), Chimera (v.1.17.1), ChimeraX (v.1.8), PyMOL (v.2.5.5), Spectronaut (18.0), ProteoWizard’s msConvert (v.3.0.22335), Kojak (v.2.0.3), Percolator (v.2.08) and ProXL web application.

### Reporting summary

Further information on research design is available in the Nature Portfolio Reporting Summary linked to this article.

## Online content

Any methods, additional references, Nature Portfolio reporting summaries, source data, extended data, supplementary information, acknowledgements, peer review information; details of author contributions and competing interests; and statements of data and code availability are available at 10.1038/s41586-025-09074-z.

## Supplementary information

Supplementary Fig. 1 Source data for western Blots, fluorescence gels and autoradiographs.

Reporting Summary

Supplementary Fig. 2 Gating strategy for flow cytometry experiments.

Supplementary Fig. 3 Workflow of EM processing.

Supplementary Table 1 Source data for proteomics data.

Supplementary Tables 2 and 3 Supplementary Table 2: plasmids. Supplementary Table 3: synthetic peptide sequences.

Supplementary Video 1 Overview of SIFI structure and important domains. Structures shown in the video are based on PDB 9D9Z and AlphaFold models.

Supplementary Video 2 Movements of the SIFI scaffold around a central hinge region analysed by cryoSPARC 3D flexible refinement using particle images of EM map EMD-46742.

Supplementary Video 3 Flexibility of the substrate-binding cavity of SIFI. Data were analysed by cryoSPARC 3D flexible refinement using particle images of EM map EMD-46686.

Supplementary Video 4 Movements of the SIFI arms. Data were analysed by cryoSPARC 3D flexible refinement using particle images of EM map EMD-49876.

## Figures and Tables

**Extended Data Table 1:**
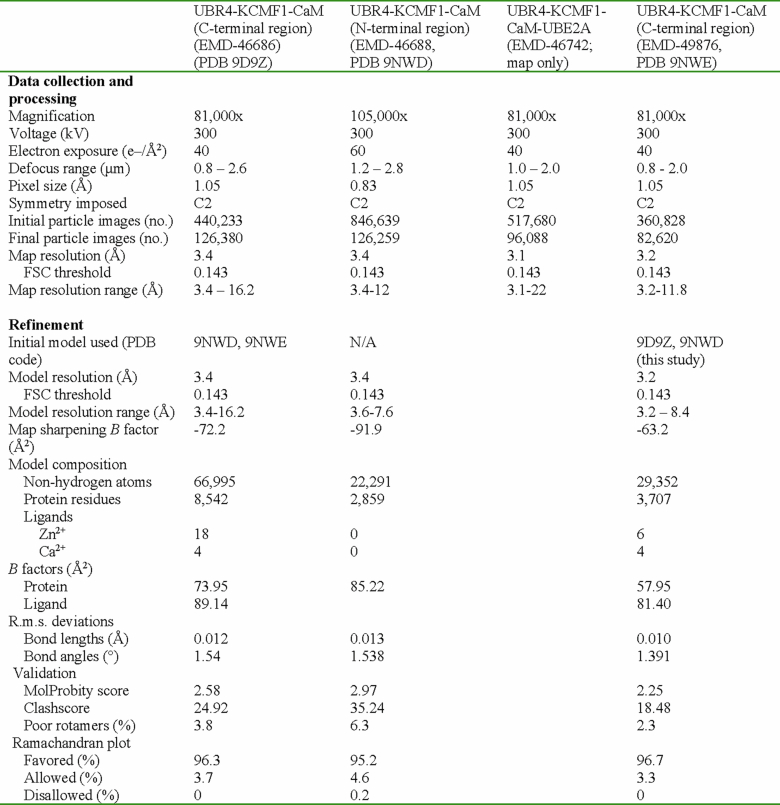
Cryo-EM data collection, refinement and validation statistics

## Data Availability

The consensus atomic coordinate model and cryo-EM map of the human UBR4–KCMF1–CaM (SIFI) complex has been deposited in the Protein Data Bank (PDB) and Electron Microscopy Data Bank (EMDB) under accession codes 9D9Z and EMD-46686, respectively. Coordinates and cryo-EM maps of the endogenous SIFI complex (C-terminal partial map with improved local densities) has been deposited under accession codes PDB 9NWE and EMD-49876, and the SIFI complex purified through affinity-tagged KCMF1 (N-terminal partial map) has been deposited under accession codes PDB 9NWD and EMD-46688. The cryo-EM map of the SIFI complex supplemented with UBE2A has been deposited under EMDB accession code EMD-46742. Source data for immunoblots are provided in Supplementary Fig. 1. Gating strategies for flow cytometry experiments are provided in Supplementary Fig. 2. The workflow for cryo-EM structure generation is provided in Supplementary Fig. 3. Source data for the IP–MS datasets are provided in Supplementary Table 1. Cross‐linking MS data are available interactively on the ProXL web application^73^ (https://proxl.yeastrc.org/proxl/p/sifi-hri) along with the raw MS spectra and search parameters used. Moreover, the complete search algorithm configuration files, fasta search databases, raw search output and raw MS data files were deposited to the ProteomeXchange Consortium via the PRIDE partner repository (https://www.ebi.ac.uk/pride/archive) under dataset identifier PXD055759. The publicly available CRAPome and Mitocarta 3.0 datasets can be accessed at https://reprint-apms.org and https://www.broadinstitute.org, respectively. There are no restrictions on data availability.
