# Supplementary material for: Molecular basis of SIFI activity in the integrated stress response
Source: Nature. 2025 May 6;643(8073):1117–26. doi: 10.1038/s41586-025-09074-z (PMC12286842; doi:10.1038/s41586-025-09074-z)
Supplement: Supplementary file 1 — Source data for western Blots, fluorescence gels and autoradiographs. [file 41586_2025_9074_MOESM1_ESM.pdf]

nature portfolio

https://doi.org/10.1038/s41586-025-09074-z

Supplementary information

Molecular basis of SIFI activity in the integrated stress response

In the format provided by the authors and unedited

Figure 2e

Chemiluminescence

Chemiluminescence+ visible light

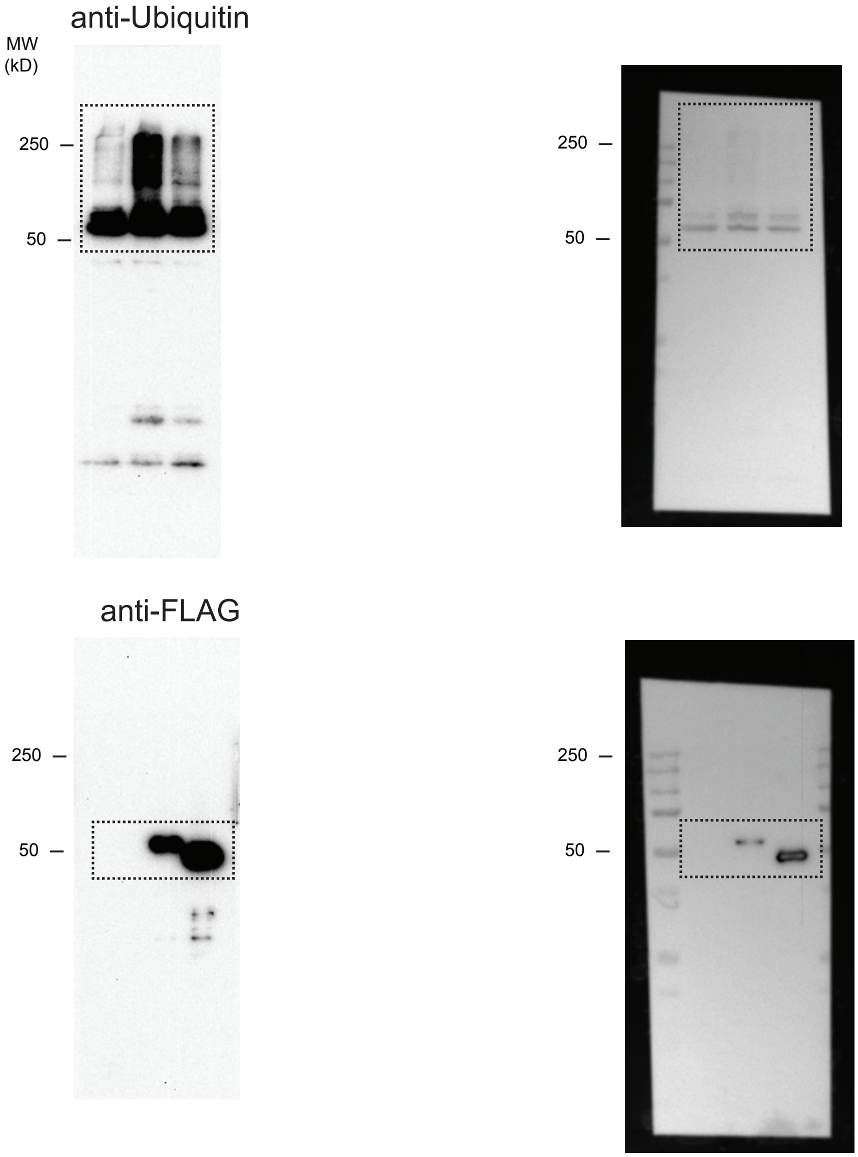

Run on different blots

Figure 2g

INPUT

Chemiluminescence | **Chemiluminescence + visible light**

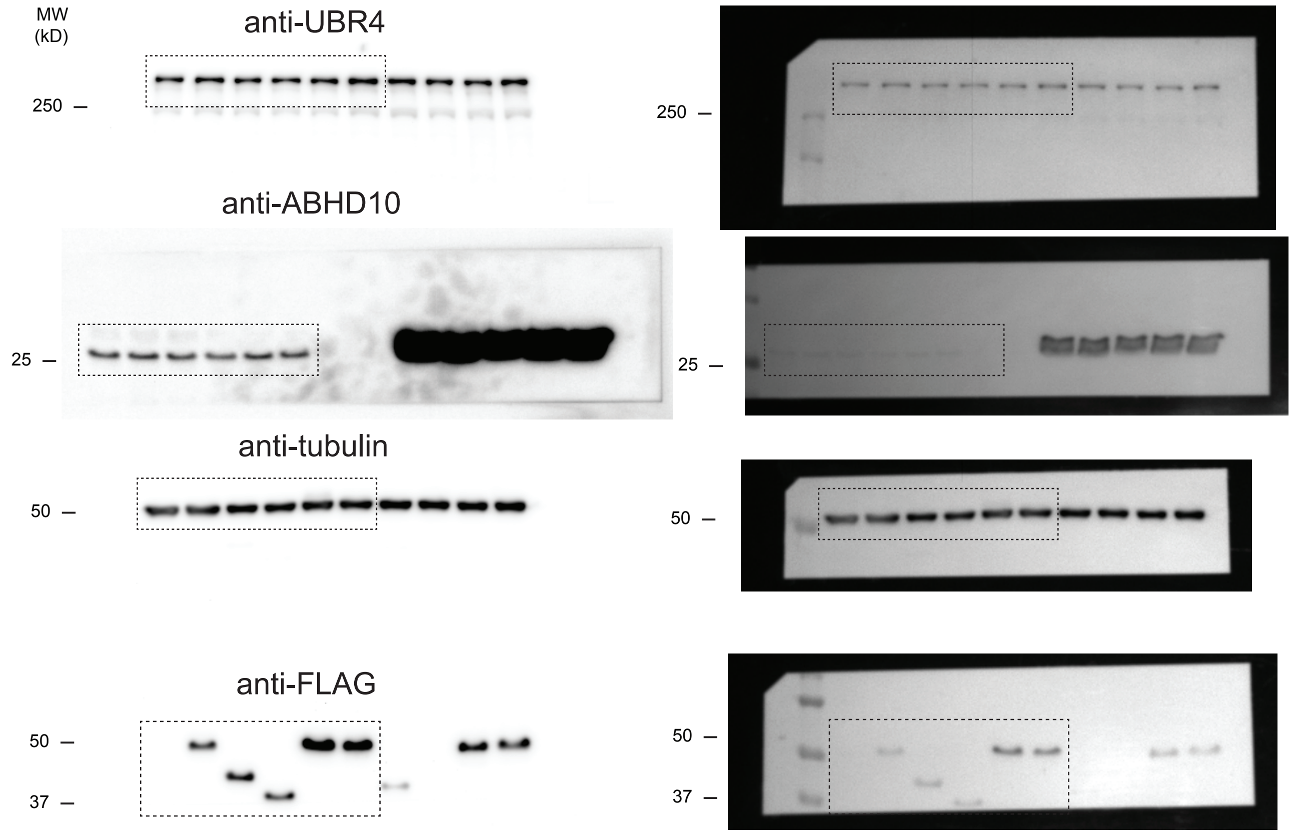

IP

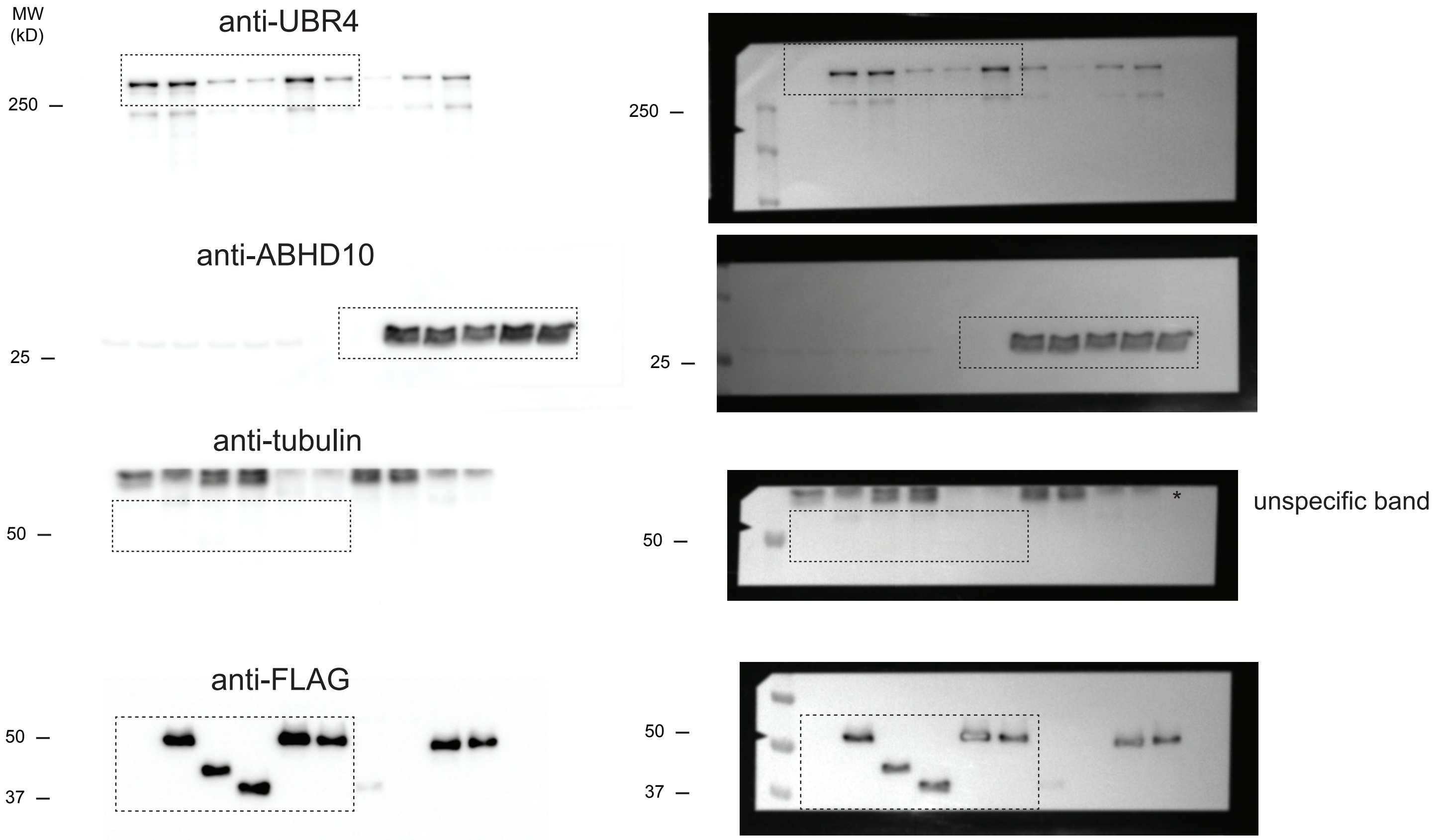

The loading control tubulin was run on the same gel as UBR4.

Figure 3c

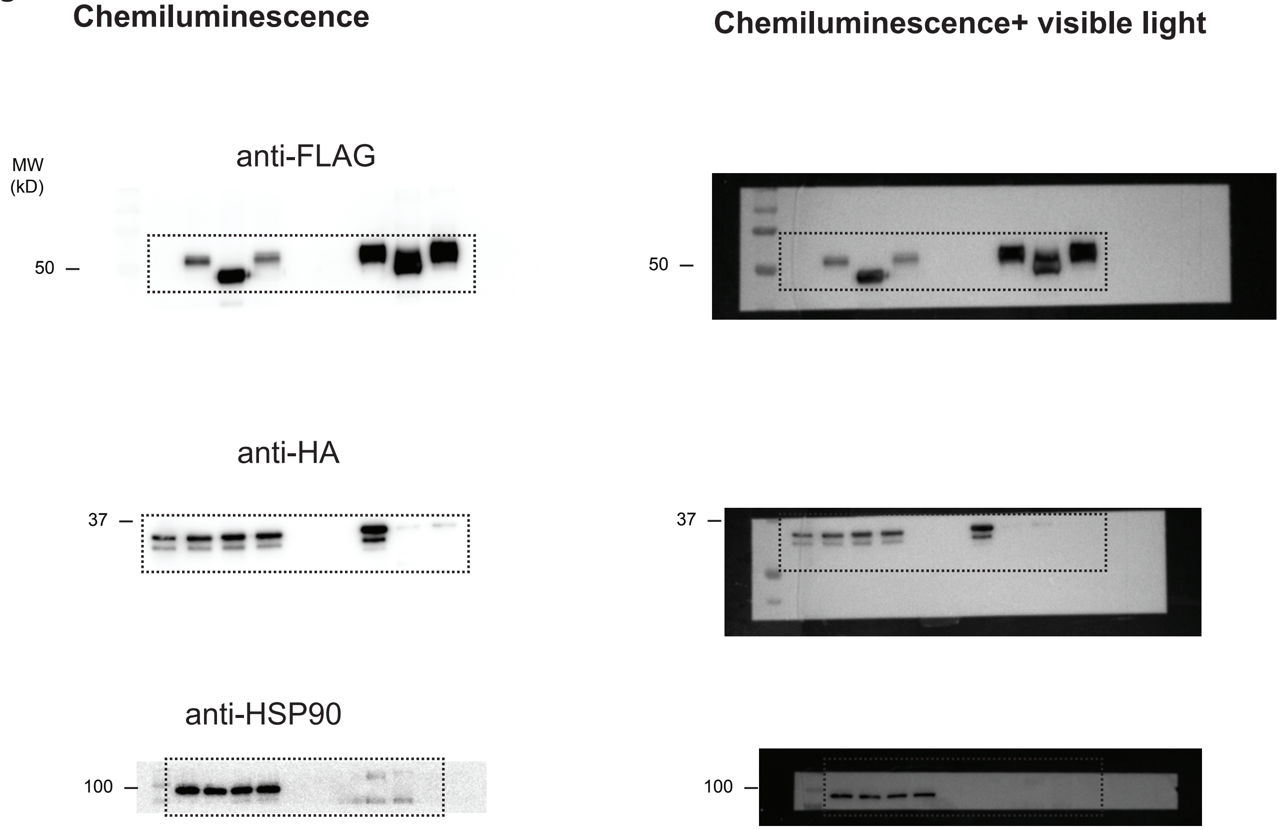

The loading control HSP90 was obtained by cutting and re-probing the anti-HA immmunoblot with anti-HSP90 antibody and is therefore run on the same gel as FLAG and HA.

Figure 3f

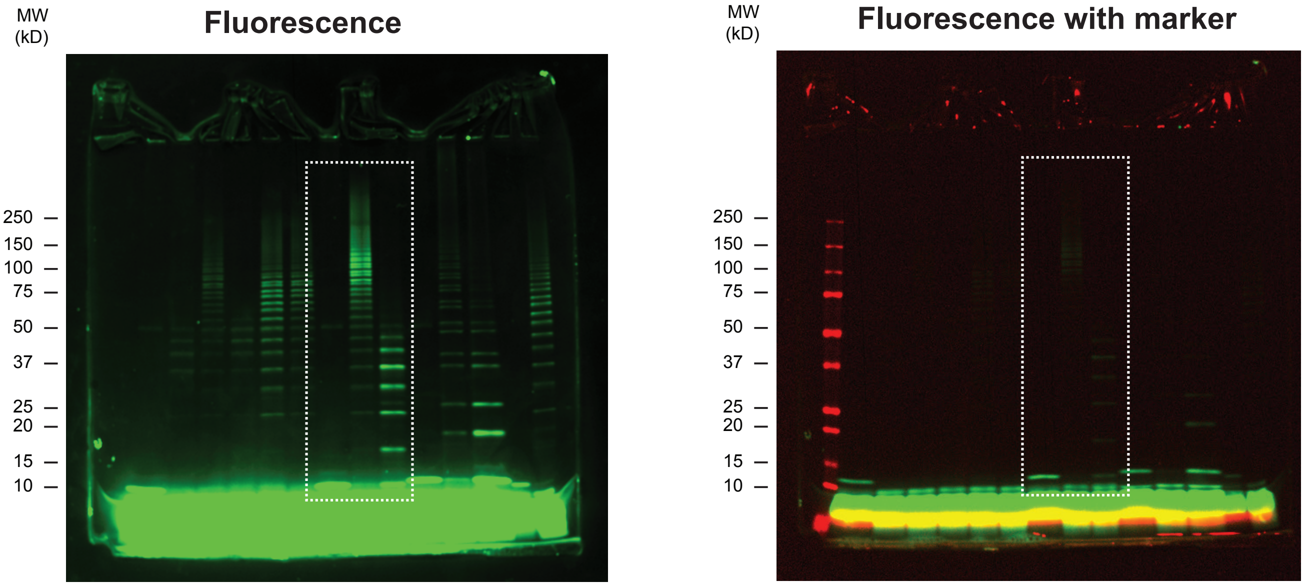

Figure 3h

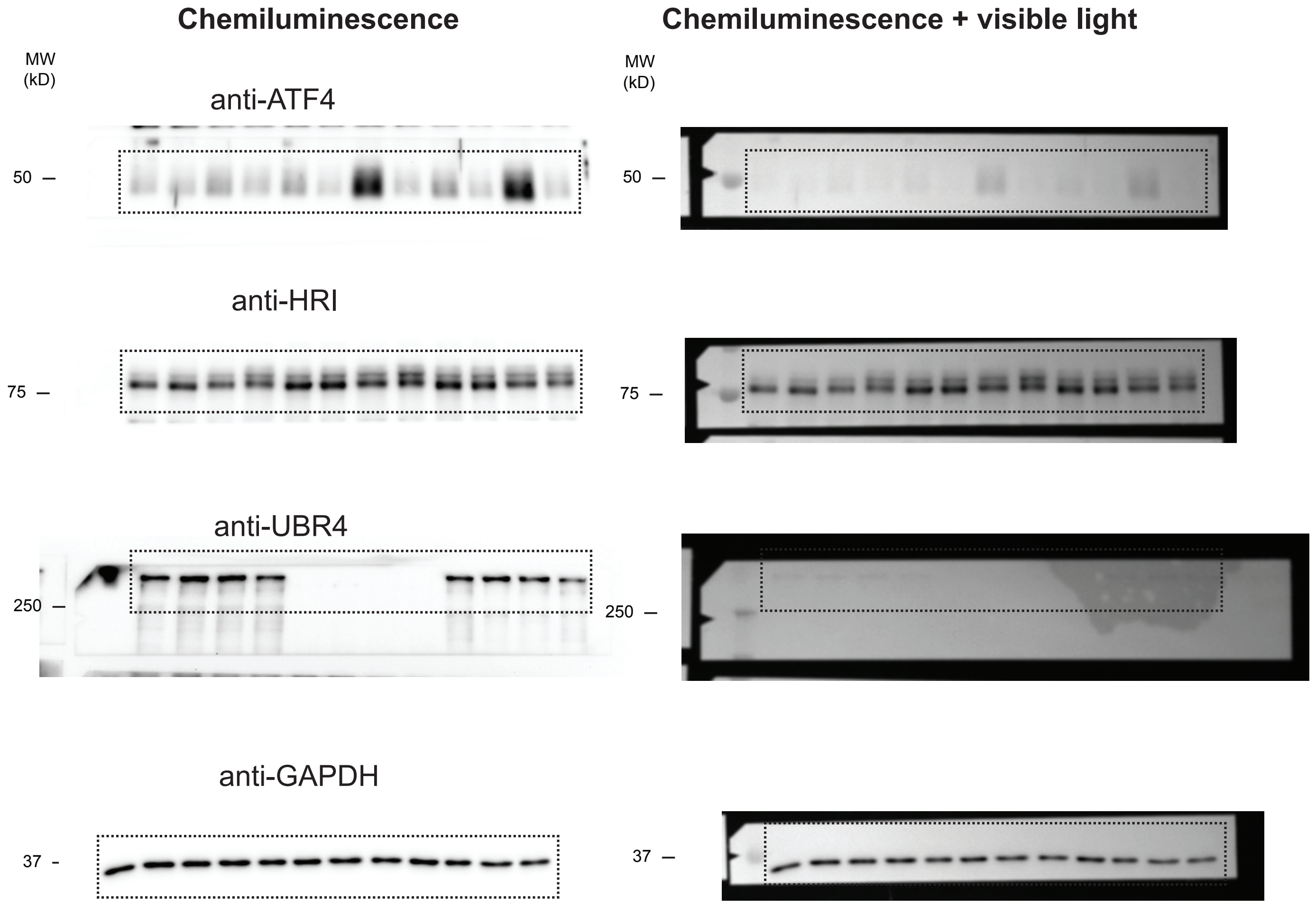

The loading control GAPDH was run on the same gel as ATF4, UBR4 and HRI.

Figure 4b

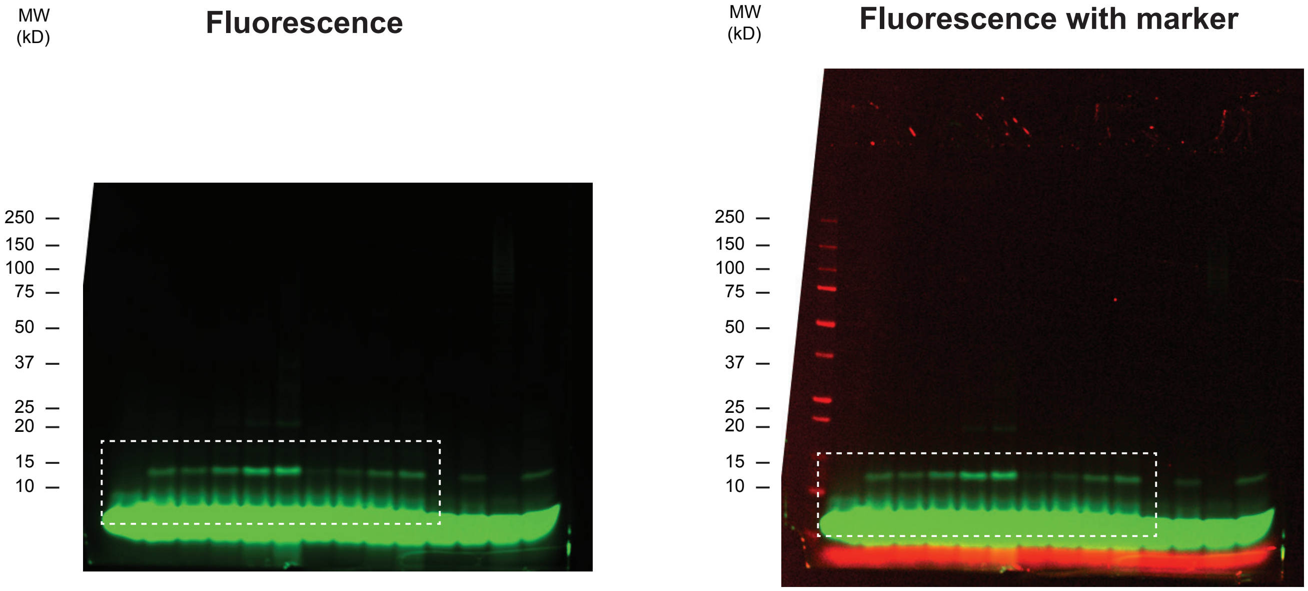
MW (kD)
Fluorescence
250
150
100
75
50
37
25
20
15
10
MW (kD)
Fluorescence with marker
250
150
100
75
50
37
25
20
15
10

Figure 4c

autoradiography

HRI(1-138)-sumo HRI(1-138)-sumo-1xUb

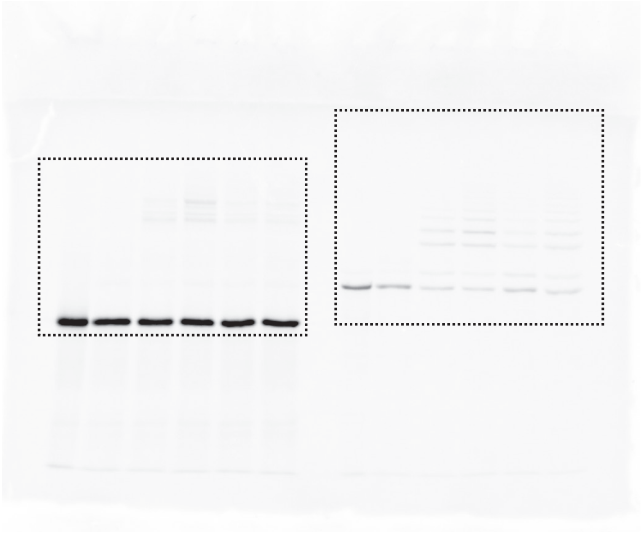

HRI(1-138)-sumo-2xUb

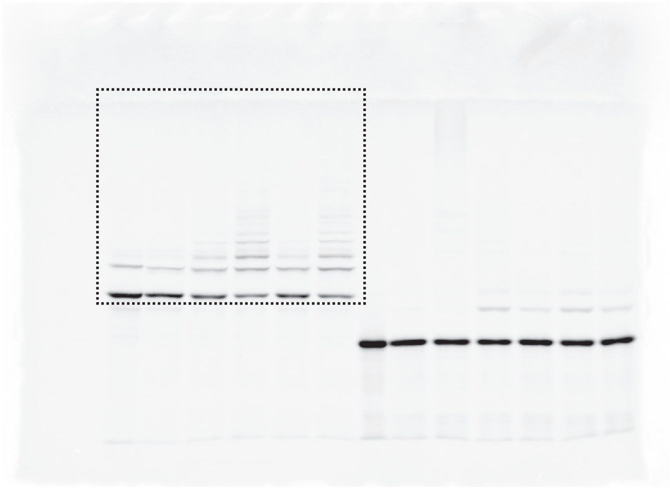

Figure 4e

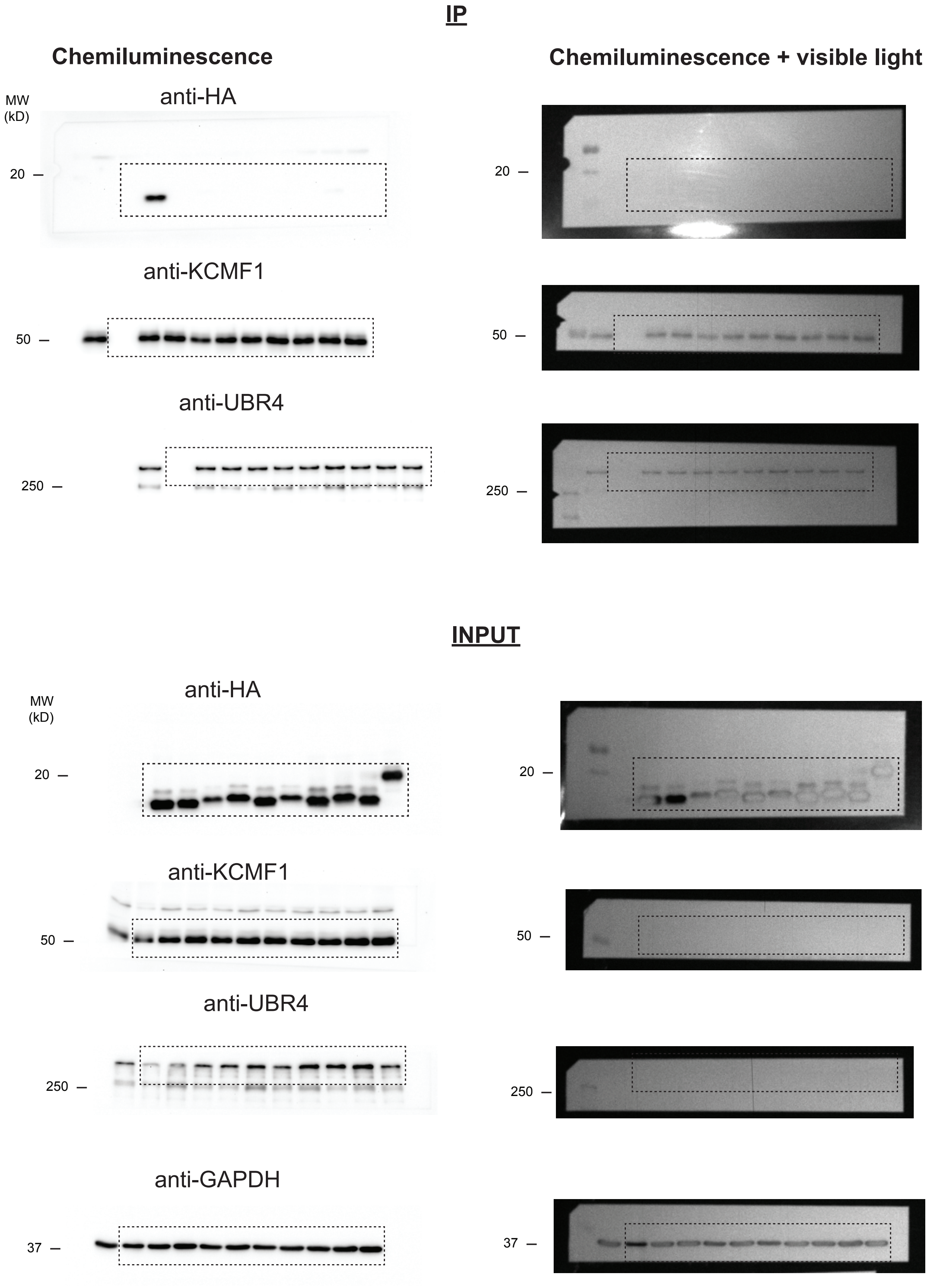

The loading control GAPDH was run on the same gel as UBR4 and KCMF1.

Figure 4f

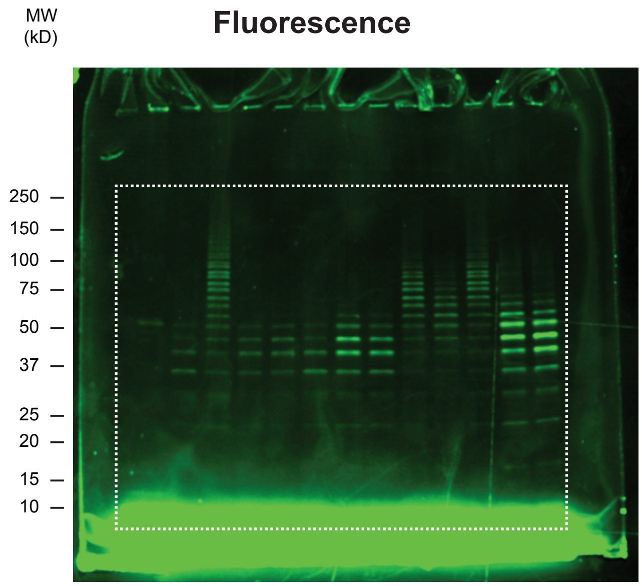

MW (kD)
Fluorescence
250
150
100
75
50
37
25
20
15
10

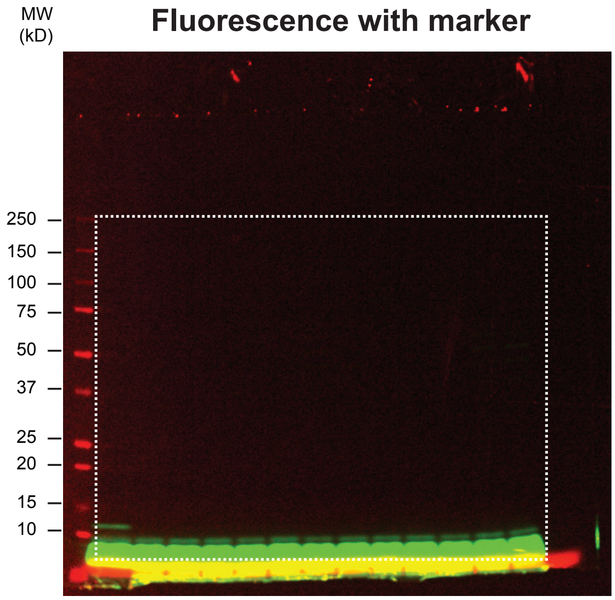

MW (kD)
Fluorescence with marker
250
150
100
75
50
37
25
20
15
10

Figure 5e

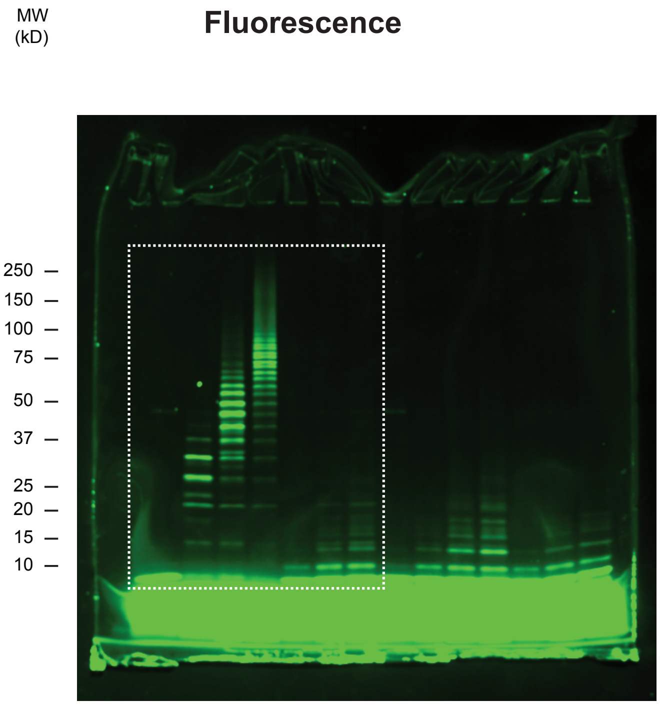
MW
(kD)
Fluorescence
250
150
100
75
50
37
25
20
15
10

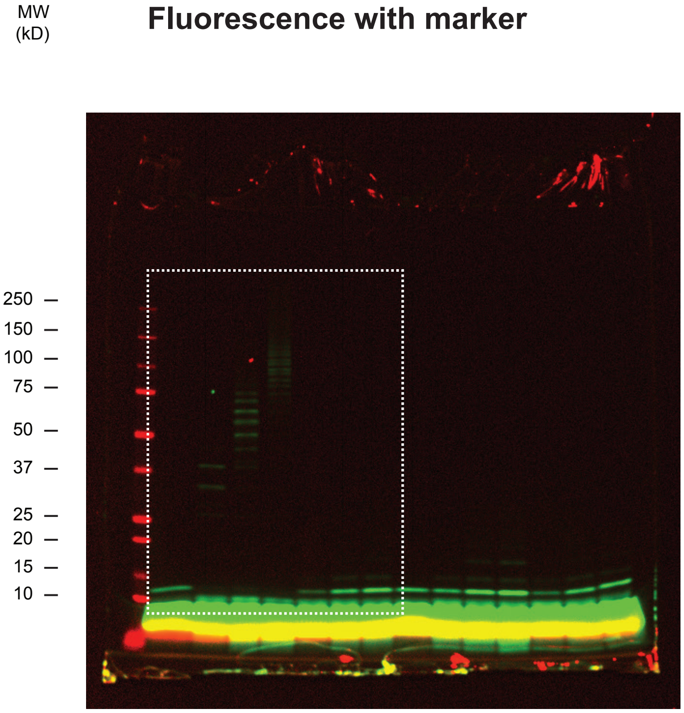
MW
(kD)
Fluorescence with marker
250
150
100
75
50
37
25
20
15
10

Figure 5f

Raw file

HRI-sumo autoradiography

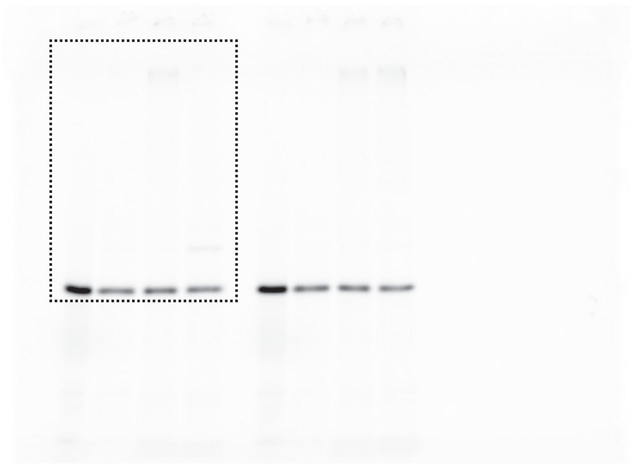

Contrasted raw file to discern typically low S^{35} autoradiography signal

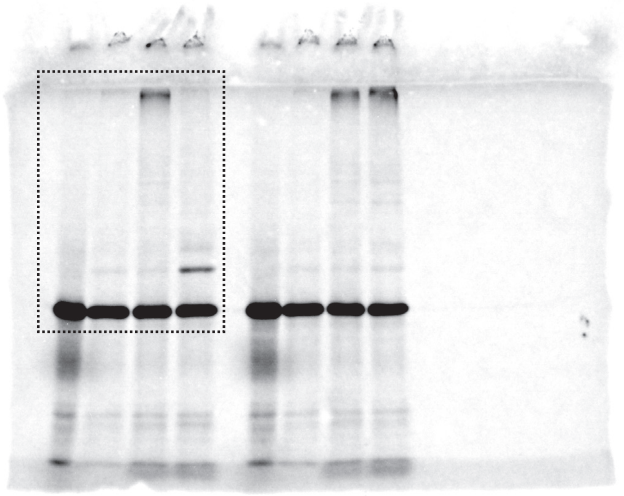

Figure 5h

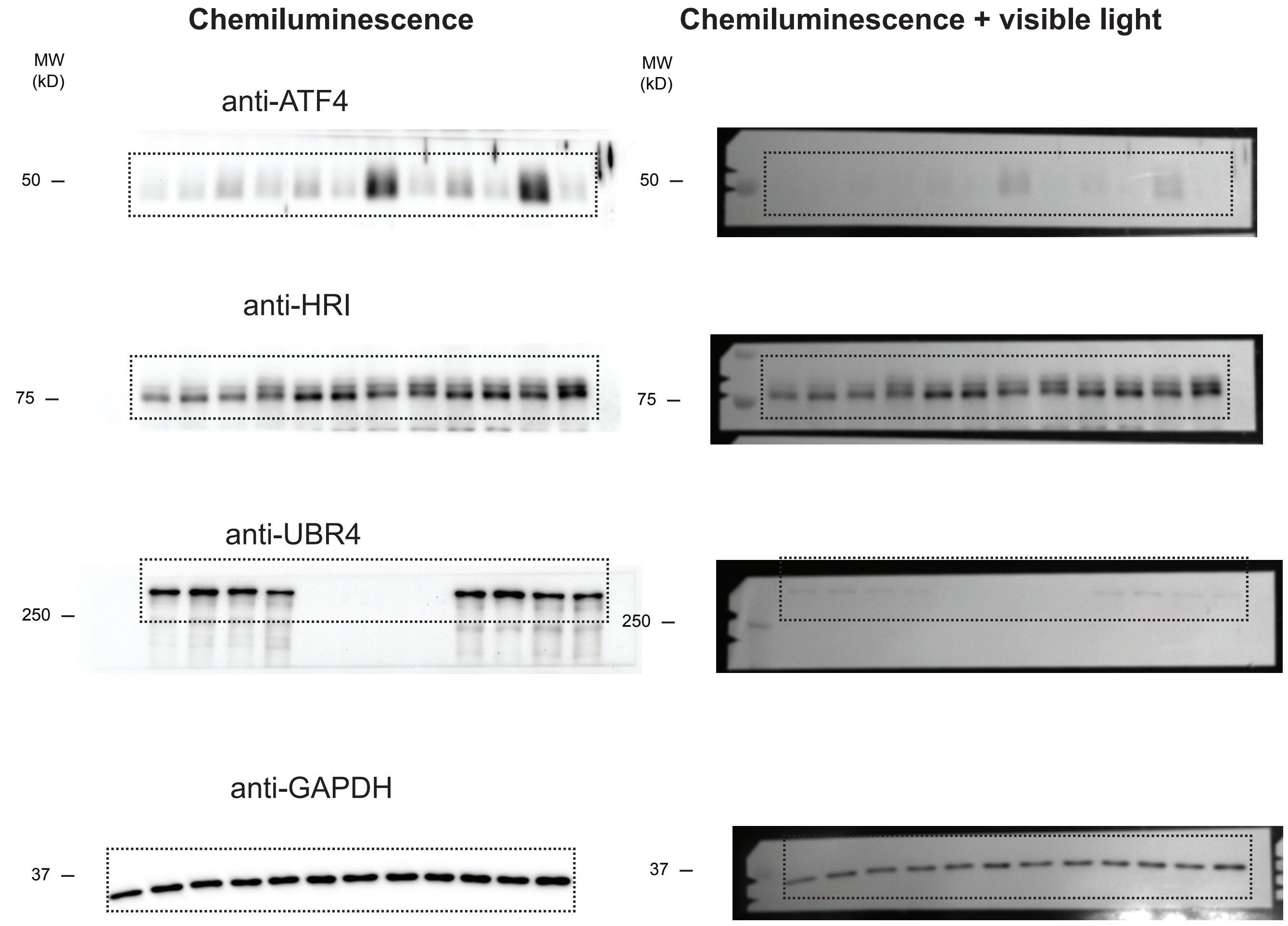

The loading control GAPDH was run on the same gel as ATF4, UBR4 and HRI.

Extended Data Figure 1b

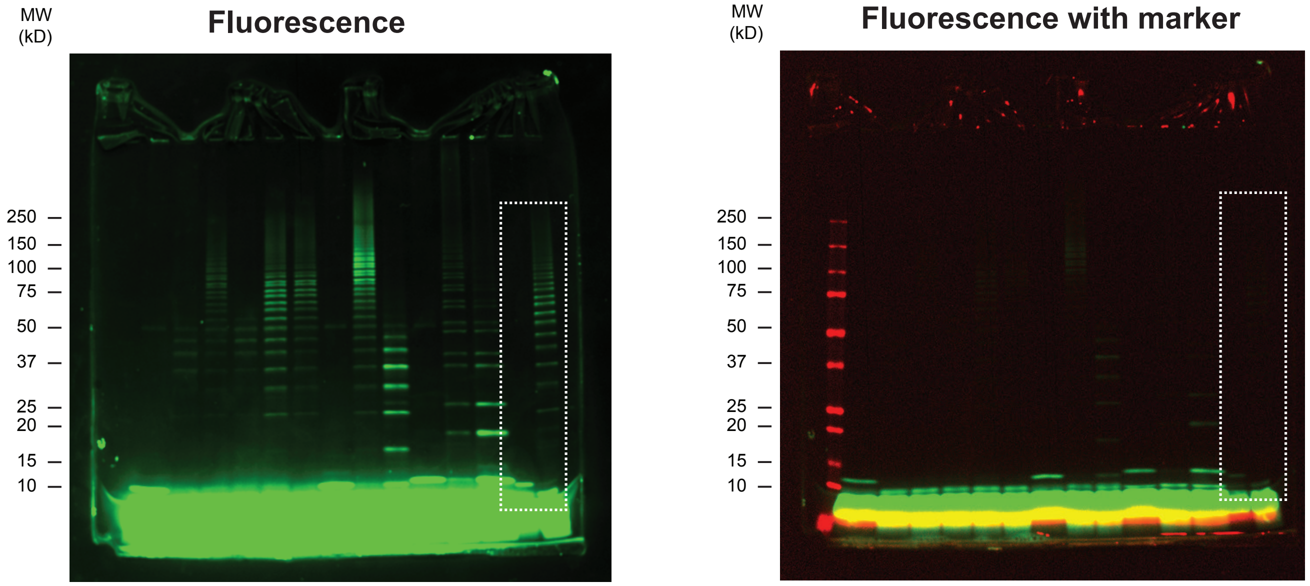

Extended Data Figure 2d

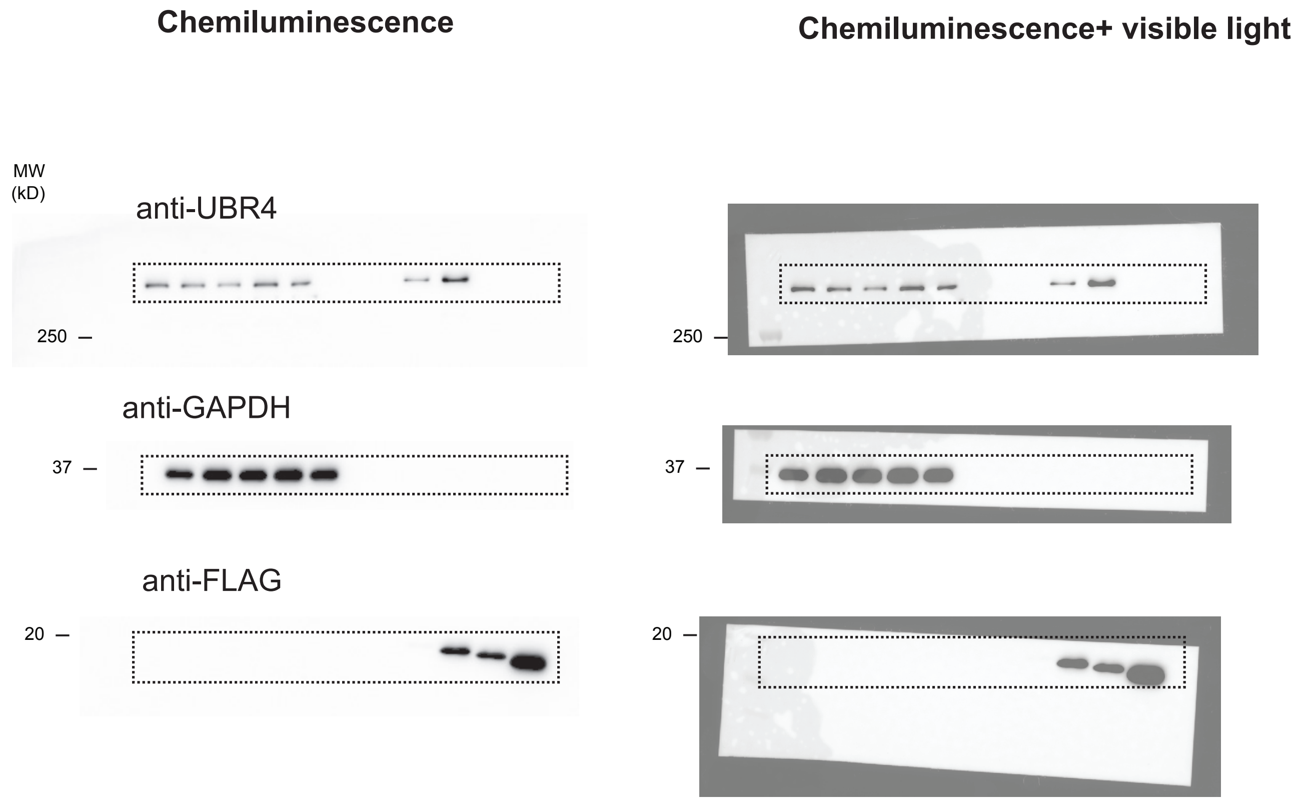

Same blot for all three antibodies.

Extended Data Figure 3d

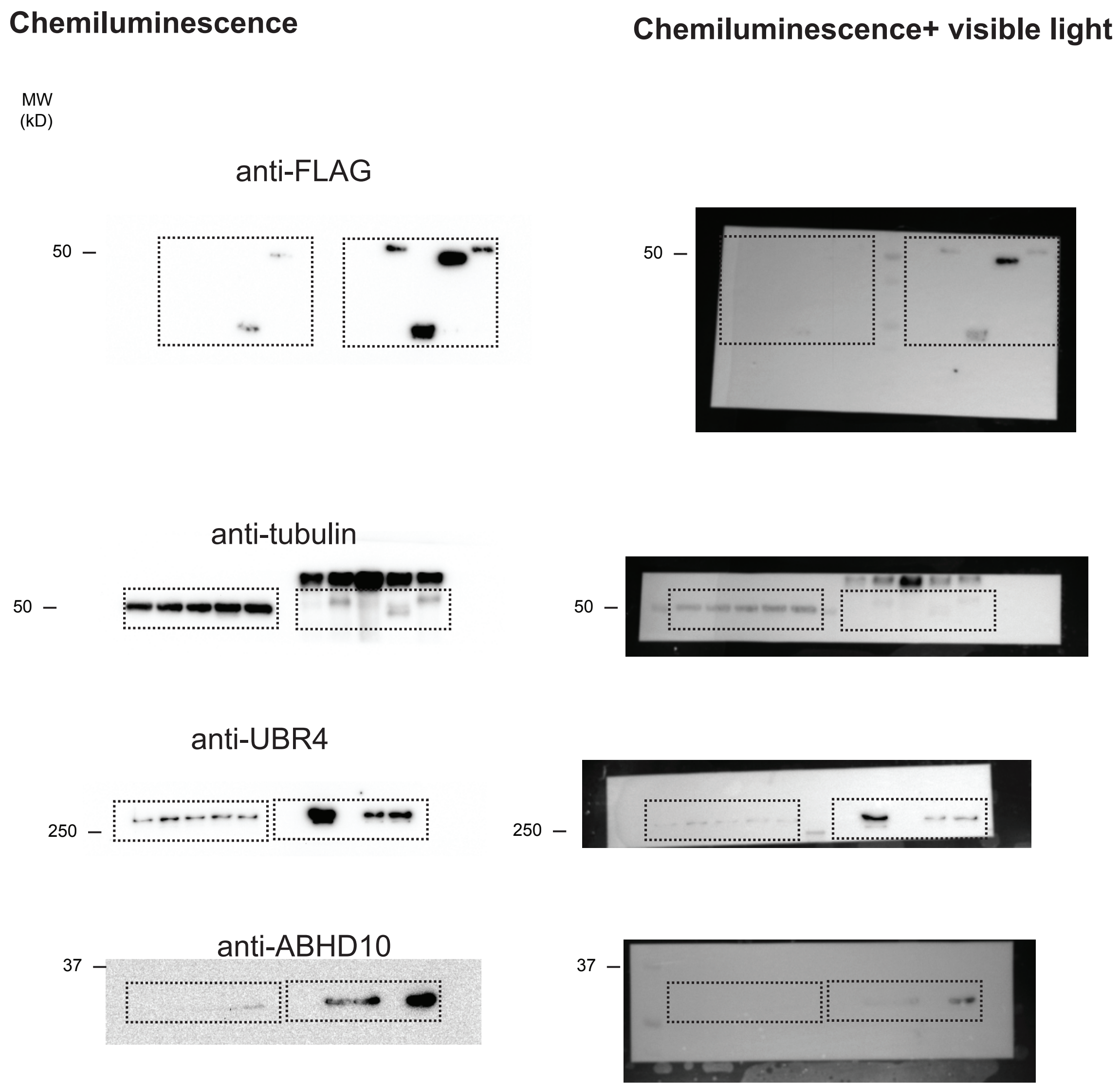

FLAG and UBR4 are from the same blot, tubulin and abhd10 are probed on different blots.

Extended Data Figure 4c

Chemiluminescence

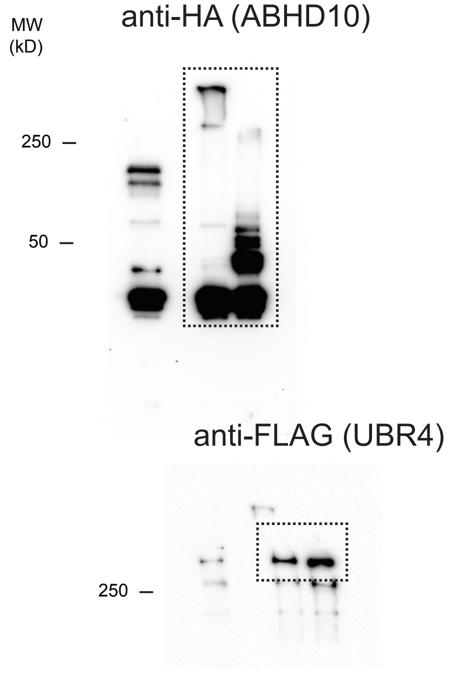

Run on different blots

Chemiluminescence+ visible light

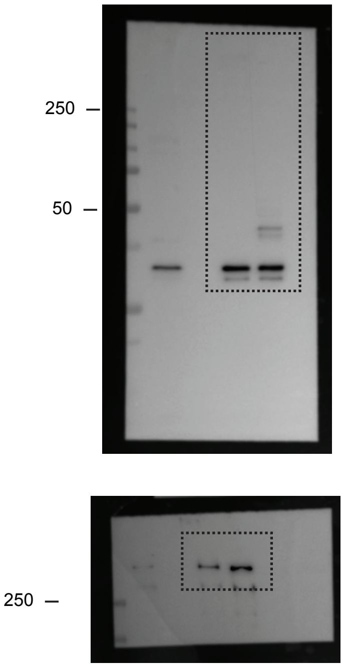

Extended Data Figure 4d

MW
(kD)

Chemiluminescence

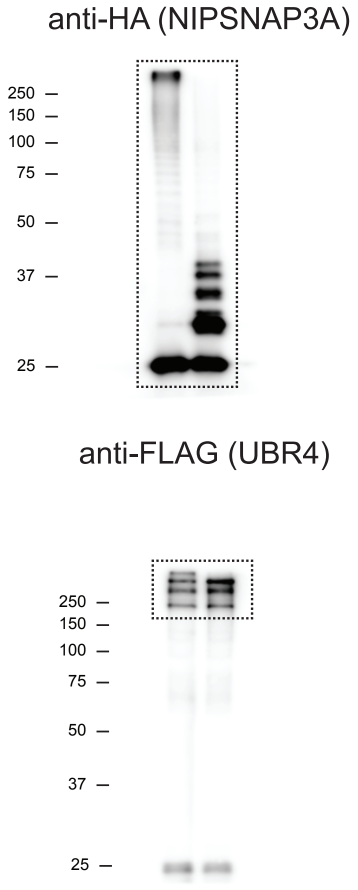

Run on different blots

MW
(kD)

Chemiluminescence + visible light

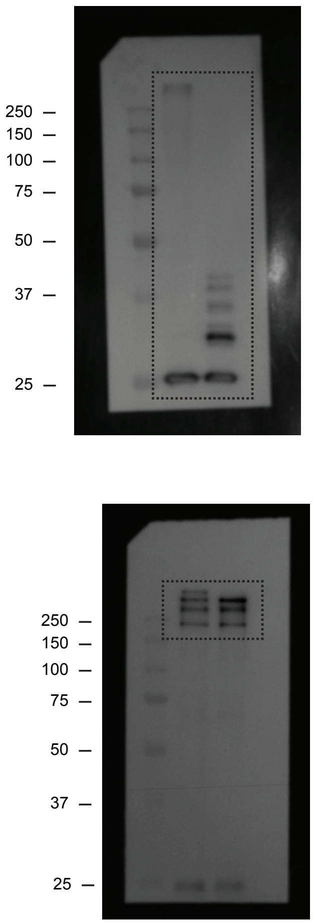

Extended Data Figure 4e

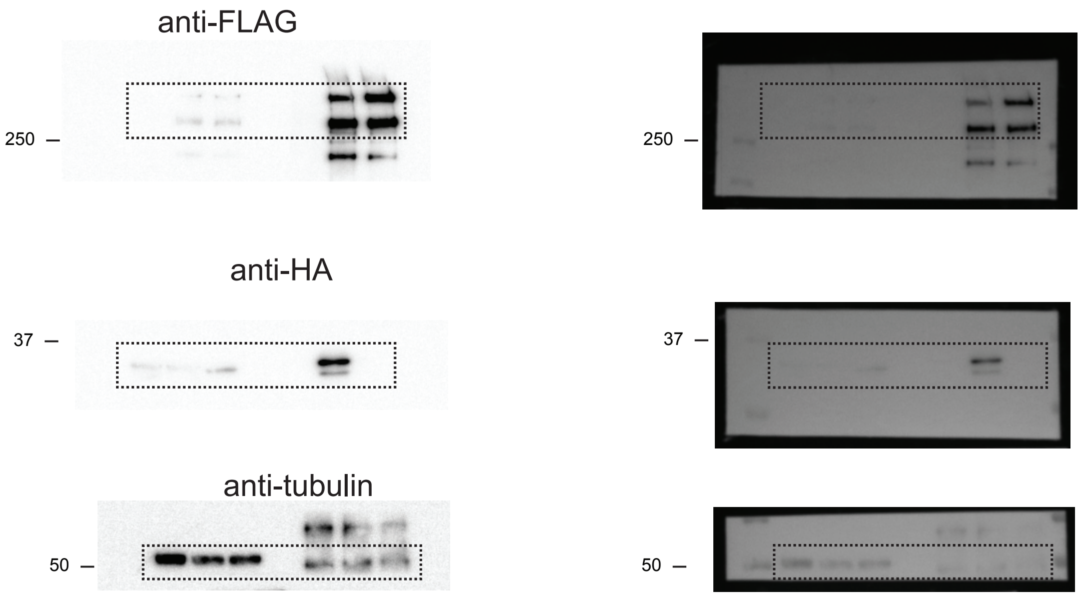

Same blot for all three antibodies.

Extended Data Figure 4f

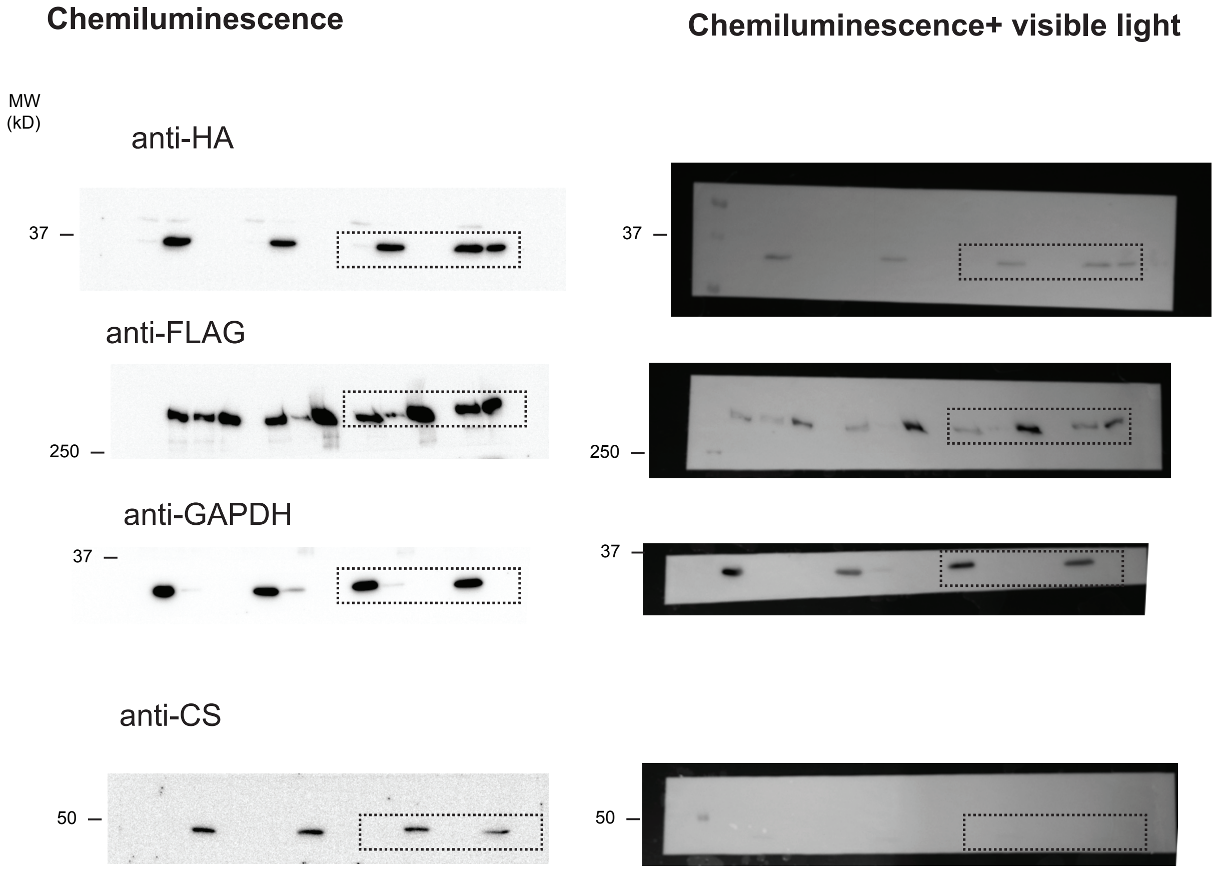

Same blot for HA and FLAG. GAPDH and CS are run on same blot.

Extended Data Figure 5c

Chemiluminescence

Chemiluminescence+ visible light

anti-Ubiquitin

MW (kD)

250 —

50 —

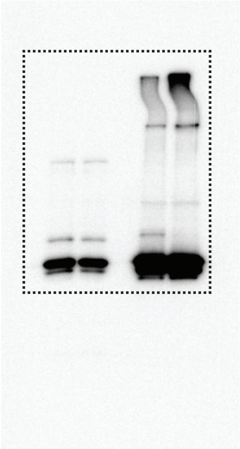

250 —

50 —

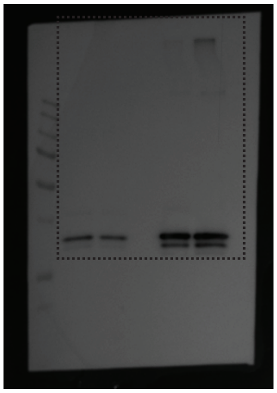

anti-FLAG

250 —

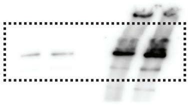

250 —

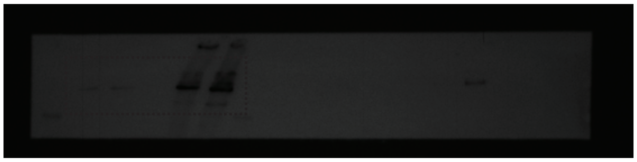

Extended Data Figure 7a

MTS-peptide (right panel)

Fluorescence

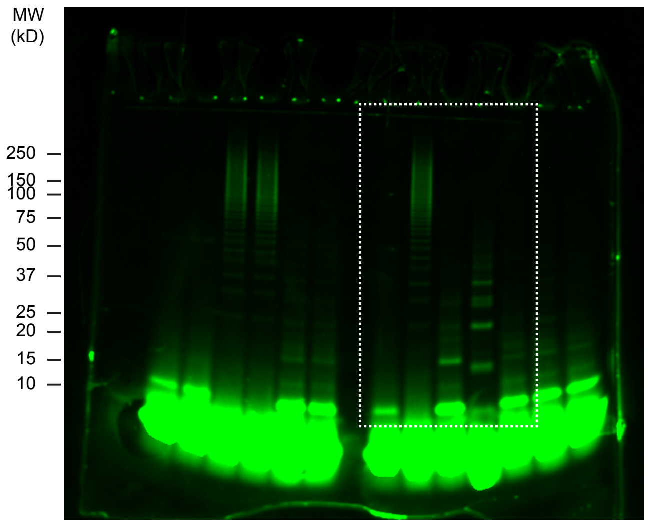

Fluorescence with marker

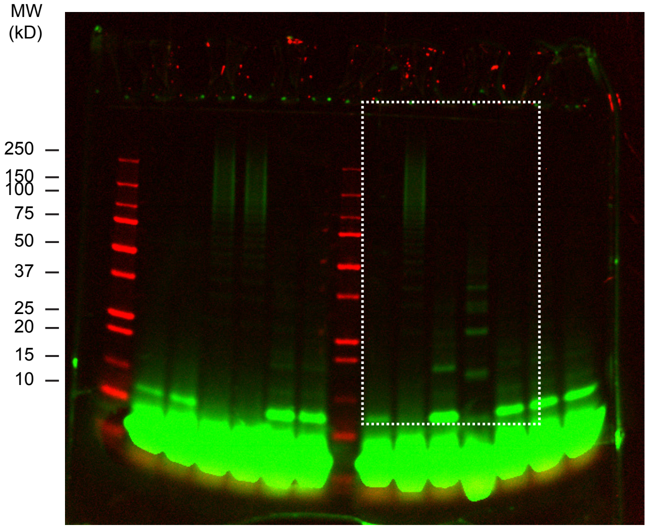

Extended Data Figure 7b

Chemiluminescence	Chemiluminescence + visible light

MW (kD)

anti-Ubiquitin

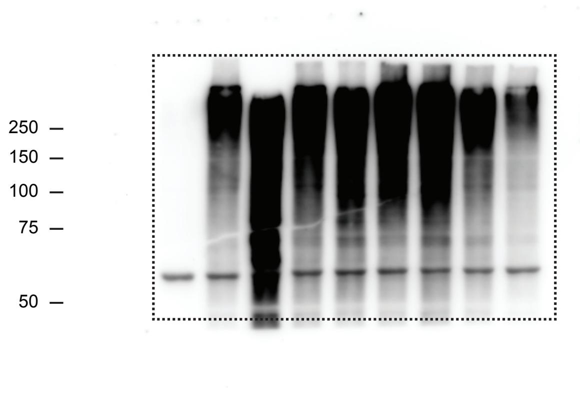

MW (kD)

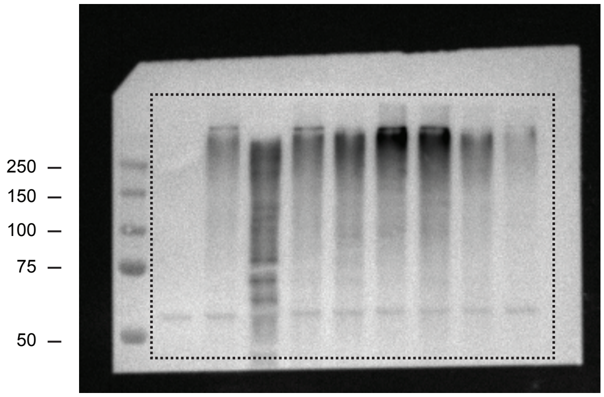

Extended Data Figure 7c

Chemiluminescence	Chemiluminescence + visible light

MW (kD)

anti-Ubiquitin

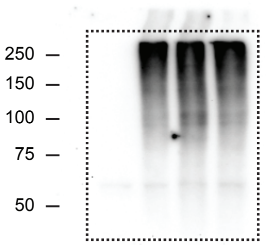

MW (kD)

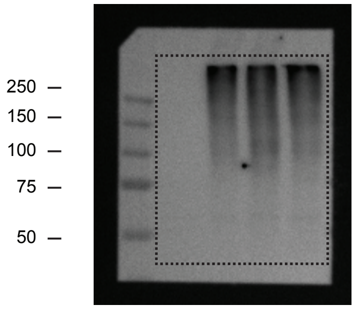

anti-K11/48 branched Ubiquitin

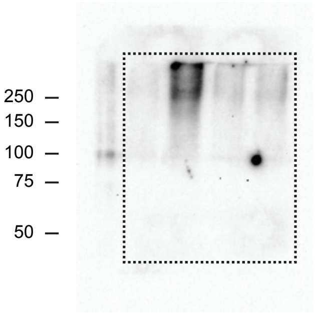

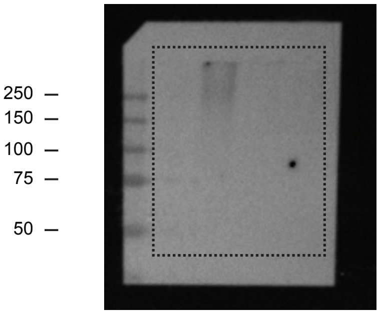

Extended Data Figure 7e

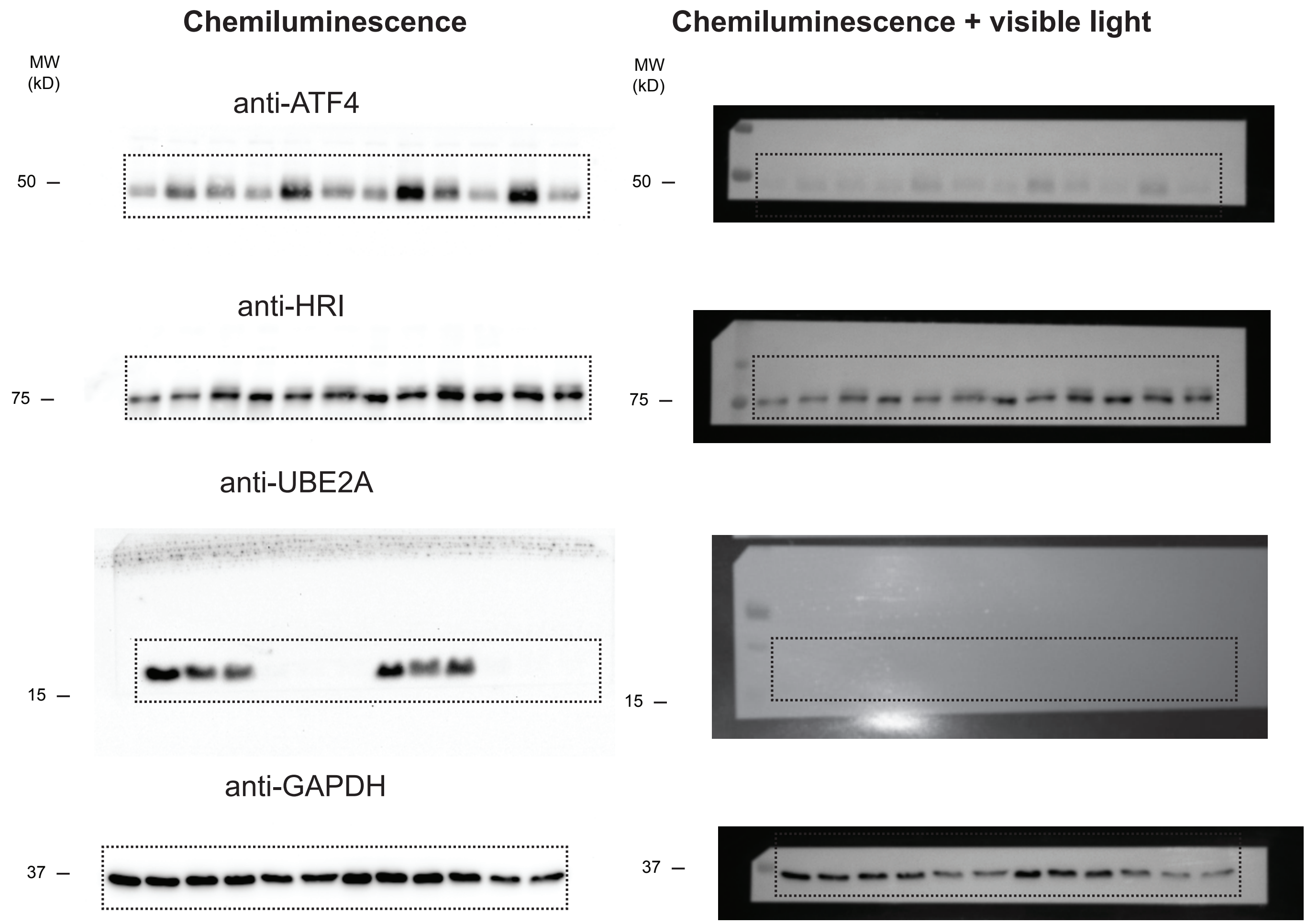

The loading control GAPDH was run on the same gel as ATF4 and HRI.

Extended Data Figure 7f

Fluorescence | **Fluorescence with marker**

MTS-peptide (left panel)

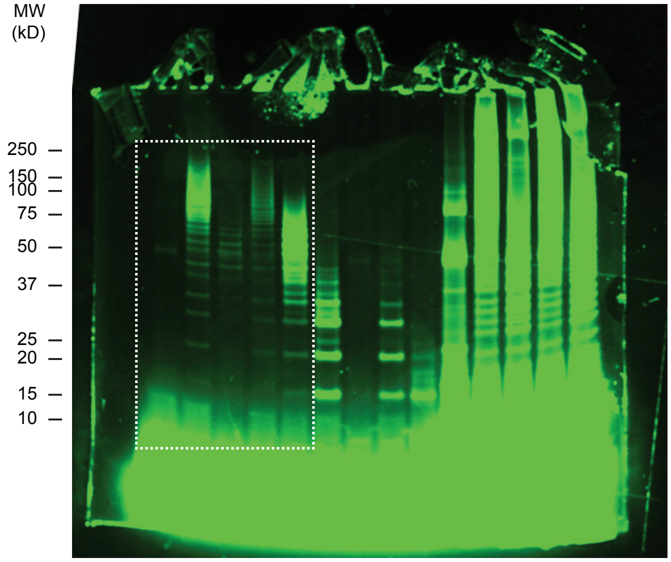

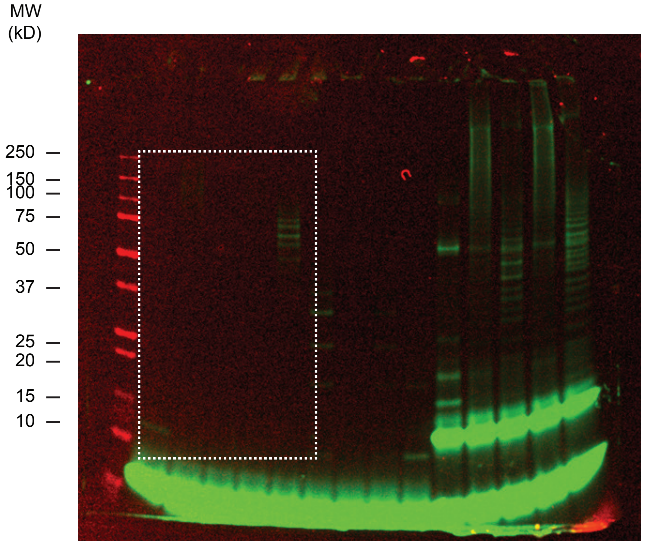

HRI-degron2-peptide (right panel)

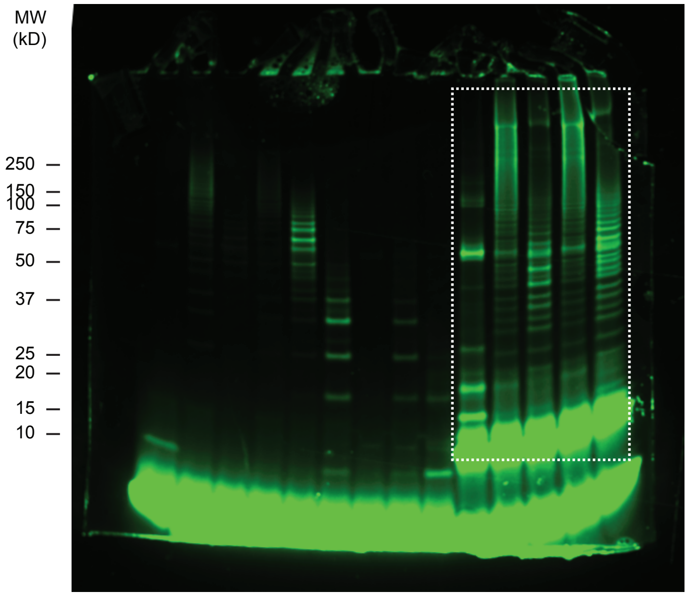

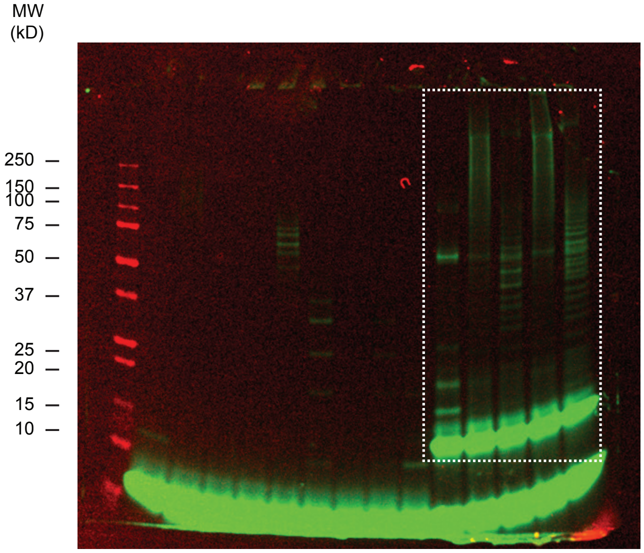

Extended Data Figure 8a

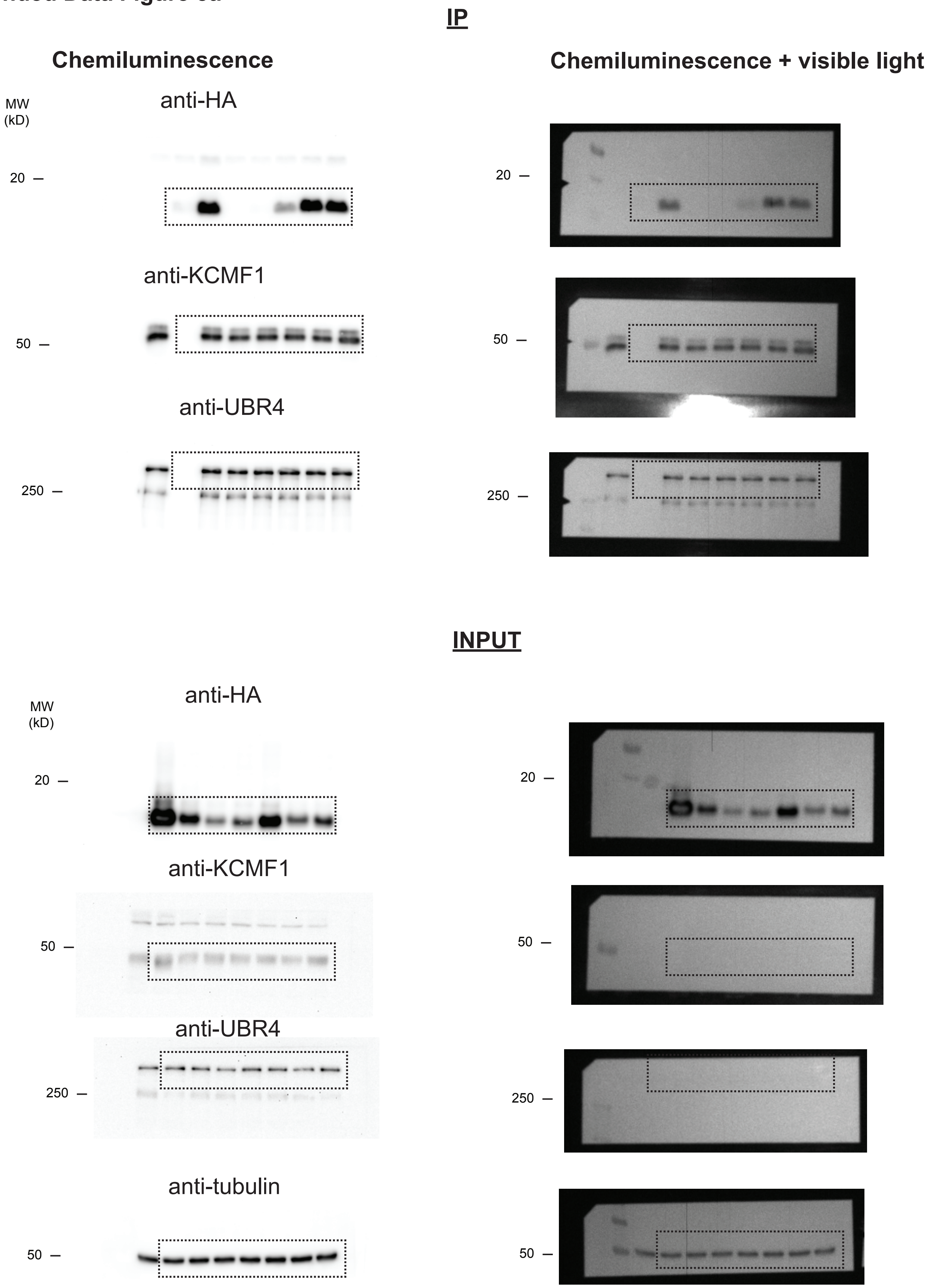

The loading control tubulin was run on the same gel as HA-UBE2A.

Extended Data Figure 8b

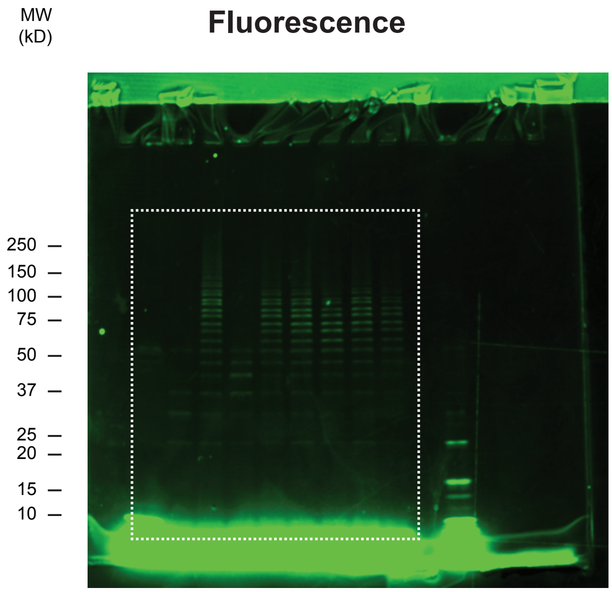

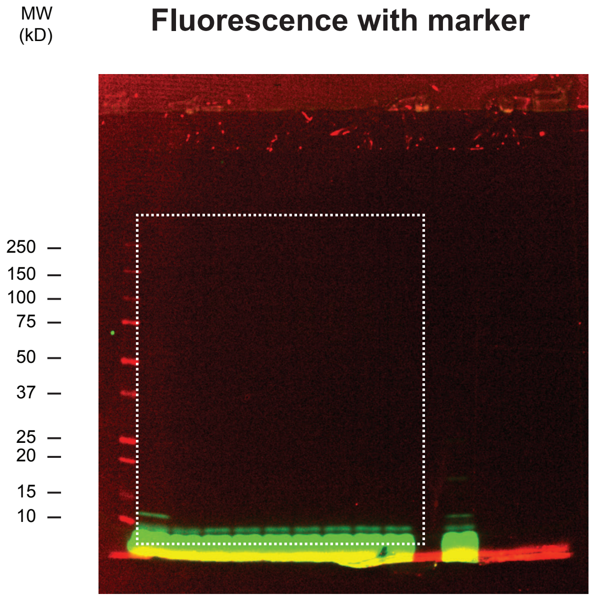

Extended Data Figure 8e

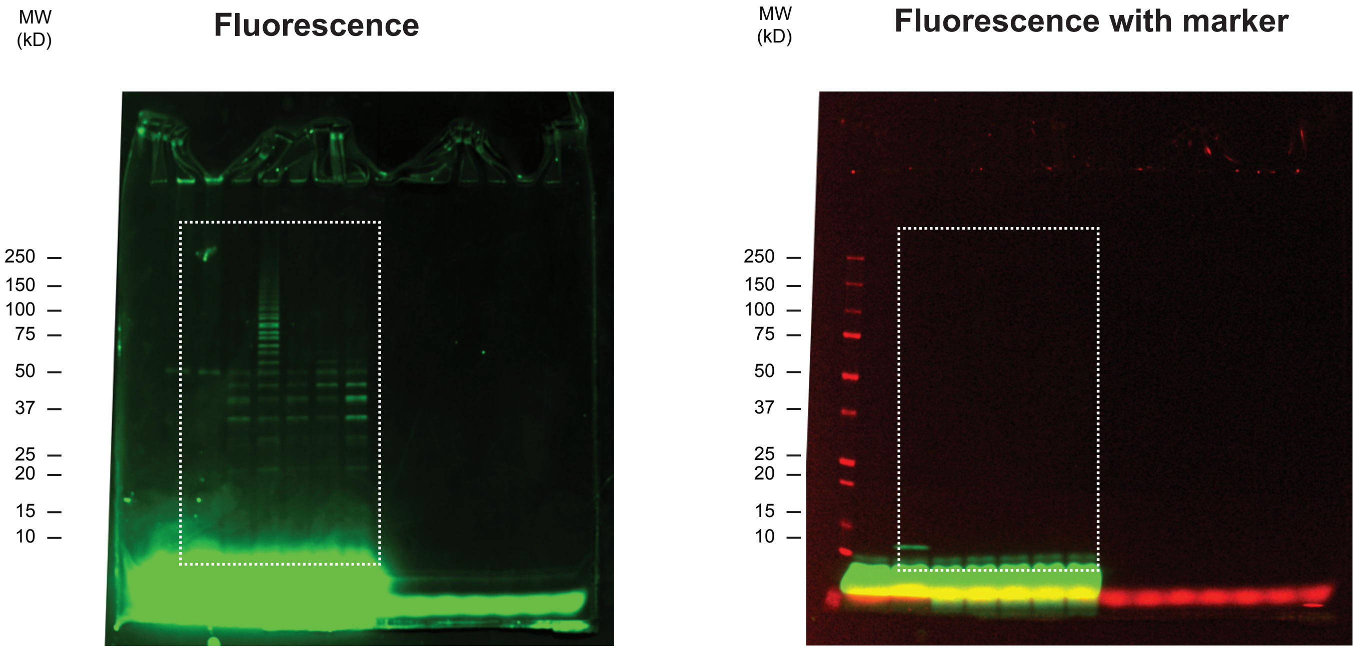

Extended Data Figure 8f

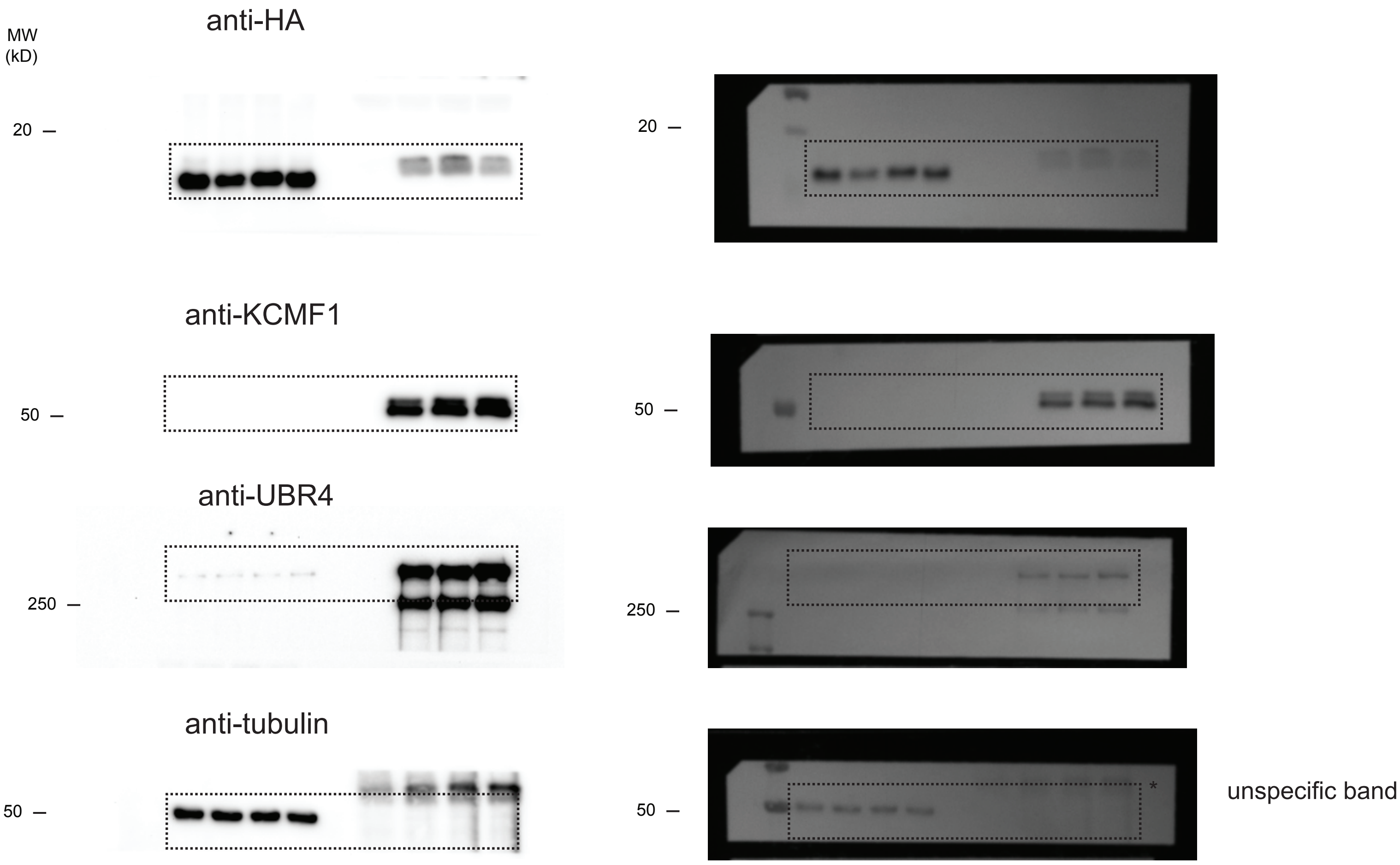

The loading control tubulin was run on the same gel as HA-UBE2A.

Extended Data Figure 9b

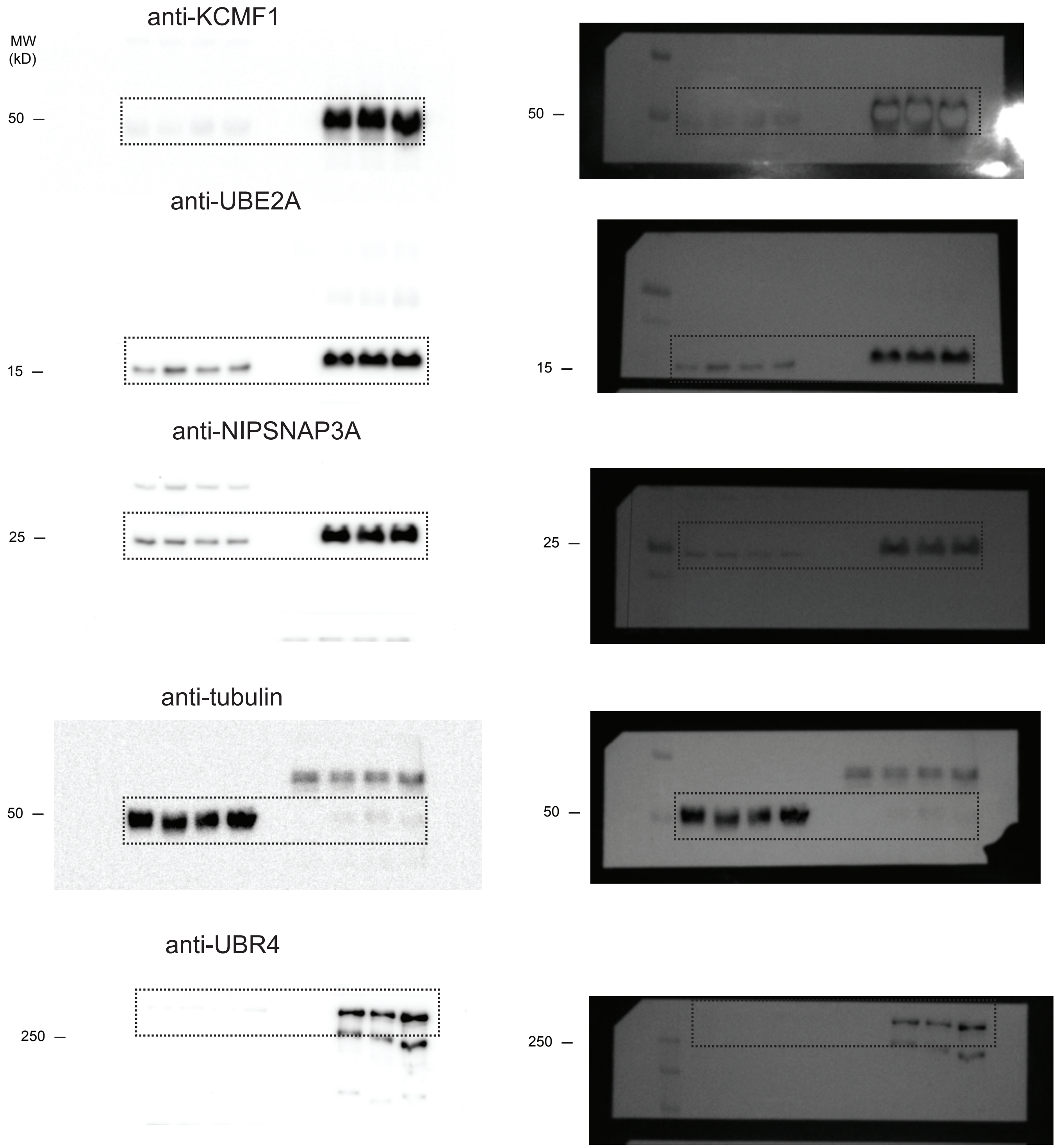

The loading control tubulin was obtained by re-probing the anti-KCMF1 immmunoblot (rabbit-Ab) with a mouse anti-tubulin antibody and is therefore run on the same gel as UBR4 and KCMF1.

Extended Data Figure 9c

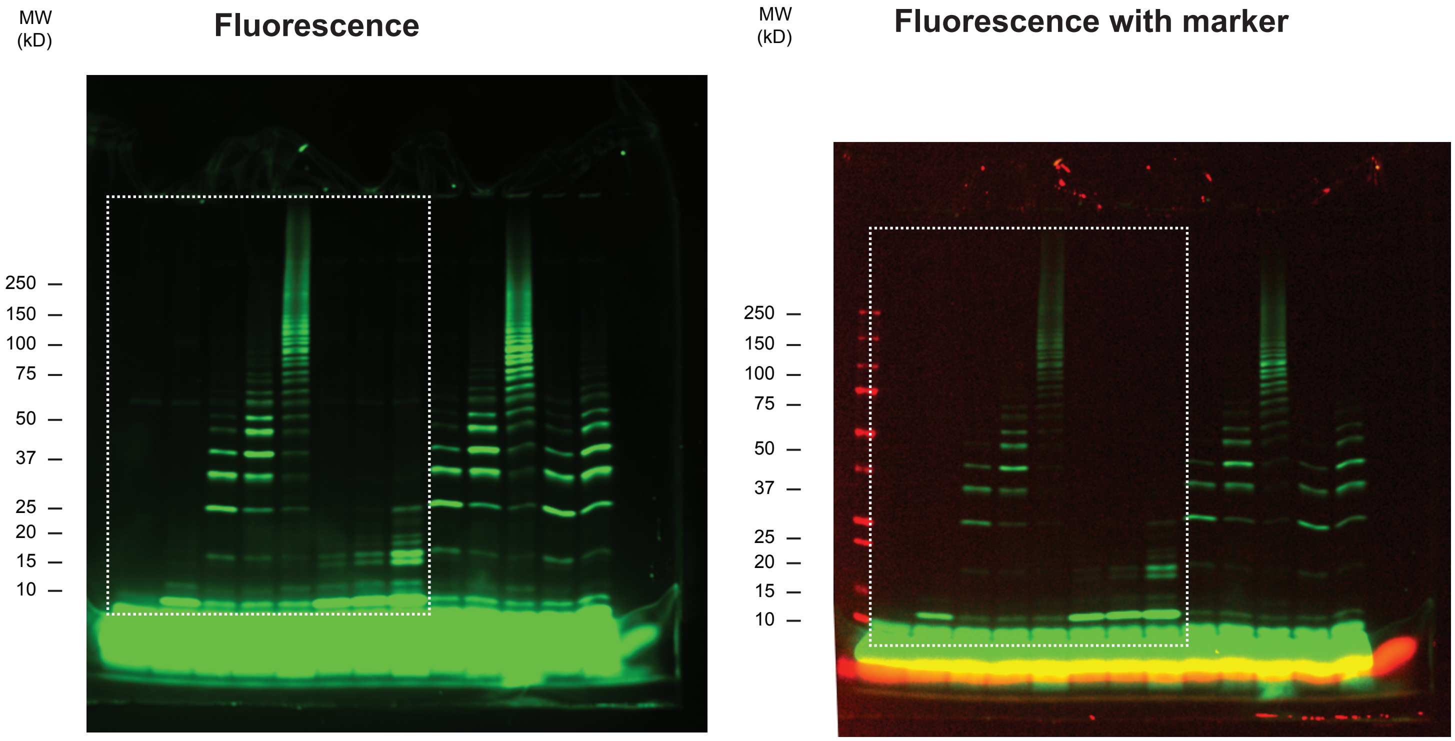

Extended Data Figure 9d

Raw file

HRI-sumo autoradiography

Contrasted raw file to discern typically low S^{35} autoradiography signal

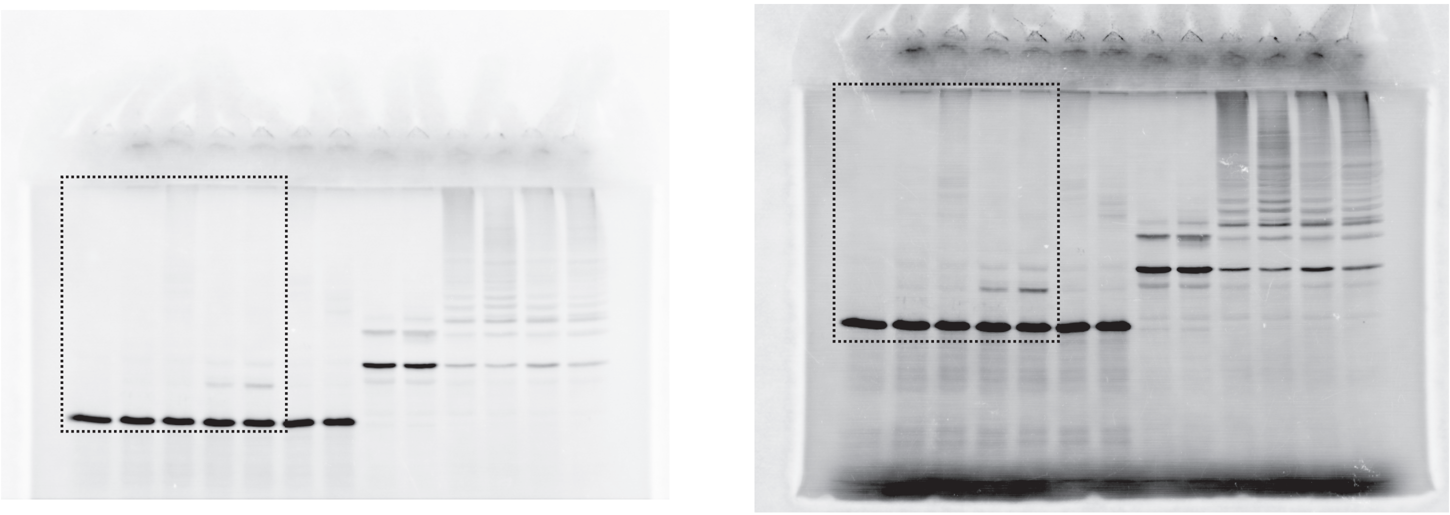

Extended Data Figure 9e

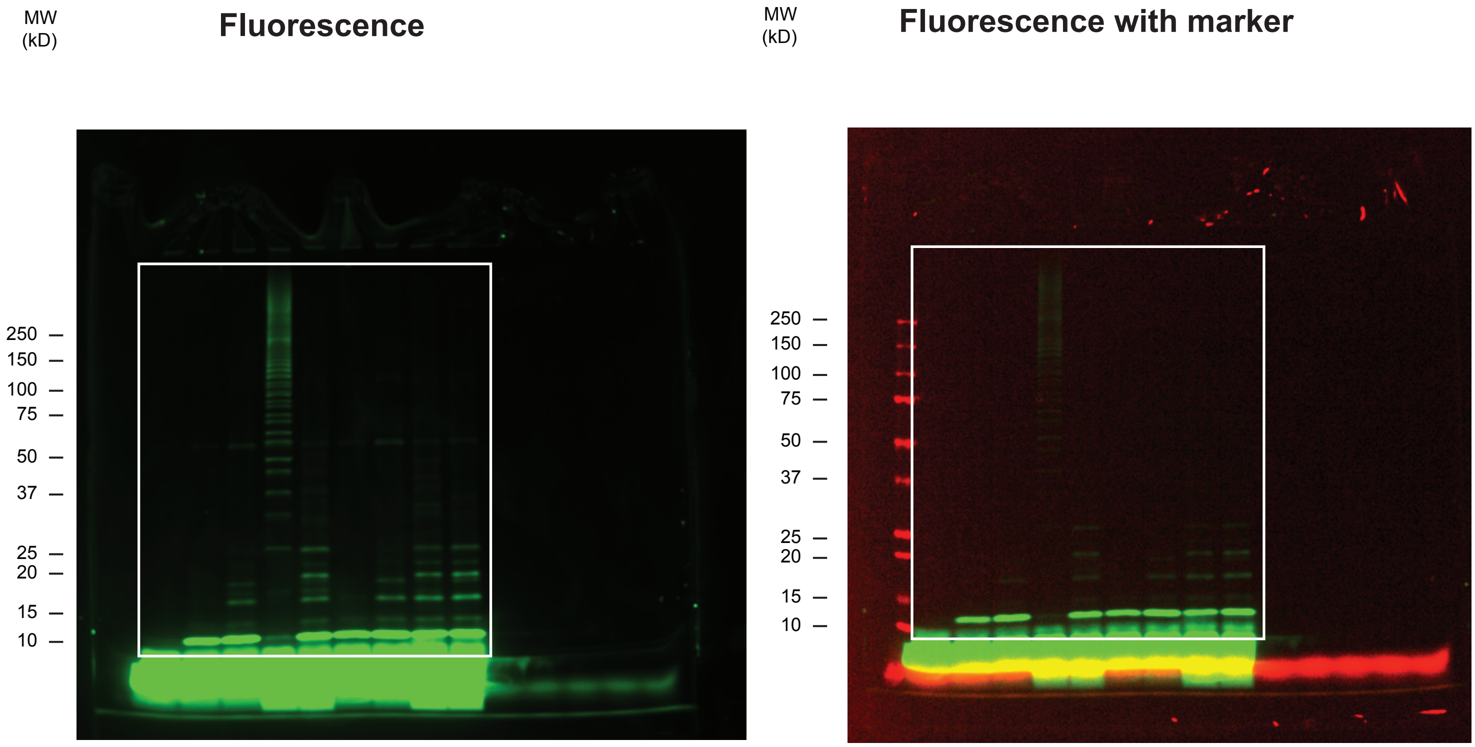

Extended Data Figure 9h

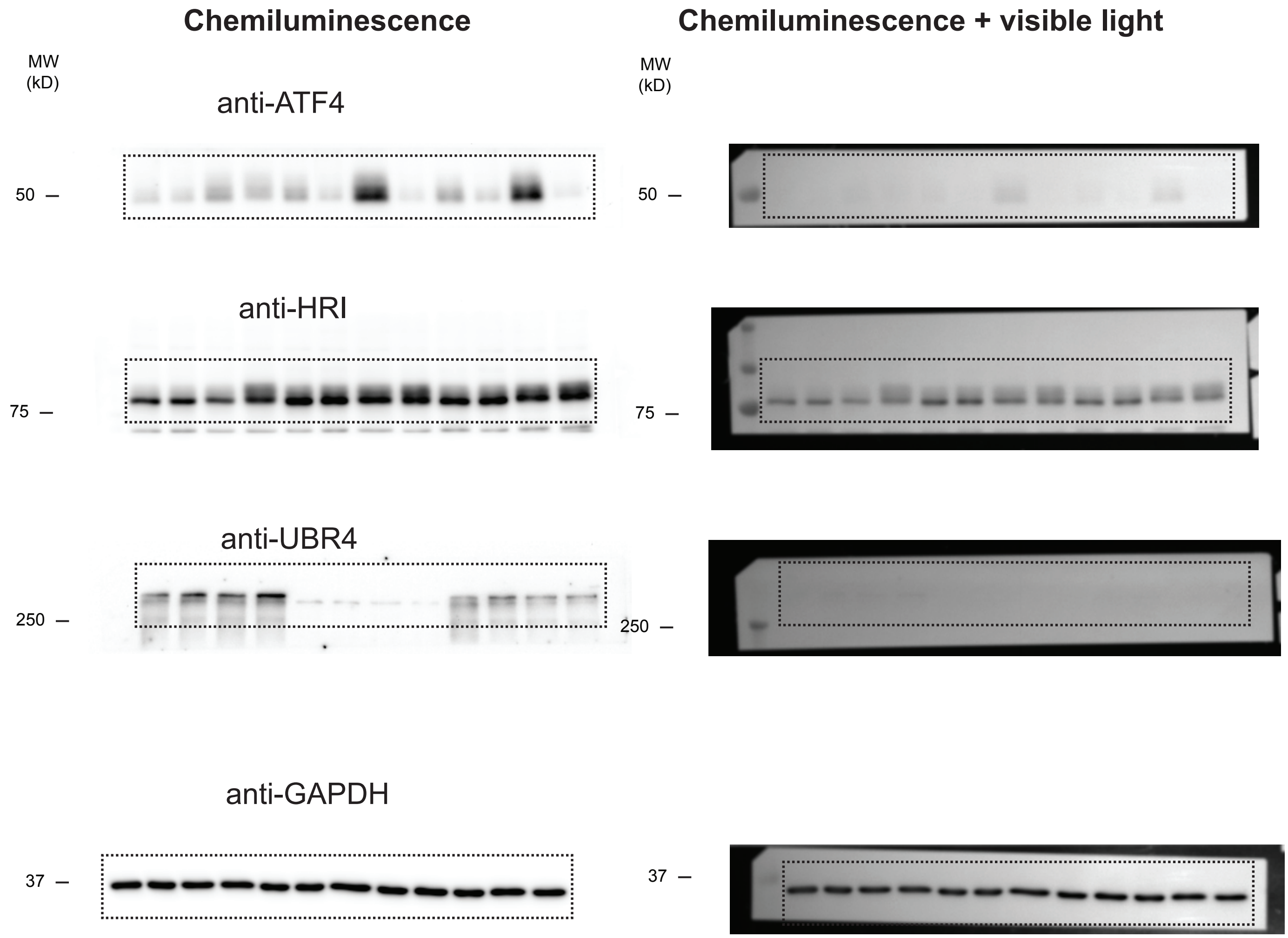

The loading control GAPDH was run on the same gel as ATF4, UBR4 and HRI.
