# Supplementary material for: Molecular basis of SIFI activity in the integrated stress response
Source: Nature. 2025 May 6;643(8073):1117–26. doi: 10.1038/s41586-025-09074-z (PMC12286842; doi:10.1038/s41586-025-09074-z)
Supplement: Supplementary file 2 — Reporting Summary [file 41586_2025_9074_MOESM2_ESM.pdf]

nature portfolio

Corresponding author(s): Michael Rapé

Last updated by author(s): 04/17/2025

Reporting Summary

Nature Portfolio wishes to improve the reproducibility of the work that we publish. This form provides structure for consistency and transparency in reporting. For further information on Nature Portfolio policies, see our Editorial Policies and the Editorial Policy Checklist.

Statistics

For all statistical analyses, confirm that the following items are present in the figure legend, table legend, main text, or Methods section.

n/a	Confirmed	
☐	☒	The exact sample size (*n*) for each experimental group/condition, given as a discrete number and unit of measurement
☐	☒	A statement on whether measurements were taken from distinct samples or whether the same sample was measured repeatedly
☒	☐	The statistical test(s) used AND whether they are one- or two-sided<br>*Only common tests should be described solely by name; describe more complex techniques in the Methods section.*
☒	☐	A description of all covariates tested
☒	☐	A description of any assumptions or corrections, such as tests of normality and adjustment for multiple comparisons
☐	☒	A full description of the statistical parameters including central tendency (e.g. means) or other basic estimates (e.g. regression coefficient) AND variation (e.g. standard deviation) or associated estimates of uncertainty (e.g. confidence intervals)
☒	☐	For null hypothesis testing, the test statistic (e.g. *F*, *t*, *r*) with confidence intervals, effect sizes, degrees of freedom and *P* value noted<br>*Give P values as exact values whenever suitable.*
☒	☐	For Bayesian analysis, information on the choice of priors and Markov chain Monte Carlo settings
☒	☐	For hierarchical and complex designs, identification of the appropriate level for tests and full reporting of outcomes
☒	☐	Estimates of effect sizes (e.g. Cohen's *d*, Pearson's *r*), indicating how they were calculated

Our web collection on statistics for biologists contains articles on many of the points above.

Software and code

Policy information about availability of computer code

Data collection	All software used is freely/commercially available: FACSDiva (Version 9.0)
Data analysis	All software used is freely/commercially available: FlowJo (Version 10.10.0), GraphPad Prism (Version 10), SerialEM (Version 4.1), AlphaFold 2 and AlphaFold 3, Coot (Version 0.9.8.92), ChimeraX (Version 1.8), Chimera (Version 1.17.1), PyMOL (Version 2.5.5), PHENIX (Version 1.21.1-5286), cryoSPARC (Version 4.3), PDBePISA (Version 1.52), NMRViewJ (Version 9.2.0-b27), Bruker TopSpin (Version 4.3.0), Spectronaut (Version 18.0), ProteoWizard's msConvert (v.3.0.22335), Kojak (v.2.0.3), Percolator (v.2.08), ProXL web application

For manuscripts utilizing custom algorithms or software that are central to the research but not yet described in published literature, software must be made available to editors and reviewers. We strongly encourage code deposition in a community repository (e.g. GitHub). See the Nature Portfolio guidelines for submitting code & software for further information.

Data

Policy information about availability of data

All manuscripts must include a data availability statement. This statement should provide the following information, where applicable:

- Accession codes, unique identifiers, or web links for publicly available datasets
- A description of any restrictions on data availability
- For clinical datasets or third party data, please ensure that the statement adheres to our policy

The consensus atomic coordinate model and cryo-EM map of the human UBR4-KCMF1-CaM (SIFI) complex has been deposited in the Protein Data Bank (PDB) and

Electron Microscopy Data Bank (EMDB) under the accession code 9D9Z and EMD-46686, respectively. Coordinates and cryo-EM maps of the endogenous SIFI complex (C-terminal partial map with improved local densities) has been deposited under accession codes PDB 9NWE and EMD-49876, and the SIFI complex purified through affinity-tagged KCMF1 (N-terminal partial map) have been deposited with accession codes PDB 9NWD and EMD-46688. The atomic model and cryo-EM map of the SIFI complex supplemented with UBE2A have been deposited under the EMDB accession code EMD-46742. Source data for immunoblots are provided in Supplementary Fig. 1. Gating strategies for flow cytometry experiments are provided in Supplementary Fig. 2. The workflow for cryo-EM structure generation is provided in Supplementary Fig. 3. Source data for the IP-MS datasets are provided in Supplementary Table 3. Cross-linking mass-spectrometry data is available interactively on the ProXL web application 73 https://proxl.yeastrc.org/proxl/p/sifi-hri along with the raw MS spectra and search parameters used. In addition, complete search algorithm configuration files, fasta search databases, raw search output, and raw MS data files were deposited to the ProteomeXchange Consortium via the PRIDE partner repository (https://www.ebi.ac.uk/pride/archive) with the dataset identifier PXD055759. The publicly available CRAPome and Mitocarta 3.0 datasets can be accessed at https://reprint-apms.org and https://www.broadinstitute.org respectively. There are no restrictions on data availability.

Research involving human participants, their data, or biological material

Policy information about studies with human participants or human data. See also policy information about sex, gender (identity/presentation), and sexual orientation and race, ethnicity and racism.

Reporting on sex and gender	This study does not involve human participants, their data or their biological material
Reporting on race, ethnicity, or other socially relevant groupings	This study does not involve human participants, their data or their biological material
Population characteristics	This study does not involve human participants, their data or their biological material
Recruitment	This study does not involve human participants, their data or their biological material
Ethics oversight	This study does not involve human participants, their data or their biological material

Note that full information on the approval of the study protocol must also be provided in the manuscript.

Field-specific reporting

Please select the one below that is the best fit for your research. If you are not sure, read the appropriate sections before making your selection.

☒ Life sciences ☐ Behavioural & social sciences ☐ Ecological, evolutionary & environmental sciences

For a reference copy of the document with all sections, see nature.com/documents/nr-reporting-summary-flat.pdf

Life sciences study design

All studies must disclose on these points even when the disclosure is negative.

Sample size	No methods to predetermine sample size for experiments were used. Sample sizes were chosen based on data from previous publications (Haakonsen et al., Nature 2024; Oh et al., Nature 2020; Yau et al., Cell 2017), robustness of the assay and technical and economical considerations.
Data exclusions	No data were excluded.
Replication	Biological replicates were performed and have been indicated in the figure legends as independent experiments. For experiments without biological replicates, the hypothesis was validated using an alternative experimental setup (different technique, ...) to address the same question. This is also stated in the corresponding figure legends.
Randomization	Not applicable, there was no subjective rating of data involved in our study. Randomization is not applicable for most standard cell culture based assays and in vitro biochemical experiments.
Blinding	Not applicable, there was no subjective rating of data involved in our study.

Reporting for specific materials, systems and methods

We require information from authors about some types of materials, experimental systems and methods used in many studies. Here, indicate whether each material, system or method listed is relevant to your study. If you are not sure if a list item applies to your research, read the appropriate section before selecting a response.

Materials & experimental systems

n/a	Involved in the study	
☐	☒	Antibodies
☐	☒	Eukaryotic cell lines
☒	☐	Palaeontology and archaeology
☒	☐	Animals and other organisms
☒	☐	Clinical data
☒	☐	Dual use research of concern
☒	☐	Plants

Methods

n/a	Involved in the study	
☒	☐	ChIP-seq
☐	☒	Flow cytometry
☒	☐	MRI-based neuroimaging

Antibodies

Antibodies used	Following antibodies were used for immunoblot analyses: anti-Flag (mouse, Clone M2, Sigma-Aldrich, F1804, dilution 1:1000), anti-Flag (rabbit, Cell Signaling Technology (CST), 2368, dilution 1:1000), anti-HA-Tag (rabbit, C29F4, CST, 3724, dilution 1:1000), anti-strep (mouse, strepMAB-Classic, 2-1507-001, iba lifesciences, dilution 1:10000), anti-GAPDH (rabbit, D16H11, CST, 5174, dilution 1:1000), anti-HSP90β (rabbit, D3F2, CST, 7411, dilution 1:1000), anti-α Tubulin (mouse, DM1A, Calbiochem, CP06, dilution 1:1000), anti-UBR4/p600 (rabbit, A302, Bethyl, A302-279A, dilution 1:1000), anti-UBE2A/B (mouse, G-9, Santa Cruz, sc-365507, dilution 1:150), anti-ATF4 (rabbit, D4B8, CST, 11815S, dilution 1:1000), anti-EIF2AK1 (rabbit, Proteintech, 20499-1-AP, dilution 1:1000), anti-KCMF1 (rabbit, Sigma, HPA030383, dilution 1:1000), anti-NIPSNAP3A (rabbit, ThermoFisher, PA5-20657, dilution 1:1000), anti-Ubiquitin (rabbit, Cell Signaling Technology (CST), 43124, dilution 1:1000), anti-ABHD10 (rabbit, ThermoFisher, PA5-103553, dilution 1:1000), goat anti-rabbit IgG (H+L) HRP (Vector Laboratories, PI-1000, dilution 1:5000), Sheep anti-mouse IgG (H+L) HRP (Sigma, A5906, dilution 1:5000), goat anti-mouse IgG light chain specific HRP conjugated (Jackson Immunoresearch, 115-035-174, dilution 1:5000).
Validation	Antibodies validated by knockdown/-out: anti-UBR4/p600 (rabbit, A302, Bethyl, A302-279A, validated for WB in human cells, see Haakonsen et al., 2024), anti-EIF2AK1 (rabbit, Proteintech, 20499-1-AP, validated for WB in human cells, see see Haakonsen et al., 2024), anti-KCMF1 (rabbit, Sigma, HPA030383, validated for WB in human cells, see see Haakonsen et al., 2024).<br><br>Antibodies validated by manufacturer: anti-Flag (mouse, Clone M2, Sigma-Aldrich, F1804, https://www.sigmaaldrich.com/US/en/product/sigma/f1804, used in 8252 publications, previously validated in our lab on recombinant proteins), anti-Flag (rabbit, Cell Signaling Technology (CST), 2368, https://www.cellsignal.com/products/primary-antibodies/dykddddk-tag-antibody-binds-to-same-epitope-as-sigma-s-anti-flag-m2-antibody/2368, used in 722 publications, previously validated in our lab on recombinant proteins), anti-HA-Tag (rabbit, C29F4, CST, 3724, https://www.cellsignal.com/products/primary-antibodies/ha-tag-c29f4-rabbit-mab/3724, used in 2406 publications, previously validated in our lab on recombinant proteins), anti-strep (mouse, strepMAB-Classic, 2-1507-001, iba lifesciences, https://www.iba-lifesciences.com/strepmab-classic/2-1507-001, used in 303 publications, detected correct protein size in this study), anti-GAPDH (rabbit, D16H11, CST, 5174, https://www.cellsignal.com/products/primary-antibodies/gapdh-d16h11-xp-rabbit-mab/5174, used in 5800 publications, detected protein at correct size in this study), anti-HSP90β (rabbit, D3F2, CST, 7411, https://www.cellsignal.com/products/primary-antibodies/hsp90b-d3f2-rabbit-mab/7411, validated by manufacturer for WB use, used in >10 publications, detected correct protein size in our study), anti-α Tubulin (mouse, DM1A, Calbiochem, CP06, https://www.sigmaaldrich.com/US/en/product/mm/cp06, used in 673 publications, detected protein correct size in this study), anti-UBE2A/B (mouse, G-9, Santa Cruz, sc-365507, https://www.scbt.com/p/ube2a-b-antibody-g-9, used in >10 publications, detected protein at correct size in this study), anti-ATF4 (rabbit, D4B8, CST, 11815S, https://www.cellsignal.com/products/primary-antibodies/atf-4-d4b8-rabbit-mab/11815, used in 703 publications, detected protein at correct size and induced upon ISR induction in this study), anti-NIPSNAP3A (rabbit, ThermoFisher, PA5-20657, https://www.thermofisher.com/antibody/product/NIPSNAP3A-Antibody-Polyclonal/10751-1-AP, validated by manufacturer for IP, IF, WB and IHC, detected protein at correct size in this study), anti-Ubiquitin (rabbit, Cell Signaling Technology (CST), 43124,https://www.cellsignal.com/products/primary-antibodies/ubiquitin-e4i2j-rabbit-mab/43124, used in 42 publications, recognizes endogenous levels of free ubiquitin and polyubiquitinated proteins. This antibody is able to detect free ubiquitin, linear polyubiquitin (M1-linked), and homotypic polyubiquitin chains consisting of K6, K11, K27, K29, K33, K48 and K63 linkages. validated by manufacturer for western blotting applications), anti-ABHD10 (rabbit, ThermoFisher, PA5-103553, https://www.thermofisher.com/antibody/product/ABHD10-Antibody-Polyclonal/PA5-103553, validated by manufacturer for WB, IHC, ICC/IF, detected correct protein size in our study)

Eukaryotic cell lines

Policy information about cell lines and Sex and Gender in Research

Cell line source(s)	HEK293T were purchased from the Berkeley Cell Culture Facility.
Authentication	All cell lines were authenticated by short tandem repeat analysis before freezing stocks (Feb 09, 2022)
Mycoplasma contamination	All cell lines were routinely tested biweekly for mycoplasma contamination using the Mycoplasma PCR Detection Kit (abm, G238) and consistently tested negative.
Commonly misidentified lines (See ICLAC register)	No commonly misidentified cell lines were used in this study.

Plants

Seed stocks	*Report on the source of all seed stocks or other plant material used. If applicable, state the seed stock centre and catalogue number. If plant specimens were collected from the field, describe the collection location, date and sampling procedures.*
Novel plant genotypes	*Describe the methods by which all novel plant genotypes were produced. This includes those generated by transgenic approaches, gene editing, chemical/radiation-based mutagenesis and hybridization. For transgenic lines, describe the transformation method, the number of independent lines analyzed and the generation upon which experiments were performed. For gene-edited lines, describe the editor used, the endogenous sequence targeted for editing, the targeting guide RNA sequence (if applicable) and how the editor was applied.*
Authentication	*Describe any authentication procedures for each seed stock used or novel genotype generated. Describe any experiments used to assess the effect of a mutation and, where applicable, how potential secondary effects (e.g. second site T-DNA insertions, mosiacism, off-target gene editing) were examined.*

Flow Cytometry

Plots

Confirm that:

- [x] The axis labels state the marker and fluorochrome used (e.g. CD4-FITC).
- [x] The axis scales are clearly visible. Include numbers along axes only for bottom left plot of group (a 'group' is an analysis of identical markers).
- [x] All plots are contour plots with outliers or pseudocolor plots.
- [x] A numerical value for number of cells or percentage (with statistics) is provided.

Methodology

Sample preparation	HEK293T cells were trypsinized and resuspended in PBS for flow cytometry.
Instrument	BD LSR Fortessa, BD LSR Fortessa X20
Software	FACSDiva (Version 9.0), FlowJo (Version 10.10.0)
Cell population abundance	For all assays, cells populations were determined using fluorescent markers. Non-fluorescent populations were clearly separated from those with fluorescent markers. Sufficient events were ensured for all populations.
Gating strategy	Initial gating steps include identification of live cells (SSC-A/FSC-A) followed by identification of single cells (FSC-H/FSC-A). The further gating strategy depends on the experimental setup:<br>- For cell competition assays mCherry+ and GFP+ populations were determined and ratios were calculated.<br>- For protein stability assays a derived parameter (GFP/mCherry) was plotted as a histogram to the mode within the GFP+/ mCherry+ population to represent stability of GFP-tagged proteins controlled by mCherry expression ensured an IRES.

- [x] Tick this box to confirm that a figure exemplifying the gating strategy is provided in the Supplementary Information.
