# Supplementary material for: Molecular basis of SIFI activity in the integrated stress response
Source: Nature. 2025 May 6;643(8073):1117–26. doi: 10.1038/s41586-025-09074-z (PMC12286842; doi:10.1038/s41586-025-09074-z)
Supplement: Supplementary file 3 — Gating strategy for flow cytometry experiments. [file 41586_2025_9074_MOESM3_ESM.pdf]

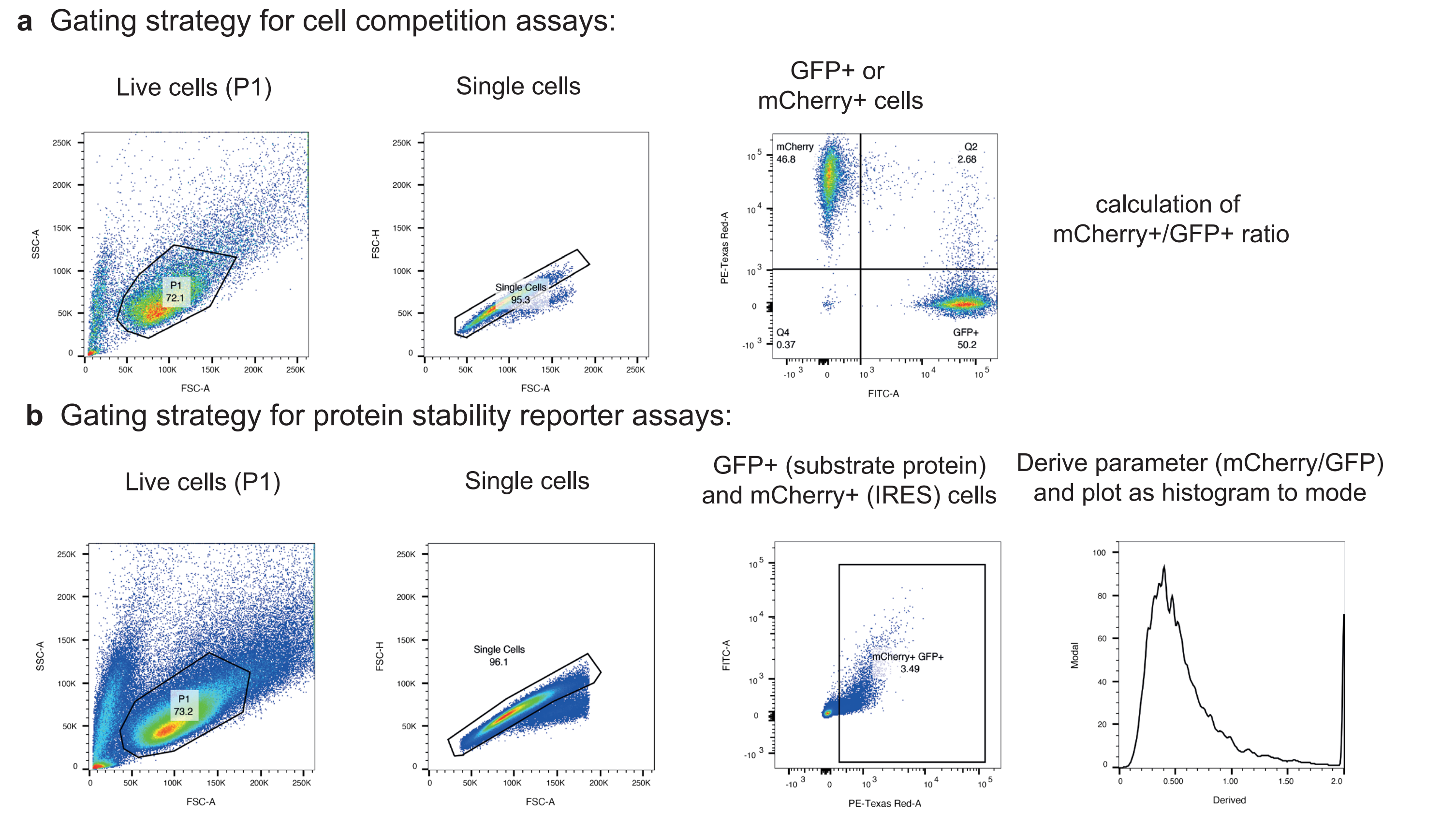

Supplementary Figure 2. Gating strategy for flow cytometry experiments. Representative plots shown for each type of flow cytometry analysis in this study. **a.** Gating strategy for cell competition assays. **b.** Gating strategy for protein stability reporter assays.
