# Supplementary material for: Molecular basis of SIFI activity in the integrated stress response
Source: Nature. 2025 May 6;643(8073):1117–26. doi: 10.1038/s41586-025-09074-z (PMC12286842; doi:10.1038/s41586-025-09074-z)
Supplement: Supplementary file 4 — Workflow of EM processing. [file 41586_2025_9074_MOESM4_ESM.pdf]

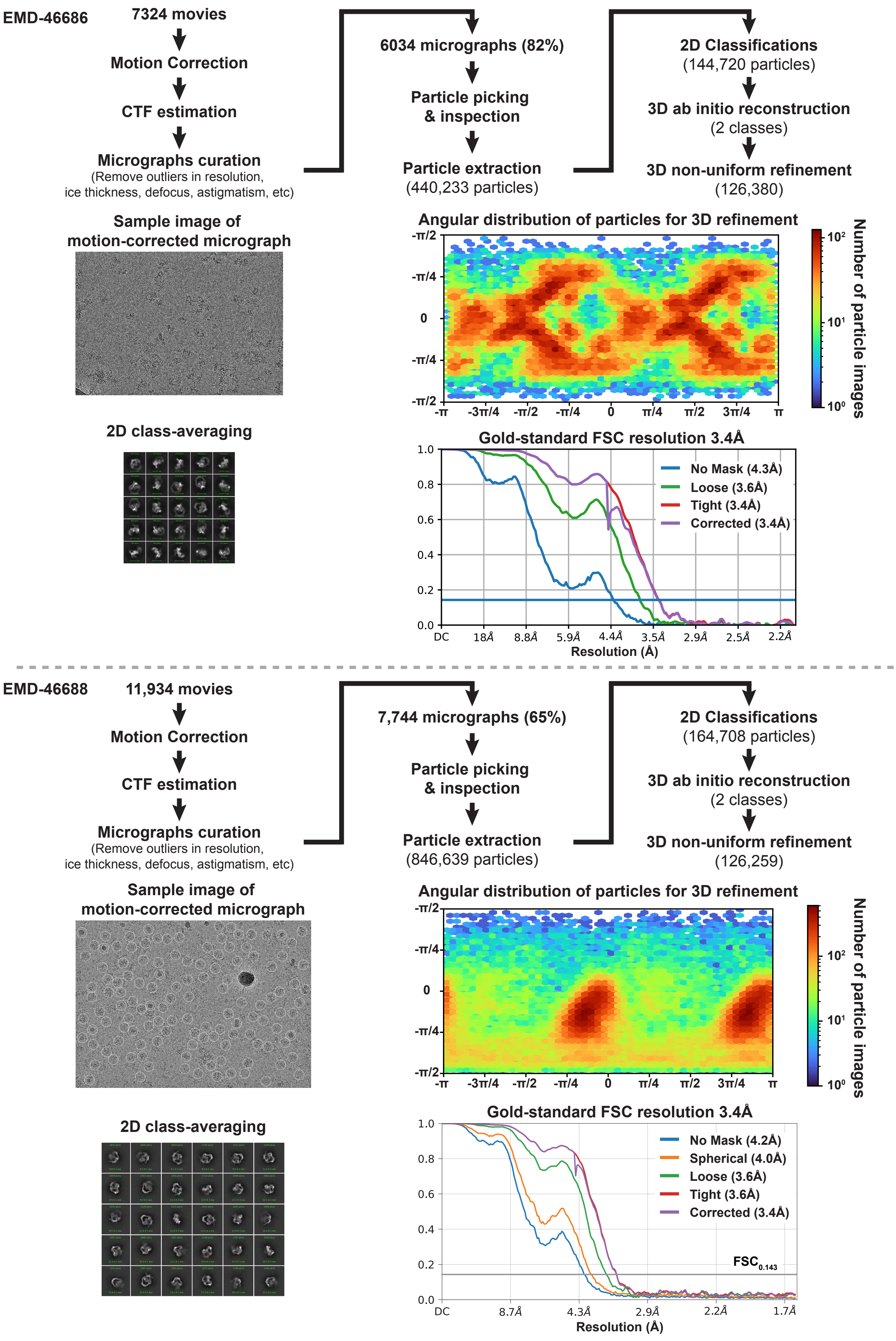

Supplementary Figure 3. Workflow for Cryo-EM data processing

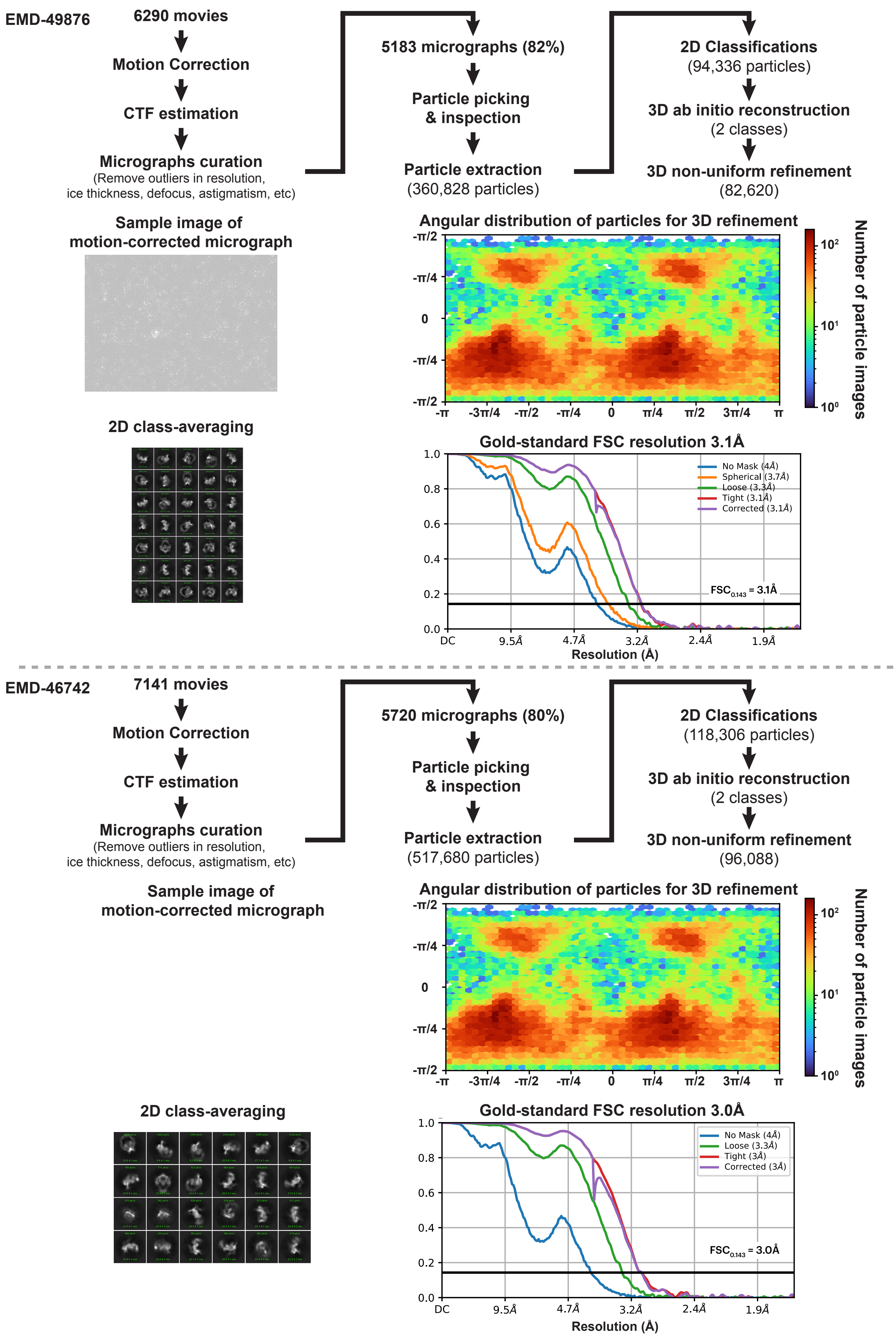
EMD-49876
6290 movies
Motion Correction
CTF estimation
Micrographs curation
(Remove outliers in resolution, ice thickness, defocus, astigmatism, etc)
5183 micrographs (82%)
Particle picking & inspection
Particle extraction
(360,828 particles)
2D Classifications
(94,336 particles)
3D ab initio reconstruction
(2 classes)
3D non-uniform refinement
(82,620)
Sample image of motion-corrected micrograph
Angular distribution of particles for 3D refinement
-π/2
-π/4
0
-π/4
-π/2
-π
-3π/4
-π/2
-π/4
0
π/4
π/2
3π/4
π
10^2
10^1
10^0
Number of particle images
2D class-averaging
Gold-standard FSC resolution 3.1Å
1.0
0.8
0.6
0.4
0.2
0.0
No Mask (4Å)
Spherical (3.7Å)
Loose (3.3Å)
Tight (3.1Å)
Corrected (3.1Å)
$FSC_{0.143}$ = 3.1Å
DC
9.5Å
4.7Å
3.2Å
2.4Å
1.9Å
Resolution (Å)
EMD-46742
7141 movies
Motion Correction
CTF estimation
Micrographs curation
(Remove outliers in resolution, ice thickness, defocus, astigmatism, etc)
5720 micrographs (80%)
Particle picking & inspection
Particle extraction
(517,680 particles)
2D Classifications
(118,306 particles)
3D ab initio reconstruction
(2 classes)
3D non-uniform refinement
(96,088)
Sample image of motion-corrected micrograph
Angular distribution of particles for 3D refinement
-π/2
-π/4
0
-π/4
-π/2
-π
-3π/4
-π/2
-π/4
0
π/4
π/2
3π/4
π
10^2
10^1
10^0
Number of particle images
2D class-averaging
Gold-standard FSC resolution 3.0Å
1.0
0.8
0.6
0.4
0.2
0.0
No Mask (4Å)
Loose (3.3Å)
Tight (3Å)
Corrected (3Å)
$FSC_{0.143}$ = 3.0Å
DC
9.5Å
4.7Å
3.2Å
2.4Å
1.9Å
Resolution (Å)
